# Exploring patient and family involvement in the lifecycle of an orphan drug: a scoping review

**DOI:** 10.1186/s13023-017-0738-6

**Published:** 2017-12-22

**Authors:** Andrea Young, Devidas Menon, Jackie Street, Walla Al-Hertani, Tania Stafinski

**Affiliations:** 1 Health Technology & Policy Unit, School of Public Health, University of Alberta, Edmonton, AB Canada; 2 School of Public Health, University of Adelaide, Adelaide, Australia; 3 Cumming School of Medicine, University of Calgary, Calgary, AB Canada

**Keywords:** Rare diseases, Orphan drugs, Patient involvement, Scoping review

## Abstract

**Background:**

Patients and their families have become more active in healthcare systems and research. The value of patient involvement is particularly relevant in the area of rare diseases, where patients face delayed diagnoses and limited access to effective therapies due to the high level of uncertainty in market approval and reimbursement decisions. It has been suggested that patient involvement may help to reduce some of these uncertainties. This review explored existing and proposed roles for patients, families, and patient organizations at each stage of the lifecycle of therapies for rare diseases (i.e., orphan drug lifecycle).

**Methods:**

A scoping review was conducted using methods outlined by Arksey and O’Malley. To validate the findings from the literature and identify any additional opportunities that were missed, a consultative webinar was conducted with members of the Patient and Caregiver Liaison Group of a Canadian research network.

**Results:**

Existing and proposed opportunities for involving patients, families, and patient organizations were reported throughout the orphan drug lifecycle and fell into 12 themes: research outside of clinical trials; clinical trials; patient reported outcomes measures; patient registries and biorepositories; education; advocacy and awareness; conferences and workshops; patient care and support; patient organization development; regulatory decision-making; and reimbursement decision-making. Existing opportunities were not described in sufficient detail to allow for the level of involvement to be assessed. Additionally, no information on the impact of involvement within specific opportunities was found. Based on feedback from patients and families, documentation of existing opportunities within Canada is poor.

**Conclusions:**

Opportunities for patient, family, and patient organization involvement exist throughout the orphan drug lifecycle. However, based on the information found, it is not possible to determine which opportunities would be most effective at each stage.

**Electronic supplementary material:**

The online version of this article (10.1186/s13023-017-0738-6) contains supplementary material, which is available to authorized users.

## Background

In recent years, patients have become active in healthcare systems and research, which have increasingly recognized the benefits of their involvement [1]. Such benefits include: improving the credibility and relevance of research [2]; enhancing the translation of research results into clinical practice; establishing more responsive services leading to better outcomes of care [3]; and identifying benefits and costs often missing from health technology assessments of new therapies [4]. While recognized by all patient communities, they are particularly valued by those with rare diseases, who experience significant challenges around their care. Often, they face delayed diagnoses and limited access to effective therapies, since decisions on market approval and reimbursement are fraught with uncertainties [5]. These uncertainties arise from a lack of robust information around 1) clinical benefit, 2) value for money, 3) potential adoption/diffusion, and 4) affordability [6]. High quality clinical trials are difficult to conduct in small patient populations where validated outcome measures are lacking [7], and natural histories are poorly understood [8, 9]. These therapies can also be extremely expensive, but in many cases, they represent the sole active treatment option for conditions that are not only rare, but also life-threatening or severely debilitating.

**Fig. 1:**
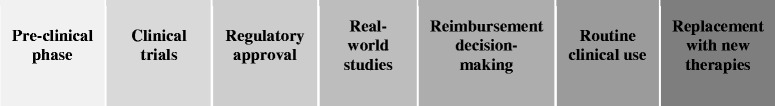
The Lifecycle of a Drug

It has been argued that patients can play an important role in reducing decision uncertainties [10]. In Canada, the potential value of patient input is reflected in Health Canada’s draft Orphan Drug Regulatory Framework, which was originally proposed in 2012. The Framework had two objectives: 1) providing Canadians with better, timelier access to orphan drugs (i.e., drugs that treat rare diseases) and 2) encouraging and facilitating clinical research on rare diseases [5], both of which were to be accomplished, in part, by incorporating patient involvement throughout the orphan drug lifecycle (Fig. 1) [5]. However, the ways in which patients were to be involved were not specified.

## Objective

The objective of this review was to explore existing and proposed roles for patients with rare diseases, their families, and patient organizations who represent them at each stage of the orphan drug lifecycle.

## Methods

A scoping review was conducted using the following 6 steps developed by Arksey and O’Malley [11, 12].

### Identifying the research questions

Two research questions were developed: 1) what opportunities exist for patients, families, and patient organizations to become involved at each stage of the orphan drug lifecycle, and 2) what opportunities have been proposed for involving them? In this paper, ‘patient’ refers to an individual living with a rare disease and ‘family’ refers to a patient’s family member or loved one who provides physical and emotional care.

### Identifying relevant studies

#### Literature search

A search for relevant peer-reviewed literature published in English between January 2000 and May 2017 was conducted using the following databases: MEDLINE, PubMed, The Cochrane Library, the Centre for Reviews and Dissemination databases (DARE, NHS EED and HTA), EMBASE, Web of Science, and EconLit. Search terms included relevant controlled vocabulary (e.g., Medical Subject Headings (MeSH): Patient Preference, Patient Participation, Consumer Participation) and keywords (e.g., Patient Engagement and Patient Involvement). These were combined with terms for medical technologies (e.g., MeSH terms: Diffusion of Innovation and Biomedical Technology), as well as rare diseases (Rare Diseases and Orphan Drug Production). A search for grey literature was also conducted using ProQuest Dissertations and Theses, NHS Evidence, and Google. The electronic searches were supplemented by a manual search of reference lists of relevant papers. Full details of the search terms and sources used are provided in (see Additional file 1: Appendix A).

#### Review of regulatory and reimbursement decision-making processes

Over the past decade, opportunities for and public awareness of patient input into regulatory and reimbursement processes (e.g., patient evidence submissions; membership on advisory or decision-making committees) have increased considerably. To capture such opportunities, the websites of regulatory and reimbursement decision-making bodies in 20 countries were reviewed. They represented the top 20 Organization for Economic Co-operation and Development (OECD) countries based on gross domestic product (GDP) per capita with populations greater than 1 million people and socialized health insurance programs/universal healthcare; thus, they were seen as relevant to the Canadian context [13]. Four countries (Qatar, Kuwait, the United Arab Emirates, and Israel) were initially included in the review, but a preliminary search revealed a paucity of information regarding decision-making processes on drugs in these countries. They were replaced with the next four OECD countries that fulfilled the inclusion criteria.

Twelve of the countries are members of the European Union (EU), which hosts the European Medicines Agency (EMA) [14]. Since it is compulsory for officially designated orphan drugs to go through EMA, its centralized regulatory approval processes were reviewed.

Searches for additional information on decision-making processes were performed using the Google search engine. When opportunities for involvement were not well described, emails were sent to the decision-making organizations to request clarification.

### Study selection

#### Literature eligibility criteria

Literature selection was completed by two reviewers who independently scanned the titles and abstracts of citations identified through the search. Studies were included if they described the involvement of patients, families, or patient organizations in any stage of the orphan drug lifecycle. Documents produced by patient organizations describing their work were also included.

Since this review focused on involvement of patients with rare diseases, their families and patient organizations throughout the orphan drug lifecycle, literature describing “public”, “community” and “citizen” engagement, non-drug technologies, or common diseases were excluded,. However, because a broad lifecycle approach was used, literature describing activities applicable to both drug and non-drug technologies (e.g. natural history registries) was included. Papers describing patient involvement in individual clinical decision-making with health care providers were excluded, since this study focused on macro-level processes. Abstracts, editorials and commentaries were excluded.

#### Decision-making process eligibility criteria

No eligibility criteria were applied to the review of decision-making processes and all decision-making processes identified were included in this review.

### Charting the data

One reviewer (AY) extracted data from all of the included papers using a standardized data extraction form. A second reviewer (TS) extracted data from a random sample (30%) of the included papers to assess reliability. For each ‘opportunity’ identified, the following information was extracted: description of the activity; country in which it took place; type of disease; participants involved (i.e., patients, families, or patient organizations); participants’ role; and the impact or outcome.

For each decision-making process identified, one reviewer (AY) extracted data on any aspects in which patients, families, or patient organizations were involved (e.g., submitting a topic for consideration; membership on advisory or decision-making committees). Each aspect was defined as an opportunity for involvement.

### Collating, summarising and reporting the results

Thematic analysis and open coding methods were used to explore the types of activities reported in each opportunity and identify categorical themes. Thematic analysis involves the identification, analysis and reporting of patterns through repeated handling of data [15]. Open coding is used to identify themes by breaking the data into distinct parts and examining and comparing them to identify similarities and differences [16]. Where there appeared to be overlap among themes, a decision was made to keep them separate if combining them would result in the loss of an important concept or idea. For example, the theme of “Conferences and Workshops” overlaps with “Advocacy and Awareness”. While ‘advocacy’ may take place at conferences, it is not necessarily the main purpose of such events. Opportunities were also categorized as either existing (i.e., has taken place) or proposed (i.e., is suggested for the future).

### Consultation exercise

Members of the Patient and Caregiver Liaison Group of a Canadian research network (Promoting Rare-Disease Innovations through Sustainable Mechanisms or PRISM [17]) were invited to participate in a webinar to validate the findings of the review and identify any additional opportunities that were missed*.* These individuals were either patients with rare disease patients or family members. The webinar began with a brief introduction and description of the review methodology. Subsequently, participants were presented with existing and proposed opportunities for patients, families and patient organizations (in that order) under each theme. They were invited to comment on the results and asked if they knew about any other existing or proposed opportunities. Following the webinar, participants were contacted individually for clarification or additional information as needed.

### Mapping opportunities onto the orphan drug lifecycle

Opportunities for involvement were mapped onto the orphan drug lifecycle by considering the stage at which each opportunity might take place. Two maps were created, one reflecting opportunities identified in the literature/website review and the second reflecting the additional opportunities identified in the webinar.

## Results

The results of the literature search and review of websites of regulatory and reimbursement processes are summarized in Appendices A and B, respectively (Additional file 1: Appendix A and Additional file 2: Appendix B). Seventy-three published studies, eleven grey literature documents, and sixty-six webpages were reviewed. Existing and proposed opportunities for involvement fell into 12 themes: research outside of clinical trials; clinical trials; patient reported outcome measures; patient registries and biorepositories; stakeholder relationships and collaborations; education; advocacy and awareness; conferences and workshops; patient care and support; patient organization development; regulatory decision-making; and reimbursement decision-making. Definitions of each theme can be found in Table C-1 (Additional file 3: Appendix C).

A summary of the most commonly identified opportunities follows.

### Existing opportunities for involvement in the orphan drug lifecycle (Table 1)

#### Research

Individual patients and their families have contributed to strategic directions for research through membership on research priority-setting committees and participation in priority-setting exercises [18]. They have also provided input into non-clinical studies aimed primarily at understanding the experience of patients and families living with rare diseases. In contrast, roles for patient organizations have mainly involved the collection and synthesis of information from their members (e.g. evaluating the effectiveness of new orphan drugs for organization members [19]).

**Table 1:** Existing opportunities for involvement identified in the literature and website review

Type of involvement	Number of Papers	Countries	Patients and families	Patient organizations
Research	33	• Australia • Canada • Netherlands • Germany • France • Spain • United States • United Kingdom • Internationally (i.e. 2+ countries)	• Participated as research subjects (International [132]; Denmark [133]; Japan [134]; Spain [93]; United Kingdom [94, 133, 135]; United States [35, 136]) • Set research priorities (Netherlands [137, 138]; Spain [18]) • Initiated research studies (United States [19, 57]) • Provided assistance to researchers conducting studies (Europe [139]); United States [140]) • Led research (International [53]; United States [19, 141]) • Developed or participated in research organizations/networks (Europe [142]; United States [143]) • Disseminated research-related information (International [144]; United Kingdom [54])	• Participated as research subjects (Europe [96]) • Set research priorities (Germany [42]; Netherlands [137]) • Initiated research (Netherlands [42]; United States [145]) • Provided assistance to researchers conducting studies (International [30, 146]; Netherlands [137]; Spain [93]; United States [19]) • Led research (Europe [45]; France [56]; Ireland [146]; United Kingdom [97]; United States [19, 24, 40, 141]) • Participated in research organizations/networks (Europe [142, 147]; Netherlands [42, 46]; United States [39, 47, 143, 148]) • Disseminated research-related information (International [144]; United Kingdom [54]) • Funded research (International [149]; Germany [42]; United States [34, 35, 40, 47, 49, 143])
Clinical Trials	8	• Netherlands • United Kingdom • United States • Europe	• Participation in trials [20]	• Provided assistance to researchers conducting trials (Europe [21]; Netherlands [46]; United States [21]) • Funded clinical trials and clinical trial networks (Europe [150]; United States [23]) • Established and/or participated in clinical trial networks (Europe [150]; United States [23, 24, 47]) • Disseminated information on the results of clinical trials (United States [23, 24])
PROMs	5	• Germany • United States • Internationally	• Submitted patient reported outcomes (PROs) in studies (International [32]) • Participated in studies to develop and validate outcome measures (International [26, 151]; Germany [27]; United States [28]) • Assisted researchers conducting studies to develop and validate outcome measures (International [26])	• Assisted researchers in conducting studies to develop and validate outcome measures (International [29])
Registries/ biorepositories	17	• Sweden • United States • Internationally	• Patients and/or families enrolled in and submitted data to registries/biorepositories (International [29, 37]; Sweden [33]; United Kingdom [152]; United States [24, 34–36]) • Provided input on registry/ biorepository design (International [32]; Europe [147]) • Involved in maintenance and/or management (Europe [153]) • Established registries (United States [41]); however, this was usually done through a patient organization	• Established of registries (International [32]; Europe [38]; United Kingdom [152]; United States [19, 34, 39–41]) • Provided input on registry/ biorepository design (International [32]; Europe [147]; United States [154, 155]) • Involved in maintenance and/or management (International [156]; Europe [38, 153]; Italy [157]; United Kingdom [152]) • Provided funding (Europe [153]; United Kingdom [152]; United States [154]) • Recruited participants (International [32, 156]; Italy [157]; United Kingdom [152])
Stakeholder relationships and collaborations	9	• Netherlands • United Kingdom • United States • Internationally	• Established and maintained relationships with researchers (Netherlands [42]; United States [35])	• Facilitated relationships between different stakeholders through charters for collaboration and hosting neutral meetings (Europe [21]; United States [47]) • Established relationships with stakeholders (e.g. researchers, industry, healthcare professionals, and other patient organizations) (European Union [96]; Italy [157]; Netherlands [42]; United States [23, 35, 40, 47])
Education	11	• Netherlands • United States • Internationally	• Helped to develop educational material and training programs for patients and families (Europe [43]; Italy [44])	• Shared informational resources on various disease-specific topics (Europe [45]; Netherlands [46]; United States [22–24, 40, 47]) • Organized and sponsored formal educational activities and training programs for healthcare professionals, researchers and policymakers (United States [22–24, 34, 49])
Advocacy and awareness	8	• Germany • United States • Europe	• Used social media to advocate for access to experimental drugs (United States [48]).	• Advocated for drug access and coverage (United States [22]) • Advocated for research (Europe [38]; Germany [42]) • Advocated for legislation (United States [47, 49]) • Started awareness campaigns (United States [22, 47])
Conferences and workshops	8	• Australia • United States	• Participated in conferences and workshops, helping to identify goals and produce recommendations for a national rare diseases strategy (Australia [95])	• Participated in conferences and workshops aimed at developing a national rare diseases strategy (Australia [95]) • Hosted and funded multi-stakeholder conferences and workshops (United States [23, 24, 34, 35, 40, 49])
Patient care and support	15	• Italy • United Kingdom • United States • Internationally	• Provided social support for other patients (International [53, 158]; United States [19, 140]) • Self-monitored clinical care through electronic health records (Italy [50]) • Provided clinical care support by contributing to the development of clinical practice guidelines ([51, 52]	• Provided social support (Europe [45]; United Kingdom [22, 49]; United States [22, 40]) • Provided financial support (United Kingdom [22]; United States [22, 47]) • Provided clinical care support (Europe [38, 45]; United States [22, 34, 40]) • Provided support to patients in clinical trials (Europe [21]; United States [47])
Patient organization development	5	• France • Netherlands • United States	• Established patient organizations, like the French Muscular Dystrophy Organization and Chromosome 18 Registry and Research Society (France [56]; United States [19])	• Provided advice to others on how to start an organization (United States [57]) • Established international patient organization alliances (International [156]; Netherlands [46]; United States [40]) • Further developed their organization by hosting fundraising events (United States [40])
Regulatory decision-making	5^a^	• Canada • New Zealand • Switzerland • United Kingdom • United States • European Union	• Submitted PROs for consideration by a regulatory body (Canada [65]; European Union [159]; New Zealand [160]; United States [161]) • Provided input on proposed regulatory decisions/guidelines (Canada [64, 65]; New Zealand [66]; United States [67, 68]) • Served as members on advisory/decision-making committees (Canada [64, 65]; United States [67, 68]) • Provided input into assessments of benefits and harms (United States [162]) • Reported adverse events to regulators (Australia [58]; Canada [59]; European Union [60]; New Zealand [61]; Switzerland [62]; United States [63])	• Representatives sat on advisory/decision-making committees (European Union [69, 70]) • Provided input on pre-submission advice given to researchers regarding clinical trial protocols (European Union [69]) • Provided input on assessments of benefits and harms (European Union [60]) Provided input on plans for ongoing pharmacovigilance (European Union [60]) • Provided input on consumer information, such as labelling (European Union [69])
Reimbursement decision-making	5^a^	• Australia • Canada • Denmark • Germany • Netherlands • New Zealand • Ontario (CAD) • Sweden • Switzerland • Scotland • United Kingdom • United States • Wales	*Centralized review processes* • Patients submitted drugs for evaluation (Australia [75]; New Zealand [163]) • Submitted information for use in evaluations, such as the degree of perceived benefit, subjective risk assessment, or burden of associated side effects (Netherlands [71]; New Zealand [72]; United States [73]) • Participated in consultations during the review process (Ontario [74]; New Zealand [72]; United States [73]) • Served as members on advisory/decision-making committees (Canada [164]; Netherlands [71]) • Provided feedback on completed evaluation reports or recommendations (New Zealand [72]; United States [165]) • Prepared patient submissions for consideration alongside clinical and economic evidence (Australia [75]; United Kingdom [76]; Wales [77]) • Presented views during review committee meetings (United Kingdom [87]; United States [73]) • Consulted on the design of the evaluation process (United Kingdom [97])	*Centralized review processes* • Reviewed horizon scanning reports (United Kingdom [166]) • Submitted drugs for evaluation (Australia [167]; New Zealand [163]) • Participated in consultations during the review process (Australia [168]; Germany [169]; United Kingdom [87]; Scotland [170]) • Served as members on advisory/decision-making committees (Sweden [171]; Switzerland [172]; United Kingdom [173]; United States [174]) • Prepared patient submissions for consideration alongside clinical and economic evidence (Australia [75]; Canada [175]; Ontario [74]; United Kingdom [176]; Scotland [177]; Wales [77]) • Provided feedback on completed evaluation reports or recommendations (Ontario [74]) • Launched appeals of negative funding decisions (United Kingdom [97]) • Created recommendations for the design of the evaluation process (United Kingdom [97])
			*Safety-net review processes* • Submitted drugs for consideration (Finland [90]) • Directly consulted during the review process (United Kingdom [87]) • Provided feedback on evaluations or recommendations (United Kingdom [87]) • Presented views during a committee meeting (United Kingdom [87]) • Submit data for use in annual evaluations for reapplications (Australia [178])	

^a^In addition to the website review

#### Clinical trials

Patient opportunities have solely comprised enrollment in clinical trials as study subjects [20]. However, patient organizations have contributed in multiple ways, including providing input into trial design and helping to recruit trial participants through communication of trial information to members [21–24].

#### Patient reported outcome measures

Patient reported outcome measures (PROMs) are self-reported measurement instruments designed to provide information on aspects of health status relevant to a patient’s quality of life, including symptoms, functionality, and physical, mental, and social health [25]. To date, the role of patients and families in PROMs has predominantly been in the development and validation of such measures (e.g. consulting patients when formulating questions for PROM survey [26–28]). Patient organizations have then managed the distribution of such surveys to patients and families [29].

#### Patient registries and biorepositories

Patients and families have contributed to registries (predominantly natural history) through their enrollment and submission of data [24, 30–37]. Many of the registries have been established by patient organizations, in collaboration with researchers and healthcare professionals [19, 32, 34, 37–41].

#### Stakeholder relationships and collaborations

On an individual level, patients and families have established informal relationships with researchers [35, 42]. In contrast, patient organizations have established formal relationships with stakeholders or facilitated relationships between stakeholder groups (e.g. by developing charters for collaboration [21]).

#### Education

Patients and families have helped develop training programs for patients/families by identifying key content areas [43, 44]. More opportunities were reported for organizations which have been involved in sharing informational resources with patients, families, and health care professionals, often through websites, toll-free hotlines, and mentoring programs [22–24, 40, 45–47]. They have also organized formal educational activities for families (e.g. parent training programs [44]) and health care professionals (e.g. Grand Rounds [22]).

#### Advocacy and awareness

While ‘advocacy’ by patients and families has mainly involved the use of social media to press for improved access to drugs [48], in patient organizations it has comprised a variety of efforts (e.g. lobbying for public funding; testifying before Congressional committees) spanning drug coverage [22], research [42] and legislation on rare diseases and orphan drugs [47, 49].

#### Conferences and workshops

Patients and families have participated in conferences and workshops (e.g. helping identify goals for a national rare disease strategy), while patient organizations have also hosted and funded them [23, 24, 34, 35, 40, 49].

#### Patient care and support

Some patients and families have been able to provide input into their care because they have had access and the ability to update, their electronic health records, which are reviewed by health care providers [50]. They have provided input into patient care more broadly through their participation in the development of clinical practice guidelines [51, 52].

With respect to social support, individual patients and families have built connections and shared information through online fora (e.g. Patients Like Me [53]) developed mostly by patient organizations (e.g. Muscular Dystrophy Charity [54]). Patient organizations have also provided social support through social events and mentoring programs [22, 40, 45, 49, 55].

#### Patient organization development

Patients and families have created patient organizations (e.g. French Muscular Dystrophy Organization [56]) which, in turn, have provided advice to others who wish to establish them [57].

#### Regulatory decision-making

Patients and families have mainly contributed indirectly to regulatory processes by reporting on adverse events [58–63] or providing input on proposed regulatory guidelines [64–68]. However, patient organizations have been more directly involved, with opportunities to serve on decision-making/advisory committees [69, 70].

#### Reimbursement decision-making

Opportunities for patient involvement in reimbursement decision-making have mainly focussed on patient organizations and included submission of information for use in evaluations (e.g. degree of perceived benefit, subjective risk assessment, or burden of associated side effects) [71–73], participation in consultations during the review process [72–74], and preparation of patient submissions for consideration alongside clinical and economic evidence [75–77]. Regarding patient submissions, similar processes exist in Australia, Canada, and the United Kingdom. In general, they involve completion of templates that ask about 1) how information in the submission was obtained, 2) impact of the disease on patients, 3) experiences of patients with their current therapy, 4) expectations patients/families for the new drug and 5) any patient experiences with the new drug [78, 79]. In Canada, the submissions also request information on family impact [78].

Opportunities for individual patients have also involved participation in safety-net programs, which provide patients with access to unapproved or non-reimbursed drugs on a case-by-case basis for a fixed time period [71, 80–89]. Individual patients and families have submitted drugs for consideration by such programs [90], participated in consultations during the review process [87], and presented their views during committee meetings [87].

Despite the wide range of existing opportunities identified, none were described in sufficient detail to determine the level of involvement they represented using published frameworks for engagement [91, 92]. These frameworks often present involvement on a spectrum, such as Arnstein’s ladder, which ranges from “manipulation” (i.e. nonparticipation) to “citizen control” (i.e. citizen power) [91]. Information on the impact of involvement in specific opportunities was also limited. One study reported, “[Patient involvement] was found to be particularly effective in enhancing the design and conduct of our research” [93]. The remaining studies typically commented on the success of an opportunity as whole (e.g. “the health utility values provided by this study could inform assessments of cost-effectiveness…” [94]).

### Proposed opportunities for involvement

Proposed opportunities for involvement are presented below. In general, they were described at a high level and lacked information on the *exact* way in which patients, families, or patient organizations should be involved.

#### Research

Participants attending the Australian Rare Disease Symposium suggested that patients and their families should be directly involved in all decisions about research on rare diseases, including involvement in the decision-making processes of research collaborations and networks [95].

#### Clinical trials

Participants of the European Haemophilia Consortium (EHC) Congress proposed that patients should be involved in ensuring clinical trials collect real-world outcomes that are meaningful to them [96]. In the United States, the National Organization for Rare Disorders (NORD) proposed that it should develop systems for improving patient access to and participation in trials [47]. Finally, the Genetic Alliance UK proposed that patient input on acceptable risk should be embedded into R&D of new treatments, from drug design to clinical trials [97].

#### Stakeholder relationships and collaborations

One proposed opportunity for patients in the establishment/ maintenance of relationships with stakeholders was identified. The Genetic Alliance UK has called for improved dialogue between patients, manufacturers, regulatory bodies, and clinical researchers to ensure alignment between the data required for decision-making and the data collected in clinical trials [97].

#### Patient care and support

The Genetic Alliance UK proposed that patients, in partnership with their physicians, should be responsible for deciding if the benefits of using a new drug outweigh the associated risks [97]. Families participating in a research study proposed that those who have taken part in clinical trials provide support for newly enrolled patients and families [98].

#### Regulatory decision-making

Three organizations proposed opportunities for involvement in regulatory decision-making. In the United Kingdom, the Genetic Alliance UK suggested that patient input on acceptable risk should be incorporated into decision-making [97]. In Europe, participants in the EHC Congress symposium proposed that patient representatives should be added to advisory committees that do not currently have representation [96]. In the United States, NORD proposed that it should be involved in 1) identifying laws, regulations and policies that need to be changed to encourage product approval [47] and 2) establishing greater certainty in the orphan product approval process, through consultations on trial design and endpoint selection [47].

#### Reimbursement decision-making

At the EHC Congress’s Symposium, it was suggested that patient input should be sought at an earlier stage in the health technology assessment (HTA) process [96] and that patient advocates and clinicians should work together to create a shared consensus on the evaluation of the efficacy and effectiveness of therapies [96]. In the United Kingdom, the Genetic Alliance UK proposed four potential roles for patient involvement in reimbursement decision-making: presenting to the evaluation committee, consulting on the development of post-evaluation research, providing input on any reassessment of risks and benefits, and contributing to topic selection during horizon scanning [97]. The Genetic Alliance UK also stated that patient organizations should be formally involved in drug identification and selection for reimbursement decision-making [97]. In the United States, NORD proposed that patient organizations work to assure reimbursement of off-label drug use for rare disease patients [47].

### Consultation exercise (feedback from patients and families)

Eight members of the PRISM Patient and Caregiver Liaison Group participated in a webinar, during which feedback on the above existing and proposed opportunities was sought. Members represented a variety of disease types (e.g. cancer; non-cancerous tumor; blood, metabolic; connective tissue; endocrine; lung; secretory gland; and epileptic encephalopathies) and a range of experience within their disease communities. Five participants were patients and three were family members. All eight were involved in patient organizations to some degree (from new members to holding executive positions). This allowed participants to speak from their perspective as patients/family members and members of patient organizations. Participants ranged in age from 18 to 70 years and there were two males and six females. The session lasted 2.5 h. In general, participants agreed with the findings of the review, but identified some opportunities that were not captured in the literature. They also clarified their role in some of the opportunities that were identified. These additions and clarifications are described below.

#### Research

Patients and families have fundraised for research, even when there is no organization to represent them. Patients, specifically, have also provided input on the format in which they would like to receive a new drug, should it come to market (e.g., pill vs. liquid).

#### Clinical trials

Patients and families differentiated between recruiting patients to clinical trials and providing them with enough information to make informed decisions on whether or not to participate. They felt that while they had a role to play in “*providing all the information necessary so… patients know what options are out there in terms of research” (Family member 2 (FM2))*, it did not include recruitment of participants.

#### Patient registries and biorepositories

Patients and families mentioned their involvement in raising funds to establish registries (without the support of an organization) and using social media (e.g., Facebook) to encourage enrollment. They also discussed some of the challenges they have experienced, such as cost:

*“…you’ve listed ‘establish and maintain registries’ but a lot of organizations do not do that because it is too expensive, too onerous.” – FM2.*

To address these challenges, organizations are considering different types of registries, some of which involve partnerships with industry or the academic community. They have encouraged their members to participate in registries established by industry and requested data from these registries to share with the disease community.

#### Stakeholder relationships and collaborations

Patients and families indicated that some have been very proactive about building relationships with stakeholders outside of a patient organization, (individuals *“can be the link between stakeholders” (Patient 4 (P4))* (e.g., linking fundraisers with patient organizations). They provided examples of their own relationships with the government through their local Members of Parliament (MPs) and Members of the Legislative Assembly (MLAs).

#### Education

Patients and families discussed a variety of work they have done *“as individual[s]… not through the patient organization” (FM1)* to provide education about their disease to, not only other patients and families, but also health care professionals. They have presented at Grand Rounds in academic medical centres and participated in standardized patient programs at universities, which has helped to familiarize students in health-related fields with different a range of rare medical conditions.

#### Advocacy and awareness

Patients and families believe *“…there’s a time and place for both [patients and patient organizations] to come in and [do advocacy]…individual patients have done a lot of individual advocacy work.” (P3).* They have advocated for access to new drugs well before social media existed (e.g., meeting with their local MPs) and for improved education, clinics, and patient care services. They have also taken part in campaigns hosted by research organizations, initiated their own campaigns (online and in schools), and hosted fundraising events.

With respect to patient organizations, they have provided guidance to patients and families around how to advocate for themselves, linked them with resources in their community and written letters of support.

#### Conferences and workshops

Patients and families indicated that at conferences and workshops, *“…you have the expert patients who can share their knowledge and give their perspective to other patients or even to doctors and healthcare providers” (P4)*. One participant had served on a planning committee and organized opportunities for patient involvement (e.g., sitting on a panel) to ensure that these opportunities were driven by actual patients. Another participant had volunteered at patient organization-hosted conferences, aiming to help the conference run more smoothly and increase involvement by assisting with aspects such as childcare.

#### Patient care and support

Patients and families felt that, as individuals, they provide not *“not just social support”* (FM3), but also clinical care support, helping other patients to identify appropriate treatments and dosing. Patient organizations also offer clinical care support through sharing information on treatments and services available locally in each province/territory.

#### Regulatory decision-making

Participants mentioned that they had participated in a webinar hosted by Health Canada, which described its plans to *“include patient involvement in regulatory decision-making”*. However, they *“didn’t think it had gotten very far on it…” (P3)*. No additional information on this project was found.

#### Reimbursement decision-making

Participants described their own experiences with patient evidence submissions to the common drug review (CDR) in Canada. Many felt that the weight given to patient submissions during decision-making, compared with other sources of information (e.g., clinical trial data) was unclear.

*“…I know very little about the weight it’s given. I don’t think anybody knows the weight it’s given” – FM2.*

Participants also described ways in which patient organizations have been involved in decision-making when the CDR issues a negative recommendation. These included media campaigns and meetings with government officials in several provinces.

#### Other

Participants discussed the importance of sharing information on specific ways in which they have been involved at the different stages of the orphan drug lifecycle:

*“…the patients and families who are involved in that have to be ambassadors and get out there and show the rest of the research community how it works and how it was successful and what the impact was…we’re trying to change a whole culture of how researchers think and work…if they can get concrete examples of how it’s worked in the past and how it’s been successful and how it’s enhanced the research, they may be more open to just having it proposed to them...” – P3.*

This was seen as particularly important when patient involvement was non-traditional (e.g. development of equal partnerships with researchers conducting trials).

### Mapping opportunities onto the orphan drug lifecycle

Opportunities for patients, families, and patient organizations mapped onto the orphan drug lifecycle are presented in Table D-1 (Additional file 4: Appendix D). The 12 themes were then mapped onto the lifecycle in Figures D-1 to D-3 (Additional file 4). These figures demonstrate that opportunities for patients, families, and patient organizations exist throughout the lifecycle. In fact, nine of the themes or categories of opportunities spanned the entire lifecycle. They also illustrate gaps in the literature that were subsequently filled through the webinar. Notably, almost none of the gaps related to opportunities for patient organizations.

## Discussion

Through the literature review, an inventory of existing and proposed opportunities for patients, families, and patient organizations at each stage of the orphan drug lifecycle was compiled. Further opportunities were identified by webinar participants, demonstrating the need for greater information-sharing of examples of patient and family involvement in Canada. Members of the rare disease community have been engaged in a variety of roles throughout the lifecycle of drugs for these diseases. However, there has not been a standardized approach to their engagement. As well, based on the literature the effects of this engagement on drug development, approval, or funding are unclear, since no evaluations have been done. Patients, families, and patient organizations have also proposed additional and increased roles for them in the lifecycle, suggesting that they feel current levels of involvement are inadequate. However, again, there is no framework proposed for a more consistent and meaningful engagement.

This situation does not appear to be unique to rare diseases. While no similar inventory of involvement opportunities for patients with common diseases could be found, it has been acknowledged that, in general, efforts need to be made to increase patient engagement in the research, development, and approval of new drugs [99, 100]. For all diseases, developing a drug that will improve patients’ lives requires a deep understanding of their medical condition, including their needs, preferences, and the trade-offs they are willing to make. These insights can be gained through improved patient involvement, leading to more relevant and impactful patient outcomes and make drug development faster, more efficient, and more productive. To this end, researchers have worked to develop frameworks for patient involvement in research [101–103] and the pharmaceutical industry has proposed the development of a “master framework” for integrated and systematic patient involvement [99]. However, there are differences between common diseases and rare diseases and it is reasonable to expect that the need for and best approach to patient involvement may differ between them. Given the nature of rare diseases (i.e. low prevalence; disease heterogeneity), clinical investigators often have less exposure to and understanding of the disease and its progression. As a result, there is a greater chance of failing to capture what is important to patients. While this inventory may have some relevant learnings for common diseases, it was compiled with a focus on rare diseases and the specific challenges associated with developing orphan drugs. With these challenges in mind, the National Center for Advancing Translational Sciences recently released their Toolkit for Patient-Focused Therapy Development. Launched in September 2017, the toolkit provides online resources to patient organizations intended to help them advance medical research on their disease (e.g. how to establish a patient registry; how to conduct post-market surveillance) [104].

This study has several limitations. The published papers, grey literature, and websites reviewed were limited to English only. Additionally, with the exception of the assessment of regulatory and reimbursement decision-making processes, the results were limited to papers describing opportunities for rare disease patients only. Therefore, some opportunities may have been missed. Finally, only one webinar was held, and it involved members of a patient liaison group within a research collaborative. Although actively engaged in Canada’s rare diseases community for many years, they may not have been familiar with all opportunities for involvement.

## Conclusion

There are a number of opportunities, both existing and proposed, for patients, their families, and the organizations representing them to become involved in the orphan drug lifecycle. However, based on the information found, it is not possible to determine which opportunities would be most effective at each stage of the lifecycle.

## Additional files

Additional file 1: Appendix A includes full details of the search terms and sources used in the literature search, the PRISMA diagram, and a detailed tabulation of the literature search results. (DOCX 297 kb)

Additional file 2: Appendix B contains the detailed tabulation of the review of websites of regulatory and reimbursement processes [105–131]. (DOCX 64 kb)

Additional file 3: Appendix C contains definitions of the themes identified. (DOCX 34 kb)

Additional file 4: Appendix D contains figures depicting the identified opportunities for patients, families, and patient organizations mapped onto the orphan drug lifecycle. (DOCX 147 kb)

## Abbreviations

CDR: Common Drug Review
EHC: European Haemophilia Consortium
GDP: Gross Domestic Product
MeSH: Medical Subject Headings
MLA: Member of the Legislative Assembly
MP: Member of Parliament
NORD: National Organization for Rare Disorders
OECD: Organization for Economic Co-operation and Development
PRISM: Promoting Rare-Disease Innovations through Sustainable Mechanisms
PRO: Patient Reported Outcome
PROM: Patient Reported Outcome Measure
