# Supplementary material for: Exploring patient and family involvement in the lifecycle of an orphan drug: a scoping review
Source: Orphanet J Rare Dis. 2017 Dec 22;12:188. doi: 10.1186/s13023-017-0738-6 (PMC5741909; doi:10.1186/s13023-017-0738-6)
Supplement: Supplementary file 1 — Appendix A includes full details of the search terms and sources used in the literature search, the PRISMA diagram, and a detailed tabulation of the literature search results. (DOCX 297 kb) [file 13023_2017_738_MOESM1_ESM.docx]

**Appendix A. Literature Search Strategy and Results.**

**Literature search strategy**

| **Database** | **Vendor** | **Date searched** | **Notes** | **Results** |
| --- | --- | --- | --- | --- |
| MEDLINE | Ovid | May 2017 |  | 2791 |
| PubMed | [www.pubmed.gov](http://www.pubmed.gov) | May 2017 |  | 1392 |
| PubMed | [www.pubmed.gov](file:///C:\Users\aldunn\Downloads\www.pubmed.gov) | May 2017 |  | 149 |
| Cochrane Library | John Wiley | May 2017 | Selected results from Cochrane systematic reviews, DARE, HTA & Methods only | 19 |
| Centre for Reviews & Dissemination (DARE, NHS EED, HTA) | <http://www.crd.york.ac.uk/crdweb/> | May 217 |  | 189 |
| EMBASE | Ovid | May 2017 |  | 269 |
| Web of Science | Thomson Reuters | 7 Mar 2014 |  | 668 |
| EconLit | EBSCOHost | 7 Mar 2014 |  | 323 |

**1. MEDLINE (Ovid; Including in-process & other non-indexed citations; searched March 2014 with monthly updates until May 2017)**

| 1 | exp Patient Preference/ | 2791 |
| --- | --- | --- |
| 2 | exp Patient Participation/ | 17460 |
| 3 | exp Consumer Participation/ | 31120 |
| 4 | exp Consumer Satisfaction/ | 76452 |
| 5 | exp Parents/ | 69741 |
| 6 | exp Caregivers/ | 20608 |
| 7 | exp Family/ | 229578 |
| 8 | exp Patients/ | 6468 |
| 9 | 5 or 6 or 7 or 8 | 301515 |
| 10 | (involv* or engag* or participat* or prefer* or outcome assess* or choice* or value*).ti. | 33481 |
| 11 | 9 and 10 | 7149 |
| 12 | exp Patient Outcome Assessment/ | 160 |
| 13 | patient* prefer*.ti,ab. | 8275 |
| 14 | patient participation.ti,ab. | 1309 |
| 15 | patient engagement.ti,ab. | 391 |
| 16 | patient involvement.ti,ab. | 1098 |
| 17 | patient value*.ti,ab. | 591 |
| 18 | patient choice*.ti,ab. | 1184 |
| 19 | family participation.ti,ab. | 301 |
| 20 | family preference*.ti,ab. | 164 |
| 21 | caregiver participation.ti,ab. | 39 |
| 22 | caregiver preference*.ti,ab. | 52 |
| 23 | parent* participat*.ti,ab. | 1021 |
| 24 | parent* prefer*.ti,ab. | 568 |
| 25 | patient reported outcome*.ti,ab. | 3365 |
| 26 | ((pro or pros or prom or proms) and outcome*).ti. | 198 |
| 27 | 1 or 2 or 3 or 4 or 11 or 12 or 13 or 14 or 15 or 16 or 17 or 18 or 19 or 20 or 21 or 22 or 23 or 24 or 25 or 26 | 12323 |
| 28 | exp "diffusion of innovation"/ | 1692 |
| 29 | exp biomedical technology/ | 7165 |
| 30 | exp technology assessment, biomedical/ | 9191 |
| 31 | exp orphan drug production/ | 814 |
| 32 | exp rare diseases/ | 4681 |
| 33 | exp health priorities/ | 8694 |
| 34 | lifecycle of technolog*.ti,ab. | 7 |
| 35 | technolog* lifecycle.ti,ab. | 6 |
| 36 | orphan drug development.ti,ab. | 65 |
| 37 | rare disease*.ti,ab. | 12429 |
| 38 | rare condition*.ti,ab. | 10701 |
| 39 | rationing.ti,ab. | 2341 |
| 40 | priorit*.ti. | 9964 |
| 41 | health technolog*.ti,ab. | 3048 |
| 42 | health care technolog*.ti,ab. | 491 |
| 43 | healthcare technolog*.ti,ab. | 314 |
| 44 | medical technolog*.ti,ab. | 4641 |
| 45 | new treatment*.ti. | 3682 |
| 46 | new therap*.ti. | 5239 |
| 47 | 28 or 29 or 30 or 31 or 32 or 33 or 34 or 35 or 36 or 37 or 38 or 39 or 40 or 41 or 42 or 43 or 44 or 45 or 46 | 98721 |
| 48 | 27 and 47 | 2150 |
| **49** | **limit 48 to yr="2000 -Current"** | **3222** |

**2a. PubMed (**[**www.pubmed.gov**](http://www.pubmed.gov)**;** searched March 2014 with monthly update until May 2017)

| #55 | Search #32 AND #53 Filters: Publication date from 2000/01/01 | 1392 |
| --- | --- | --- |
| #54 | Search #32 AND #53 | 1791 |
| #53 | Search #33 OR #34 OR #35 OR #36 OR #37 OR #38 OR #39 OR #40 OR #41 OR #42 OR #43 OR #44 OR #45 OR #46 OR #47 OR #48 OR #49 OR #50 OR #51 OR #52 | 73495 |
| #52 | Search innovation[ti] | 5093 |
| #51 | Search "new therapy"[ti] OR "new therapies"[ti] | 1381 |
| #50 | Search "new treatment*.ti. | 1434 |
| #49 | Search "medical technolog*" | 1661 |
| #48 | Search "healthcare technolog*" | 21 |
| #47 | Search "health care technolog*" | 42 |
| #46 | Search "health technolog*" | 104 |
| #45 | Search priorit*[ti] | 10171 |
| #44 | Search rationing[tiab] | 2349 |
| #43 | Search "rare conditions"[tiab] | 795 |
| #42 | Search "rare disease"[tiab] OR "rare diseases"[tiab] | 12359 |
| #41 | Search "orphan drug development" | 55 |
| #40 | Search "technology lifecycle*" | 7 |
| #39 | Search technolog*[ti] AND lifecycle[ti] | 5 |
| #38 | Search health priorities[mh] | 8639 |
| #37 | Search rare diseases[mh] | 4691 |
| #36 | Search orphan drug production[mh] | 788 |
| #35 | Search technology assessment, biomedical[mh] | 9074 |
| #34 | Search biomedical technology[mh] | 6993 |
| #33 | Search diffusion of innovation[mh] | 15047 |
| #32 | Search #1 OR #2 OR #3 OR #4 OR #12 OR #13 OR #14 OR #15 OR #16 OR #17 OR #18 OR #19 OR #20 OR #21 OR #22 OR #23 OR #24 OR #25 OR #26 OR #27 OR #28 OR #29 OR #30 OR #31 | 115816 |
| #31 | Search (pro[ti] OR pros[ti] OR prom[ti] OR proms[ti]) AND outcome*[ti] | 201 |
| #30 | Search "patient reported outcomes"[tiab] | 2408 |
| #29 | Search "parental preferences"[tiab] | 113 |
| #28 | Search "parents preferences"[tiab] | 6 |
| #27 | Search "parental participation"[tiab] | 209 |
| #26 | Search "parent participation"[tiab] | 144 |
| #25 | Search "parents participation"[tiab] | 7 |
| #24 | Search "caregiver preferences"[tiab] | 29 |
| #23 | Search "caregiver participation"[tiab] | 31 |
| #22 | Search "family preferences"[tiab] | 104 |
| #21 | Search "family participation"[tiab] | 487 |
| #20 | Search "patient choice"[tiab] | 1026 |
| #19 | Search "patients values"[tiab] | 71 |
| #18 | Search "patient values"[tiab] | 386 |
| #17 | Search "patient involvement"[tiab] | 1026 |
| #16 | Search "patient engagement"[tiab] | 361 |
| #15 | Search "patient participation"[tiab] | 1292 |
| #14 | Search "patient prefer*" OR "patients prefer*" | 597 |
| #13 | Search patient outcome assessment[mh] | 139 |
| #12 | Search #9 AND #10 | 6976 |
| #10 | Search involv*[ti] OR engage*[ti] OR participat*[ti] OR prefer*[ti] OR "outcome assessment"[ti] OR choice*[ti] OR value*[ti] | 340171 |
| #9 | Search #5 OR #6 OR #7 OR #8 | 299719 |
| #8 | Search patients[mh] | 63761 |
| #7 | Search family[mh] | 227898 |
| #6 | Search caregivers[mh] | 20417 |
| #5 | Search parents[mh] | 69249 |
| #4 | Search consumer satisfaction[mh] | 76044 |
| #3 | Search consumer participation[mh] | 30912 |
| #2 | Search patient participation[mh] | 17298 |
| #1 | Search patient preference[mh] | 2567 |

**2b. PubMed additional search terms (searched May2017)**

| #66 | Search #65 NOT #15 Filters: Publication date from 2000/01/01 | 149 |
| --- | --- | --- |
| #67 | Search #65 NOT #15 – DUPLICATES FROM ORIGINAL SEARCH REMOVED | 149 |
| #65 | Search #61 AND #63 Filters: Publication date from 2000/01/01 | 4072 |
| #64 | Search #61 AND #63 | 5271 |
| #63 | Search #19 OR #20 OR #21 OR #22 OR #23 OR #26 OR #28 OR #29 OR #31 OR #32 | 336564 |
| #61 | Search #16 OR #18 | 117634 |
| #32 | Search "clinical trial protocol*"[ti] – ADDITIONAL TERMS | 26 |
| #31 | Search "clinical trial design"[ti] | 369 |
| #29 | Search "regulatory process*"[ti] | 101 |
| #28 | Search "regulatory approval*"[ti] | 60 |
| #26 | Search pragmatic clinical trials as topic[mh] | 43 |
| #23 | Search drug discovery[mh] | 8139 |
| #22 | Search drug approval[mh] | 10534 |
| #21 | Search diagnostic test approval[mh] | 48 |
| #20 | Search device approval[mh] | 2176 |
| #19 | Search ((diffusion of innovation[mh]) OR (biomedical technology[mh]) OR (technology assessment, biomedical[mh]) OR (orphan drug production[mh]) OR (rare diseases[mh]) OR (health priorities[mh]) OR (technolog*[ti] AND lifecycle[ti]) OR ("technology lifecycle*") OR ("orphan drug development") OR ("rare disease"[tiab] OR "rare diseases"[tiab]) OR ("rare conditions"[tiab]) OR (rationing[tiab]) OR (priorit*[ti]) OR ("health technolog*") OR ("health care technolog*") OR ("healthcare technolog*") OR ("medical technolog*") OR ("new treatment"[ti] OR "new treatments"[ti]) OR ("new therapy"[ti] OR "new therapies"[ti]) OR (innovation[ti])) | 246144 |
| #18 | Search "patient oriented research"[ti] | 65 |
| #16 | Search ((patient preference[mh]) OR (patient participation[mh]) OR (consumer participation[mh]) OR (consumer satisfaction[mh]) OR (((parents[mh]) OR (caregivers[mh]) OR (family[mh]) OR (patients[mh])) AND (involv*[ti] OR engage*[ti] OR participat*[ti] OR prefer*[ti] OR "outcome assessment"[ti] OR choice*[ti] OR value*[ti])) OR (patient outcome assessment[mh]) OR ("patient prefer*" OR "patients prefer*") OR ("patient participation"[tiab]) OR ("patient engagement"[tiab]) OR ("patient involvement"[tiab]) OR ("patient values"[tiab]) OR ("patients values"[tiab]) OR ("patient choice"[tiab]) OR ("family participation"[tiab]) OR ("family preferences"[tiab]) OR ("caregiver participation"[tiab]) OR ("caregiver preferences"[tiab]) OR ("parents participation"[tiab]) OR ("parent participation"[tiab]) OR ("parental participation"[tiab]) OR ("parents preferences"[tiab]) OR ("parental preferences"[tiab]) OR ("patient reported outcomes"[tiab]) OR ((pro[ti] OR pros[ti] OR prom[ti] OR proms[ti]) AND outcome*[ti])) |  |
| #15 | Search ((patient preference[mh]) OR (patient participation[mh]) OR (consumer participation[mh]) OR (consumer satisfaction[mh]) OR (((parents[mh]) OR (caregivers[mh]) OR (family[mh]) OR (patients[mh])) AND (involv*[ti] OR engage*[ti] OR participat*[ti] OR prefer*[ti] OR "outcome assessment"[ti] OR choice*[ti] OR value*[ti])) OR (patient outcome assessment[mh]) OR ("patient prefer*" OR "patients prefer*") OR ("patient participation"[tiab]) OR ("patient engagement"[tiab]) OR ("patient involvement"[tiab]) OR ("patient values"[tiab]) OR ("patients values"[tiab]) OR ("patient choice"[tiab]) OR ("family participation"[tiab]) OR ("family preferences"[tiab]) OR ("caregiver participation"[tiab]) OR ("caregiver preferences"[tiab]) OR ("parents participation"[tiab]) OR ("parent participation"[tiab]) OR ("parental participation"[tiab]) OR ("parents preferences"[tiab]) OR ("parental preferences"[tiab]) OR ("patient reported outcomes"[tiab]) OR ((pro[ti] OR pros[ti] OR prom[ti] OR proms[ti]) AND outcome*[ti])) AND ((diffusion of innovation[mh]) OR (biomedical technology[mh]) OR (technology assessment, biomedical[mh]) OR (orphan drug production[mh]) OR (rare diseases[mh]) OR (health priorities[mh]) OR (technolog*[ti] AND lifecycle[ti]) OR ("technology lifecycle*") OR ("orphan drug development") OR ("rare disease"[tiab] OR "rare diseases"[tiab]) OR ("rare conditions"[tiab]) OR (rationing[tiab]) OR (priorit*[ti]) OR ("health technolog*") OR ("health care technolog*") OR ("healthcare technolog*") OR ("medical technolog*") OR (ew treatment*.ti.) OR ("new therapy"[ti] OR "new therapies"[ti]) OR (innovation[ti])) – ORIGINAL SEARCH STRATEGY | 5116 |

**3. The Cochrane Library (searched May 2017)**

* scanned & selected only results from Cochrane review, Other reviews, Methods, and HTA

#1 MeSH descriptor: [Patient Preference] explode all trees 631

#2 MeSH descriptor: [Patient Participation] explode all trees 916

#3 MeSH descriptor: [Consumer Participation] explode all trees 1408

#4 MeSH descriptor: [Consumer Satisfaction] explode all trees 10032

#5 MeSH descriptor: [Patient Outcome Assessment] explode all trees 321

#6 "patient participation" 1977

#7 "patient engagement" 403

#8 "patient involvement" 750

#9 "patient preference*" 2465

#10 "family participation" 34

#11 "family preference*" 22

#12 "caregiver participation" 12

#13 "caregiver preference*" 19

#14 "parental participation" 39

#15 "parental preference*" 41

#16 "patient reported outcomes" 731

#17 "patient values" 62

#18 "patients values" 74

#19 "patient choice*" 194

#20 "patient oriented research" 61

#21 #1 or #2 or #3 or #4 or #5 or #6 or #7 or #8 or #9 or #10 or #11 or #12 or #13 or #14 or #15 or #16 or #17 or #18 or #19 or #20 14087

#22 MeSH descriptor: [Diffusion of Innovation] explode all trees 191

#23 MeSH descriptor: [Treatment Outcome] explode all trees 88431

#24 MeSH descriptor: [Biomedical Technology] explode all trees 94

#25 MeSH descriptor: [Health Care Rationing] explode all trees 107

#26 MeSH descriptor: [Technology Assessment, Biomedical] explode all trees 801

#27 MeSH descriptor: [Orphan Drug Production] explode all trees 10

#28 MeSH descriptor: [Rare Diseases] explode all trees 21

#29 MeSH descriptor: [Health Priorities] explode all trees 62

#30 lifecycle and technolog* 6

#31 "orphan drug*" 35

#32 "rare diseases" 141

#33 rationing 374

#34 innovation 1294

#35 #22 or #23 or #24 or #25 or #26 or #27 or #28 or #29 or #30 or #31 or #32 or #33 or #34 90534

#36 #21 and #35 19

**DARE, NHS EED, HTA databases)**

**4. UK Centre for Reviews and Dissemination (DARE, NHS EED, HTA databases) (**[**www.crd.york.ac.uk/crdweb**](http://www.crd.york.ac.uk/crdweb)**; searched May 2017)**

1 (patient*):TI 8793

2 (participation):TI OR (involv*):TI OR (preference*):TI 321

3 (engagement):TI OR (engaging):TI OR (choice*):TI 109

4 ("reported outcome*"):TI OR ("patient value*"):TI OR (PROM*):TI 485

5 (parent*):TI OR (caregiver*):TI OR (family):TI 560

6 #1 OR #5 9221

7 #2 OR #3 OR #4 851

8 #6 AND #7 191

9 * FROM 2000 TO 2014 60174

10 #8 AND #9 189

**5. EMBASE (Ovid; 1974 to 2017 May)**

| 1 | exp patient preference/ | 4798 |
| --- | --- | --- |
| 2 | exp patient participation/ | 17337 |
| 3 | exp parent/ | 153400 |
| 4 | exp caregiver/ | 3941 |
| 5 | exp family/ | 336864 |
| 6 | 3 or 4 or 5 | 364512 |
| 7 | (involv* or engag* or participat* or prefer* or outcome assess* or choice* or value*).ti. | 410678 |
| 8 | 6 and 7 | 7864 |
| 9 | patient outcome assessment.mp. | 312 |
| 10 | patient engagement.mp. | 490 |
| 11 | patient involvement.mp. | 1521 |
| 12 | patient reported outcome*.mp. | 6501 |
| 13 | PROMS.ti,ab. | 306 |
| 14 | patient value*.ti. | 72 |
| 15 | patient choice*.ti. | 394 |
| 16 | patient oriented research.mp. | 209 |
| 17 | diffusion of innovation.mp. | 377 |
| 18 | exp medical technology/ | 31497 |
| 19 | health care rationing.mp. | 322 |
| 20 | exp biomedical technology assessment/ or technology assessment.mp. | 15163 |
| 21 | orphan drug production.mp. | 16 |
| 22 | exp rare disease/ | 18019 |
| 23 | exp orphan drug/ | 1511 |
| 24 | health priorities.mp. | 1082 |
| 25 | lifecycle of technology.mp. | 6 |
| 26 | technology lifecycle.mp. | 7 |
| 27 | orphan drug development.mp. | 59 |
| 28 | innovation.ti. | 6588 |
| 29 | 17 or 18 or 19 or 20 or 21 or 22 or 23 or 24 or 25 or 26 or 27 or 28 | 72416 |
| 30 | 1 or 2 or 8 or 9 or 10 or 11 or 12 or 13 or 14 or 15 or 16 | 37841 |
| 31 | 29 and 30 | 341 |
| 32 | limit 31 to yr="2000 -Current" | 280 |

**6. Web of Science (Thomson Reuters; searched 7 Mar 2014)**

| # 3 | [668](http://apps.webofknowledge.com/summary.do?product=UA&doc=1&qid=3&SID=3Di1dvssPbT8JxD1wkf&search_mode=CombineSearches) | #2 AND #1  Timespan=2000-2017  Search language=English |
| --- | --- | --- |
| # 2 | [Approximately 784,692](http://apps.webofknowledge.com/summary.do?product=UA&doc=1&qid=2&SID=3Di1dvssPbT8JxD1wkf&search_mode=GeneralSearch) | TOPIC: (diffusion) OR TOPIC: ("biomedical technolog*") OR TOPIC: ("technology assessment*") OR TOPIC: ("health care rationing") OR TOPIC:("orphan drug*") OR TOPIC: ("rare disease*") OR TOPIC: ("lifecycle of technolog*") OR TOPIC: ("technology lifecycle") OR TOPIC: (rationing) ORTOPIC: (innovation)  Timespan=2000-2014  Search language=English |
| # 1 | [Approximately 44,034](http://apps.webofknowledge.com/summary.do?product=UA&doc=1&qid=1&SID=3Di1dvssPbT8JxD1wkf&search_mode=GeneralSearch) | TOPIC: ("patient preference*") OR TOPIC: ("patient participation") OR TOPIC: ("patient engagement") OR TOPIC: ("patient involvement") OR TOPIC:("family participation") OR TOPIC: ("family preferences") OR TOPIC: ("caregiver participation") OR TOPIC: ("caregiver preference*") OR TOPIC:("parental participation") OR TOPIC: ("parental preference*") OR TOPIC: ("parent* preference*") OR TOPIC: ("patient outcome assessment") OR TOPIC:("patient oriented research") OR TOPIC: ("patient* value*") OR TOPIC: ("patient* choice*") OR TOPIC: ("patient reported outcome*")  Timespan=2000-2014  Search language=English |

**7. EconLit (EBSCOHost; searched 7 May 2017)**

| S3 | (diffusion of innovation OR biomedical technolog* OR health care rationing OR orphan drug* OR rare disease* OR lifecycle of technolog* OR innovation) AND (S1 AND S2) | (331) |
| --- | --- | --- |
| S2 | diffusion of innovation OR biomedical technolog* OR health care rationing OR orphan drug* OR rare disease* OR lifecycle of technolog* OR innovation | (52,061) |
| S1 | patient preference* OR patient participation OR patient outcome assessment OR patient oriented research OR patient choice* OR patient value* OR ( (parent* OR caregiver OR family) AND (preference* OR participation OR value* OR choice*) ) | (13,562) |

**Grey literature searches**

| Source | URL | Date | Search terms | Results |
| --- | --- | --- | --- | --- |
| ProQuest Dissertations & Theses | Subscription required | May 2017 | ti(patient* OR caregiver* OR family OR parent*) AND ti((involvement OR participation OR engagement)) AND ti((technology OR technologies OR treatment* OR therapy OR therapies OR lifecycle)) | 64 (none relevant) |
| Grey Literature Collection (New York Academy of Medicine) | [www.nyam.org/library/](http://www.nyam.org/library/) | May 2017 | “(patient or family or caregiver or parent) AND (engagement OR participation OR involvement) AND (lifecycle OR technology OR treatment OR therapy)” | 92 (4 possibly relevant) |
| KU-UC (Reseau de recherché en santé des population du Quebec) | [www.santepop.qc.ca/en/recherchemotscles.html?2](http://www.santepop.qc.ca/en/recherchemotscles.html?2) | May 2017 | scanned all reports under keyword heading Technology Assessment  searched keywords: “patient participation”  “patient engagement” | 214  1  4 |
| UK NHS Evidence | [www.evidence.nhs.uk/](https://www.evidence.nhs.uk/) | May 2017 | ("patient engagement" OR "patient involvement" OR "patient participation") AND (technology OR technologies OR lifecycle) | 3367 (only scanned through first 500 hits; 11 possibly relevant) |
| The Patient – Patient-Centered Outcomes Research | <http://link.springer.com/journal/40271> | May 2017 | Scanned tables of contents for all issues (2008-2014) | 13 |
| US Food and Drug Administration Patient-Focused Drug Development | [www.fda.gov/ForIndustry/UserFees/PrescriptionDrugUserFee/ucm347317.htm](http://www.fda.gov/ForIndustry/UserFees/PrescriptionDrugUserFee/ucm347317.htm) | May 2017 | Scanned web page and links to meeting documents & The Voice of the Patient reports | 4 |
| European Patients Forum | [www.eu-patient.eu](http://www.eu-patient.eu) | May 2017 | Scanned web page | 9 |
| European Patients’ Academy on Therapeutic Innovation | [www.patientsacademy.eu](http://www.patientsacademy.eu) | May 2017 | Scanned web page | 1 |
| Patient-Centered Outcomes Research Institute | [www.pcori.org](http://www.pcori.org) | May 2017 | Scanned web page | 3 |
| James Lind Alliance | [www.lindalliance.org/](http://www.lindalliance.org/) | May 2017 | Scanned web page | 4 |
| HTAi Patient & Citizen Involvement | [www.htai.org](http://www.htai.org) | May 2017 | Scanned web page of special interest group section | 4 |
| Google.ca | [www.google.ca](http://www.google.ca) | May 2017 | “patient focussed drug development” / “patient focused drug development”  ("patient engagement" OR "patient involvement" OR "patient participation") AND (technology OR technologies OR lifecycle)  "patient involvement" "technology life cycle" | 4 / 301,000 (only scanned first 100 hits)  288,000 (8 possibly relevant; only scanned first 200 hits – mainly on patient engagement & information technologies)  34 |
| Google Scholar | [www.googlescholar.com](http://www.googlescholar.com) | 21 Mar 2014 | “patient involvement in health technologies development and lifecycle”  (limited 2000-2014)  "patient involvement" development "orphan drugs" | 15  296 (33 possibly relevant) |

**Figure A-1. PRISMA diagram of literature search results & study selection for review of opportunities for patient, family, and patient organization involvement in the orphan drug lifecycle.**

|  | Records identified through database searching (n = 5800) | | |  | Additional records identified through other sources (n = 24) | | | | |  |
| --- | --- | --- | --- | --- | --- | --- | --- | --- | --- | --- |
|  |  | |  |  |  | |  | | |  |
|  | | |  | | | |  | | | |
|  | | | Records after duplicates removed (n = 3582) | | | |  | | | |
|  | | |  | | | |  | | | |
| Excluded papers related to: commentaries, editorials, or perspective pieces (14); abstract only (13); patient, family, or patient organization input not discussed (9); information was duplicated in another study (7); focused on a common disease (5); and insufficient information available to describe the opportunity for involvement (3). | | | Titles and abstracts reviewed  (n = 3,582) | | | |  | Records excluded (n = 3,424) | | |
|  | | |  | | | |  |  | | |
|  | | | Full-text articles assessed for eligibility (n = 158) | | | |  | | | |
|  | | |  | | |  | | | | |
|  | | Studies included in qualitative synthesis (n = 84) | |  | Full-text articles excluded, with reasons (n = 74) | | | |  | |

| **Table A-1. Opportunities for patient, family, and patient organization involvement identified in the literature review.** | | | | | |
| --- | --- | --- | --- | --- | --- |
| **Primary author (year)** | **Country** | **Disease area** | **Participants^‡^** | **Role** | **Impact or outcome** |
| ***Research^†^*** | | | | | |
| *Participation as a research subject* | | | | | |
| Syed  (2015)  [133] | Denmark and United Kingdom | Rare disease in general | Patients, families, and patient organizations | • Patients, families, and patient organizations participated in a study using a modified 3-round Delphi technique (questionnaire, face-to-face meetings, validation and sign-off) to assess patients’ experiences at selected Centres of Expertise (CoEs), identify policy areas with gaps for discussions on the current services, and develop policy recommendations for rare disease centres of expertise in order to improve standards and quality of care | • Policy recommendations were developed in areas previously recognized as having gaps |
| Bedgood  (2007)  [132] | International  (United States, Canada, United Kingdom, Australia, Colombia, Germany, Israel, New Zealand, and the Philippines) | Achalasia | Patients | • Patients completed an online survey collecting data on demographics, symptom onset, presenting symptoms, and success of current treatments | • 83 surveys were completed globally  • The authors state that Internet-based surveys may be useful in the accumulation of information on uncommon diseases, which are generally difficult to study due to the limited numbers of patients at single centers |
| Henrard  (2015)  [105] | Belgium | Haemophilia | Patients | • Patients completed a questionnaire evaluating what motivates people with haemophilia to participate in clinical research and to identify factors that might influence their willingness to participate | • 62 questionnaires were completed  • The rate of willingness to participate was significantly lower in patient who reported having no knowledge of clinical trial modalities |
| Coathup  (2016)  [134] | Japan | Myotonic dystrophy | Patients | • Patients completed a questionnaire regarding patients’ views and attitudes to using digital tools in patient registries and engagement with medical research in Japan | • The majority of patients were not receiving the information they want from clinicians (e.g. research findings, opportunities to participate in trials) and are willing to engage with digital technologies to receive relevant medical information, They are also interested in having control over when and how they receive this information, and being informed of how their data is used and shared |
| Serrano-Aguilar  (2009)  [93] | Spain | Degenerative ataxias (DAs) | Patients | • Patient input was incorporated into the completion of a systematic review of the literature on the effectiveness of treatment for DAs by having patients complete 3 questionnaires as part of the Delphi Method for consultation:  (1) an open questionnaire on the treatments used for DA and patients’ most relevant self-perceived health problems associated with their disease,  (2) a prioritization of the health problems identified in questionnaire 1, and  (3) an opportunity to revise earlier answers based on the overall, ranked results of questionnaire 2 | • The authors indicate that patient participation was effective in enhancing the design and conduct of the systematic review  •Patients were able to identify relevant research needs and highlight variations in values and access to different treatments across regions/countries  • Some health problems and outcome measures identified by the patients were not found in any of the studies included in the review, indicating an evidence gap that DA researchers and policy makers should consider in the design of future projects |
| Morel  (2016)  [135] | United Kingdom | Rare diseases in general | Patients and families | • Patients and families across 52 rare diseases participated in a mix-methods survey to explore what they consider of value when choosing between hypothetical therapeutic options and to quantify both their benefit-risk preferences and the influence of disease context  • Discrete-choice experience comparing hypothetical treatment profiles of benefits and risks were used to measure preferences across 7 attributes related to health outcomes, safety, and process of care.  • Disease context was describe through bespoke questions on current disease management and the joint use of the 12-item WHODAS 2.0 questionnaire and 2 Likert scales capturing self- and proxy assessed disease-induced threat to life and impairment  • Qualitative insights on patient and family definitions of value and risk were also collected | • Respondents attributed most importance to drug response, risk of serious side effects, and the ability to conduct usual activities while on treatment. Attributes related to treatment modalities were the least important. There was a willingness to accept risks in hopes of finding some benefit (e.g. higher chance of drug response or greater health improvement potential). Increasing disease severity, impairment or disability, and the lack of effective therapeutic options significantly raised the willingness to gain benefit through risk.  • The study provides a more detailed understanding of the relationship between disease context, treatment attributes, and the degree of risk respondents are willing to take to gain a specific degree of benefits.  • It is suggested that researchers of novel therapeutics for rare diseases invest in preference elicitation studies to generate rigorous patient evidence and specific regulatory guidance should be issued to acknowledge their importance and their use in marketing authorisations. |
| Swinburn  (2012)  [94] | United Kingdom | Advanced neuroendocrine tumors (NETs) | Patients | • Patients participated in exploratory interviews in which they described their experiences living with NETs and undergoing therapy  • Patients also completed the EQ-5D assessment exercise, rating their health on 5 different dimensions  • These health states were collected to be used to elicit utility values for the health states of patients undergoing treatment for NETs | • Patient interviews, along with the results of a literature review and interviews with clinical experts, were used to develop 10 vignettes describing the health states of advanced NET patients  • A time trade-off methodology was then used to have members of the UK public value the health states  • The authors suggest that the health utility values resulting from this study could inform cost-effectiveness assessments for advanced NET therapies |
| Carroll  (2012)  [136] | United States | Pulmonary arterial hypertension (PAH) | Patients | • Patients participated in semi-structured interviews discussing the factors that influence their decision to enroll in randomized controlled trials (RCTs) | • 24 factors that influence the RCT enrollment decisions of patients with PAH were identified  • The results indicate that by minimizing time demands of participating, providing financial remuneration, and allowing participants to continue current therapies may enhance enrollment to trials in similar disease areas  •A need to ensure patients understand the distinction between research and clinical care was also found, as many patients demonstrated an increased willingness to participate based on expectations of perceived personal benefit |
| De Blieck  (2013)  [35] | United States | Juvenile neuronal ceroid lipofuscinosis (JNCL or Batten Disease) | Patients | • As part of the development and validation of a disease-specific clinical outcome measure for JNCL, patients were assessed by researchers from the University of Rochester’s Batten Centre (URBC) using their Unified Batten Disease Rating Scale (UBDRS), which involves a physical examination and an evaluation of cognitive and behavioural symptoms using well-established neuropsychological measures | • 120 subjects were evaluated with the scale at least once and 95 children provided quantifiable measures of neurobehavioral function over time  • Disease burden and rate of progression were evaluated and quantified using the UBDRS data in 82 subjects with genetically confirmed JNCL, representing the largest cohort of Batten-disease patients reported to date using a disease-specific rating scale  • The collection of neuropsychological data permitted the objective assessment of change in neurobehavioral function over time and was also used to successfully cross-validate the UBDRS  • Telemedicine for remote UBDRS assessment was also successfully piloted |
| De Blieck  (2013)  [35] | United States | JNCL | Patients | • As part of the URBC’s efforts to validate a non-invasive, child-friendly method of obtaining cells for genotyping, patients participating in the URBC’s UBDRS study provided buccal epithelial cell samples to the URBC for genetic diagnosis | • Patients without a prior genetic diagnosis were successfully diagnosed, with the majority of samples being collected from buccal specimens  • Several novel mutations were identified through this process |
| Consolaro  (2016)  [106] | Not specified | JIA | Patients | • Patients participated in studies assessing the use of smartphones for collecting data on pain and disease symptoms | • The study found that smartphones improve the quality of self-reported data and make it easier to obtain repeated measures over a short time frame |
| Khodyakov  (April 2017)  [107]  *Proposed* | United States | DMD | Patients and families | • Patients and families will participate in interviews to help design a new method for engaging them in guideline development. They will be providing feedback on how the RAND/UCLA Appropriateness Method (RAM; the gold standard approach for conducting clinical expert panels) should be modified for the purposes of patient engagement and what rating criteria should patients and families use to provide input during the process of guideline development.  • Once the method is piloted, it will be tested with two concurrently run patient/family panels that will rate patient-centeredness of a subset of DMD care management recommendations already deem clinically appropriate and necessary | • Not applicable |
| Johnson  (2016)  [139] | Europe | Dysmelia | Families | • Parents completed online surveys regarding satisfaction with service, occurrence of signposting (where contact details for organizations able to provide further information/support are provided) and preferences when dysmelia is detected in maternity services | • Participants were less than satisfied with the service they received and only 27% were offered signposting formation.  • 91% of parents would have wanted signposting information and 67% would have wanted access to a support group. |
| Bendixen  (2016)  [98] | United States | Duchenne Muscular Dystrophy (DMD) | Families | • Parents of patients with DMD participated in focus groups to identify knowledge, attitudes and perceptions of participation in clinical research in DMD | • Parents provided the researchers with numerous thoughtful and novel ideas about ways to engage families in research in DMD |
| O’Mahony  (2014)  [96] | Europe | Hemophilia | Patient organizations | • 19 of the 43 patient organizations affiliated with the European Haemophilia Consortium (EHC) completed an EHC survey that assessed the standard of care for people with haemophilia in all European Countries | • Based on the results of the survey, the EHC have produced information packs for every National Member Organization affiliated with the countries that responded  •The information provided shows exactly where the country ranks in terms of availability of care, compared with the rest of Europe and includes suggested uses for the data  • The data collected were key in influencing the European Directorate for Quality of Medicines & HealthCare (EDQM) recommendation that in order to optimise the organization of haemophilia care nationally, a formal body should be established in each country including a national haemophilia patient organization. This was crucial for securing an agreement between the Ministry of Health in Romania, the EHC and the Romanian Haemophilia Association to establish a formal national haemophilia committee in Romania in 2013. [117] |
| *Research priority-setting* | | | | | |
| Nierse  (2013)  [137] | Netherlands | Neuromuscular diseases (NMDs) | Patients | • Patients participated in interviews and focus groups, and completed a questionnaire to identify their research priorities | • This study demonstrates that patients can formulate relevant research questions  • Patients highly valued research on cure and prevention of the occurrence of symptoms  • Patients and professionals identify a need to balance fundamental research and research on preventing and treating symptoms as well as research on slowing down disease progression  • This patient-driven agenda validates the wide scope of rehabilitation described in the International Classification of Functioning, Disability, and Health (ICF) model, and also identifies the need for more interdisciplinary research |
| Van Merode  (2016)  [138] | Netherlands | Waldenstrom’s disease | Patients | • Patients participated in individual interviews and focus groups to identify patients’ needs in the context of research priorities on important topics for quality of care improvement. | • Topics identified include improved information on all aspects of disease and treatment, involving patients in decision making, organization of care, and the burden of neuropathy. |
| Davila-Seijo  (2013)  [18] | Spain | Dystrophic Epidermolysis Bullosa | Patients | • Patients participated in three phases of a priority-setting exercise (Priority Setting Partnership, or PSP, method): a consultation survey, ranking exercises, and small workshops | • 6 uncertainties were identified that patients, families, and health care professionals feel are priority areas for future research  • This study also demonstrated that the PSP method can be utilized in a rare diseases setting |
| Khodyakov  (May 2017)  [107] | United States | Kawasaki disease | Patients and families | • Patients and families took part in online modified-Delphi panels, participating in 2 rating rounds with a statistical feedback and online discussion round in between | • Online approaches to engaging stakeholders, including patients and families, are promising and efficient in addition to in-person meetings |
| Woodward  (2016)  [156] | International | Atypical Haemolytic Uraemic Syndrome (aHUS) | Patient organizations | • The aHUS Alliance included a call for research ideas at their third meeting. The session was facilitated by the Alliance’s representative on the Global aHUS Registry scientific advisory board (SAB)  • A preliminary report was made available to all affiliates online, allowing for additional suggestions to be made.  • The final research suggestions were provided to the Chair of the aHUS registry and reported to the SAB by the Alliance representative. | • The work plan for the aHUS Registry in 2016 featured a number of topics proposed by the Alliance. |
| Nierse  (2013)  [137] | Netherlands | NMDs | Patient organizations | • Active members of the Dutch Patient Association for NMD (VSN) participated in expert meetings to complement and validate the priority topics identified by patients during interviews and focus groups  • VSN members also participated in a dialogue meeting with researchers and clinicians to develop the shared agenda | • The dialogue meeting provided a learning experience for all members involved and, as a result, the professionals acknowledged diverse areas for research that mattered to patients  • Through the sharing of perspectives in the final dialogue meeting, a shared research agenda was developed  • A research agenda was developed |
| Nierse  (2013)  [137] | Netherlands | NMDs | Patient organizations | • A staff member of VSN was a member of the team developing the agenda | • A research agenda was developed |
| Caron-Flinterman  (2005)  [42] | Germany | Retinitis Pigmentosa | Patient organizations | • Pro Retina, a German Retinitis Pigmentosa patient group, formulated research priorities for the scientific community | Not specified |
| *Initiation of research* | | | | | |
| Ferguson  (2002)  [19] | United States | Chromosome 18 deletion | Patients and families | • A mother of a patient noticed improvement in her child’s symptoms after taking human growth hormone and developed a theory to explain her observations based on available scientific literature | • Researchers tested the theory, finding it to be correct |
| Mai  (2012)  [57] | United States | Li-Fraumeni syndrome (LFS) | Patients and families | • Patients participated in a workshop around clinical research on LFS at the National Institutes of Health with scientists and physicians, sharing their experiences and their goals | • Patients and families identified 6 priorities for clinical research  • A new advocacy group was established to facilitate effective communication between LFS families and the clinical and scientific members of the research consortium |
| Caron-Flinterman  (2005)  [42] | Netherlands | Addison’s disease; Cushing’s disease | Patient organizations | • The Dutch Addison and Cushing Society (NVACP) requested an independent research facility to complete a study on improved drug administration methods for the treatment of Addison’s disease | • The NVACP-requested study eventually led to the establishment of a research project on delayed release tablets |
| Panofsky  (2011)  [145] | United States | Rare diseases in general | Patient organizations | • Patient organizations develop close relationships with, and between, scientists (i.e., “sociability”) to influence the research process  • Additional mechanisms utilized include resources; collective mobilization; timing; lay expertise; and organization controls  • Note: according to the author, without sociability, these additional mechanisms are insufficient for influencing research | • “Sociability” provides benefits for patient organizations and can help scientists develop stronger research networks and improved productivity |
| Patient Partner  [20] | Not specified | Rare diseases in general | Patient organizations | • Patient organizations promote the running of a trial by gathering researchers; demonstrating interest in new treatments; and bringing together research teams | Not specified |
| Landy  (2012)  [55] | Not specified | Rare diseases in general | Patient organizations | • Patient organizations have written funding proposals to conduct their own research | Not specified |
| *Assist in the conduct of research* | | | | | |
| Molster  (2012)  [95]  *Proposed* | Australia | Rare diseases in general | Patients and families | • Participants in the Australian Rare Diseases Symposium suggested that patients and families should be directly involved in decisions about research on rare diseases | Not applicable |
| Doyle  (2015)  [140] | United States | Cystinosis | Patients and families | • Community members provided input into the development of interview guides for focus groups and interviews as part of a qualitative study exploring the experiences of patients interacting in a disease community | • Three core questions were developed  • Six focus groups and 17 semi-structured interviews were conducted with adult patients and the parents of young patients |
| Johnson  (2016)  [139] | Europe | Dysmelia | Families | • Surveys regarding parents’ experiences in maternity services after detection of Dysmelia were developed based on clinical need as identified by parents of children with dysmelia who were associated with the European Dysmelia Reference Information Centre | • Participants were less than satisfied with the service they received and only 27% were offered signposting formation.  • 91% of parents would have wanted signposting information and 67% would have wanted access to a support group. |
| Montano  (2007)  [30] | International  (Australia, Austria, Brazil, Canada, Chile, Colombia, Finland, France, Germany, Indonesia, Italy, Japan, Morocco, New Zealand, Poland, Puerto Rico, Saudi Arabia, Spain, Switzerland, Turkey, United Kingdom, United States)  Note: study conducted out of the United States | MPS IVA | Patient organizations | • Survey questions given to individuals registered in an MPS IVA registry were first reviewed and discussed by a panel of board members from the International Morquio Organization (IMO), a patient organization for those with MPS | • The information collected will help to facilitate clinical trials on ERTs, as information on the natural history, rate of progression, and distribution of symptoms in untreated patients is necessary for developing clinical endpoints and judging therapeutic effects  • The authors suggest an annual survey will allow for the collection of more data on the management of patients, efficacy of treatment, and endpoints of clinical trials |
| De Moerloose  (2015)  [146] | International  (Ireland, UK, France, Sweden, Canada, the Netherlands, and Poland) | Haemophilia | Patient organizations | • National haemophilia patient organizations participated in a survey to assess the long-term effects of prophylaxis and the continuing benefit of treatment into adulthood. Each organization randomly selected severe haemophilia patients to be included in the survey, | •The findings demonstrated that prophylaxis started at an early age and continued into adulthood resulted in less bleeding, less damage to joints, and less time missed at work. |
| Nierse  (2013)  [137] | Netherlands | NMDs | Patient organizations | • The VSN helped to pilot and distribute the questionnaire, and also recruited patients for interviews and focus groups, which were used to develop the research agenda | • A research agenda was developed |
| Serrano-Aguilar  (2009)  [93] | Spain | DAs | Patient organizations | • Patient organization leaders helped to enroll research participants via email | • The authors indicate that patient participation was effective in enhancing the design and conduct of the systematic review  •Patients were able to identify relevant research needs and highlight variations in values and access to different treatments across regions/countries  • Some health problems and outcome measures identified by the patients were not found in any of the studies included in the review, indicating an evidence gap that DA researchers and policy makers should consider in the design of future projects |
| Ferguson  (2002)  [19] | United States | Pseudoxanthoma elasticum (PXE) | Patient organizations | • PXE International, an organization started by the parents of patients, collaborates with medical researchers, advising on symptoms of PXE and consulting on research strategies | Not specified |
| Landy  (2012)  [55] | Not specified | Rare diseases in general | Patient organizations | • Patient organizations provide researchers with letters of support | Not specified |
| Landy  (2012)  [55] | Not specified | Rare diseases in general | Patient organizations | • Patient organizations provide advice on the design of research projects | Not specified |
| Landy  (2012)  [55] | Not specified | Rare diseases in general | Patient organizations | • Patient organizations participate in data collection and analysis | Not specified |
| Landy  (2012)  [55] | Not specified | Rare diseases in general | Patient organizations | • Patient organizations help recruit research subjects | Not specified |
| *Partner in or lead the conduct of research* | | | | | |
| Frost  (2008)  [53] | International | Amyotrophic lateral sclerosis (ALS) | Patients | • Patients established a website providing a rationale for lithium treatment for ALS (based on the results of a study done in Italy) and a spreadsheet for users to report on: functional state pre- & post-initiation of lithium therapy; lithium dosage; lithium blood levels; and annotations to record side-effects, benefits, and to specify other treatments  • Subsequently, Patients Like Me, in collaboration with the patients who began the study, started tracking patient experiences with lithium treatment | • The number of patients taking lithium treatment in the Patients Like Me community increased from 1 to 116 with 4 months of a post referencing a study that showed a potential benefit of its use  • Since publication of the paper, patients having tried lithium increased to over 250, with 125 having completed a side-effects survey  • This article does not describe the results of the patient-driven study |
| Ferguson  (2002)  [19] | United States | Chromosome 18 deletion | Patients and families | • The mother of a patient, as a graduate student, developed a treatment for children with chromosome 18 deletion | • This treatment is now the first effective therapy being offered |
| Tosi  (2015)  [141] | United States | Osteogenesis imperfecta (OI) | Patients and patient organizations | • The Osteogenesis Imperfecta Foundation established the Adult Natural History Initiative (OI-ANHI), which tested the feasibility of an online health survey to 1) define health care concerns and perceptions of adults with OI, 2) identify health-care related issues that may have been previously missed or under-valued by adults with OI and their medical providers, and 3) compare QO responses by adults with OI with those of benchmark populations without OI  • The OI-ANHI is led by a committee that includes patients and patient organization representatives  • Surveys were developed based on focus groups with patients | • The OI-ANHI survey supports the use of an internet-based strategy for successful patient-centered outcomes research in rare disease populations  • Adults with OI report lower general health status but are more similar to the general population than might be expected. |
| Scarpa  (2011)  [45] | Europe | MPS II | Patient organizations | • Patient organizations gather information regarding the effect that treatments have on quality of life by having members complete surveys | Not specified |
| Rabeharisoa  (2003)  [56] | France | NMDs | Patient organizations | • The French Muscular Dystrophy Organization (AFM) created a scientific council, but generally establishes its own research policy through the board of governors  • e.g., the establishment of Genethon, a project that produces genes maps and provides customized sequencing services to outside research teams, was done with little involvement and support from the council | • Almost half of the AFM budget is spent on research |
| Rabeharisoa  (2003)  [56] | France | NMDs | Patient organizations | • Patients and their families actively partner with specialists in the production of knowledge and the care and treatment of their disease  • Patients are developing the tools necessary to formalize their “experiential knowledge” so that professionals, etc. will recognize its importance  • e.g., a special interest group on spinal muscular atrophy (SMA) within the AFM collected information on the experiences of patients and their parents to write a white paper on the disease | • The SMA group’s white paper successfully initiated a dialogue between families and specialists, resulting in the identification of different forms of the disease |
| De Moerloose  (2015)  [146] | Ireland | Haemophilia | Patient organizations | • The Irish Haemophilia Society collected patient data to assess the effect of a serious bleed on health-related quality of life. | • Health utility values based on the EQ-5D survey were calculated for 3 patients  • This data can supplement key clinical data to inform HTA bodies and payers of the true impact of haemophilia on the day-to-day lives on patients |
| Genetic Alliance UK  (2014)  [97] | United Kingdom | Rare diseases in general | Patient organizations | • Genetic Alliance UK has developed a project to explore the perspectives of patients and families affected by genetic conditions regarding willingness to accept risk or adverse outcomes with a new treatment | Not specified |
| Ferguson  (2002)  [19] | United States | Gastrointestinal stromal tumor (GIST) | Patient organization | • The Life Raft Group, a patient group started by the husband of a patient, published a study evaluating Gleevec’s effectiveness for GIST patients | • The study collected data on quality of clinical care available at clinical trial centres; attempted to evaluate the sources of information that participants relied on; developed a methodology for participants to serve as their own control group; and introduced a new scale to rate side effect severity from the patients point of view  • The leader of LifeRaftGroup.org states that patient-initiated research helps reduce the lag time that professional researchers experience, helping to get important information to patients faster |
| Ferguson  (2002)  [19] | United States | PXE | Patient organizations | • PXE International was part of the research team working towards identifying the gene responsible for PXE | • The gene was identified and patented by the research team |
| Seminara  (2010)  [24] | United States | UCDs | Patient organizations | • Within the UCDC of the RDCRN, the National Urea Cycle Disorders Foundation has developed and distributed surveys to determine attitudes and barriers to study participation | Not specified |
| Von-Hippel-Lindau Alliance (VHLA)  (2014)  [40] | United States | Von Hippel-Lindau disease (VHL) | Patient organizations | • The Von-Hippel-Lindau Alliance (VHLA) developed a VHL Research Council | Not specified |
| Patient Partner  [20] | Not specified | Rare diseases in general | Patient organizations | • Patient organizations gather data or other information  • Patient organizations lead focus groups or discussion sessions for research | Not specified |
| *Development of or involvement in research organizations* | | | | | |
| Molster  (2012)  [95]  *Proposed* | Australia | Rare diseases in general | Patients and families | • Participants in the Australian Rare Diseases Symposium suggested that patients and families should be involved in all decision-making processes within any collaborations or networks established | Not applicable |
| McCormack  (2013)  [142] | Europe | NMDs | Patients, families, and patient organizations | • Parents of patients and representatives of patient organizations are members of TREAT-NMD’s Project Ethics Council (PEC)  • Responses to questions discussed and other papers produced by the PEC are posted on the TREAT-NMD website | • The PEC has provided guidance in several TREAT-NMD projects, including the development of the network’s Global Registry and has provided guidelines for relations with industry  • The PEC has addressed some of the main issues around rare neuromuscular disease care, diagnosis, treatments and ethics, and demonstrates a potentially suitable model for addressing similar issues in the rare diseases field |
| Fleurence  (2014)  [143] | United States | Rare diseases in general | Patients and patient organizations | • The National Patient-Centered Clinical Research Network (PCORnet) is comprised of 18 patient powered research networks (PPRNs), led by patients in partnership with researchers, and 11 clinical data research networks  • Patient roles within the networks vary, from contributing data and sharing de-identified data for research to involvement in leadership and governance  • Patient organizations are also members of the PPRNs  • Patients are also represented on PCORnet’s Steering Committee and Executive Committee | • Through PCORnet, PPRNs can learn from each other how to increase and retain members, collect data, and prioritize research  • PPRNs have the chance to work directly with larger health care systems and clinical data research networks  • The first phase of PCORnet extends through to September 2015  • The author states that the network will support rapid, efficient, and cost-effective conduct of research |
| Duchange  (2014)  [147] | European Union | Leukodystrophies (LDs) | Patient organizations | • The EU LeukoTreat program, a network established to support therapy development for LDs, created its own ethics committee (The LeukoTreat Ethics Committee or LEC) comprised of clinicians, researchers, law professionals, and representatives from patient organizations  • The purpose of the LEC is to provide ethical management and follow-up regarding projects in the EU LeukoTreat program | Not specified |
| Boon  (2010)  [46] | Netherlands | NMDs | Patient organizations | • The VSN cofounded a number of scientific collaborations (e.g., The European Alliance of neuromuscular disorders associations (EAMDA);  The European Neuromuscular Centre (ENMC)) | • An EAMDA workshop on Duchenne muscular dystrophy led to a coordinated research effort that resulted in the discovery of the gene responsible for the disease  • 160 workshops run by the ENMC have brought together 2000 scientists, with the resulting reports being among the most cited NMD articles |
| Caron-Flinterman  (2005)  [42] | Netherlands | Rare diseases in general | Patient organizations | • 2 patients participate on the Steering Group on Orphan Drugs, which stimulates and facilitates research on, and development of orphan drugs  • Note: The Dutch Steering Committee has been replaced by the Dutch Orphan Drug Network | Not specified |
| Caron-Flinterman  (2005)  [42] | Netherlands | NMDs | Patient organizations | • 4 representatives from the VSN are board members on the Dutch foundation for Neuromuscular Research (SONMZ), which stimulates and communicates research on the causes of, and therapies for, NMDs | Not specified |
| Fleurence  (2014)  [143] | United States | Rare diseases in general | Patient organizations | • Patients are represented on PCORnet’s Steering Committee and Executive Committee | • The first phase of PCORnet extends through to September 2015  • The author states that the network will support rapid, efficient, and cost-effective conduct of research |
| Dunkle  (2010)  [47] | United States | Rare diseases in general | Patient organizations | • NORD and other patient organizations are involved in the NIH’s Therapeutics for Rare and Neglected Diseases (TRND) initiative, which aims to help accelerate treatment development | Not specified |
| Cystic Fibrosis Foundation  (2014)  [148] | United States | CF | Patient organizations | • The Cystic Fibrosis Foundation (CFF) developed the CF Therapeutics Development Network (CF TDN) in collaboration with specialists in CF clinical research  • The Network is comprised of a coordinating Centre, 77 CFF-accredited care centers (i.e. CF Therapeutics Development Centers) and laboratories and interpretation centers (i.e. National Resource Centers) | • The TDN has conducted more than 100 clinical studies since its inception in 1998 |
| Schwartz  (2013)  [39] | United States | Paychyonychia Congenita (PC) | Patient organizations | • Patient organizations established the PC Project, an international collaborative network of patients, medical professionals, and scientists | Not specified |
| Schwartz  (2013)  [39] | United States | PC | Patient organizations | • Members of the PC Project established the International PC Consortium, which connects various researchers and specialists to conduct basic clinical research | Not specified |
| *Dissemination of research-related information* | | | | | |
| Ekins  (2012)  [144] | International  (Note: project started in North America) | Rare diseases in general | Patients, families, and patient organizations | • The Open Drug Discovery Teams app collects Tweets using particular hashtags (e.g., #sanfilipposyndrome)  • Anyone may use the app and curate the content by endorsing or disapproving of each Tweet (or “factoid”) | • The authors anticipate that this app will be used to disseminate information on new scientific developments, to network and discover other researchers, and to provide opportunities for collaboration by highlighting conferences, publications, or laboratory data sharing |
| JISC  (2011)  [54] | United Kingdom | NMDs | Patients | • Patients participated in the Talk Research focus group to discuss if the communication services provided by the Muscular Dystrophy Charity (MDC), now known as Muscular Dystrophy UK, meet the needs of patients and to provide feedback on language, content, and the structure of the MDC websites and publications | • Patients involved in the Talk Research focus group have become involved in other MDC activities such as reviewing grant applications |
| JISC  (2011)  [54] | United Kingdom | NMDs | Patient organizations | • The MDC communication service employs a team of people who have work in research to produce summaries of new research (e.g., lay summaries of clinical trials)  • The MDC is also part of Patient Inform, which provides patients with access to research articles | Not specified |
| Landy  (2012)  [55] | Not specified | Rare diseases in general | Patient organizations | • Patient organizations presented research at scientific conferences, via websites and newsletters, and through the press | Not specified |
| Landy  (2012)  [55] | Not specified | Rare diseases in general | Patient organizations | • Patient organizations helped to prepare research reports/articles | Not specified |
| *Provide funding for research* | | | | | |
| Kelly  (2017)  [149] | International | Cystic Fibrosis (CF) | Patient organizations | • CF patient organizations fund research, including funding one larger project, funding a series of sub-projects on a common theme, partially funding a research project, and indefinitely funding part of a researcher’s salary.  • Common themes across organizations include practicing an open call for research applications, evaluating applications using a peer review process, and placing an increased emphasis on patient engagement. | Not specified |
| Caron-Flinterman  (2005)  [42] | Germany | Retinitis Pigmentosa | Patient organizations | • Pro Retina funds innovative research projects | Not specified |
| Cystic Fibrosis Foundation (CFF)  (2013)  [34] | United States | CF | Patient organizations | • The CFF provides grants to fund research | Not specified |
| De Blieck  (2013)  [35] | United States | JNCL | Patient organizations | • The Batten Disease Support and Research Association (BDRSA) provides funding for the University of Rochester Batten Center’s (URBC’s) research activities, including clinical trials | • A controlled phase II trial has been initiated, with funding provided by the FDA and the BDSRA, to assess the use of oral mycophenolate in ambulatory children with JNCL |
| Dunkle  (2010)  [47] | United States | Rare diseases in general | Patient organizations | • NORD provides grant programs for medical research | Not specified |
| Fleurence  (2014)  [143] | United States | Rare diseases in general | Patient organizations | • PPRNs operating within PCORnet, a national data infrastructure that incorporates electronic health records and administrative, claims and patient-generated data for use in research | • Through PCORnet, PPRNs can learn from each other how to increase and retain members, collect data, and prioritize research  • PPRNs have the chance to work directly with larger health care systems and clinical data research networks  • The first phase of PCORnet extends through to September 2015  • The author states that the network will support rapid, efficient, and cost-effective conduct of research |
| Lasker  (2005)  [49] | United States | Primary Biliary Cirrhosis (PBC) | Patient organizations | • The PBCers Organization fundraises for research | Not specified |
| Von-Hippel-Lindau Alliance (VHLA)  (2014)  [40] | United States | VHL | Patient organizations | • The VHLA awards grants through a competitive research grant application process | • Research supported by the VHLA has resulted in better understanding of the mechanism responsible for VHL tumor development, improved diagnosis and treatment of VHL, and increased average life expectancy of a person with VHL by more than 16 years |
| Landy  (2012)  [55] | Not specified | Rare diseases in general | Patient organizations | • Patient organizations provide investigators with financial support | • Leaders of the organizations surveyed felt their involvement in clinical research had increased the amount of research performed on their condition of interest and had improved the overall quality of the data produced  • They also felt that their involvement had increased participation rates |
| ***Clinical Trials*** | | | | | |
| *Participation as a research subject* | | | | | |
| Patient Partner  [20] | Not specified | Rare diseases in general | Patients | • Patients contribute DNA, cells, or other biological material to biorepositories or databases for use in a trial | Not specified |
| Patient Partner  [20] | Not specified | Rare diseases in general | Patients | • Patients participate in trials testing the effects of a new treatment or drug | Not specified |
| *Assist researchers in the development/conduct of clinical trials* | | | | | |
| O’Mahony  (2014)  [96]  *Proposed* | Europe | Hemophilia | Patients | • In a satellite symposium at the European Haemophilia Consortium (EHC) Congress, it was suggested by a panel member that to ensure the collection of real-world outcomes that are meaningful to patients, patients must work together with all stakeholders to build a credible framework based on their real-world experience  • This framework will ensure that the methods used to collect evidence are robust and reliable and that the evidence collected is in-depth enough to successfully engage with health authorities | Not applicable |
| Bendixen  (2016)  [98]  *Proposed* | United States | DMD | Families | • Families who know of clinical trials should share the information on social media so that others may learn about them | Not applicable |
| Dunkle  (2010)  [47]  *Proposed* | United States | Rare diseases in general | Patient organizations | • The National Organization for Rare Disorders (NORD), an umbrella organization of rare disease patient organizations, identified one of its priorities as developing systems to improve patient access to and participation in clinical trials | Not applicable |
| EURODIS  (2011)  [21] | Europe | Rare diseases in general | Patient organizations | • Patient organizations help to adapt the design of clinical trials to meet patients’ expectations, facilitating adherence and ensuring quality of life is taken into consideration | Not specified |
| EURODIS  (2011)  [21] | Europe | Rare diseases in general | Patient organizations | • Patient organizations discuss results with trial sponsors to contribute to the assessment of treatment benefits | Not specified |
| EURODIS  (2011)  [21] | Europe | Rare diseases in general | Patient organizations | • Patient organizations provide early information to potential participants to ensure inclusion in a trial | Not specified |
| Boon  (2010)  [46] | Netherlands | NMDs | Patient organizations | • The VSN is involved in assisting and initiating clinical trials | Not specified |
| Black  (2011) [22] | United States | Severe myoclonic epilepsy of infancy (SMEI or Dravet syndrome) | Patient organizations | • The IDEA League provides researchers with access to a large cohort of patients for research | Not specified |
| Black  (2011) [22] | United States | SMEI | Patient organizations | • Centres within the IDEA League’s Collaborative Clinical Research and Comprehensive Care Network collaborate to develop clinical research protocols | Not specified |
| Groft  (2013)  [23] | United States | Rare diseases in general | Patient organizations | • The Rare Diseases Clinical Research Network (RDCRN) obtains input from patient organizations on the development of informed consent statements, recruitment strategies, and protocols for research conducted within the Network | Not specified |
| Groft  (2013)  [23] | United States | Rare diseases in general | Patient organizations | • Patient organizations help the RDCRN to identify patient cohorts and recruit patients for studies conducted by the Network | • Researchers in the RDCRN rank help with patient recruitment as one of the top three benefits of interactions with patient organizations [154] |
| Seminara  (2010)  [24] | United States | Urea Cycle Disorders (UCDs) | Patient organizations | • The National Urea Cycle Disorders Foundation is involved in the Urea Cycle Disorders Consortium (UCDC) of the RDCRN  • The foundation is directly involved in the development of protocols, consents, content evaluations, and progress reporting in RDCRN studies | Not specified |
| Patient Partner  [20] | Not specified | Rare diseases in general | Patient organizations | • Patient organizations inform patients of opportunities for taking part in clinical trials | Not specified |
| Patient Partner  [20] | Not specified | Rare diseases in general | Patient organizations | • Patient organizations assist in the development of clinical research protocols | Not specified |
| Patient Partner  [20] | Not specified | Rare diseases in general | Patient organizations | • Patient organizations review funding requests, clinical trial protocols, or patient information to be used in a trial | Not specified |
| Patient Partner  [20] | Not specified | Rare diseases in general | Patient organizations | • Patient organizations provide advice or serve as advisory members on clinical research program committees for the development of a trial | Not specified |
| Patient Partner  [20] | Not specified | Rare diseases in general | Patient organizations | • Patient organizations supply demographic and disease-specific information on members represented by the organization for use in a trial | Not specified |
| *Provide funding for clinical trials* | | | | | |
| Fajac  (2013)  [150] | Europe | CF | Patient organizations | • The European Cystic Fibrosis Society’s Clinical Trial Network (ECFS-CTN) is funded by the ECFS and national patient organizations through the umbrella organization CF Europe | Not specified |
| Groft  (2013)  [23] | United States | Rare diseases in general | Patient organizations | • Patient organizations fund research conducted within the RDCRN, including travel clinics to facilitate patient access to investigators and studies within the RDCRN | • Researchers in the RDCRN ranked direct funding as one of the top three benefits of interactions with patient organizations [154] |
| Patient Partner  [20] | Not specified | Rare diseases in general | Patient organizations | • Patient organizations finance or raise funds for clinical trials | Not specified |
| *Development of or involvement in clinical trial organizations/networks* | | | | | |
| Fajac  (2013)  [150] | Europe | CF | Patient organizations | • The ECFS established a clinical trial network (ECFS-CTN) comprised of 30 selected sites across 11 European countries  • Current initiatives are to set up an investigator-initiated trial, to implement central laboratories for outcome measures, further develop new surrogate end points, and establish a quality-improvement program | Not specified |
| Fajac  (2013)  [150] | Europe | CF | Patient organizations | • A patient organization representative sits on the Executive Committee of the ECFS-CTN and other representatives are invited to meet with the Executive Committee once a year | Not specified |
| Fajac  (2013)  [150] | Europe | CF | Patient organizations | • Patient organizations are invited to ECFS-CTN steering committee meetings twice a year | Not specified |
| Dunkle  (2010)  [47] | United States | Rare diseases in general | Patient organizations | • Patient organizations are involved in the operations, activities, and strategy of the RDCRN  • Organizations are involved in each consortium and form the RDCRN’s Coalition of Patient Advocacy Groups, which participates in network-level discussions and meetings | Not specified |
| Groft  (2013)  [23] | United States | Rare diseases in general | Patient organizations | • 90 patient organizations are members of the RDCRN, forming the “coalition of patient advocacy groups” (CPAG) | Not specified |
| Groft  (2013)  [23] | United States | Rare diseases in general | Patient organizations | • The CPAG chairperson is a member of the RDCRN Steering Committee | Not specified |
| Seminara  (2010)  [24] | United States | UCDs | Patient organizations | • The Executive Director of the National Urea Cycle Disorders Foundation (NUCDF) serves as a voting member on the UCDC of the RDCRN | Not specified |
| *Dissemination of information on clinical trials results* | | | | | |
| Groft  (2013)  [23] | United States | Rare diseases in general | Patient organizations | • Patient organizations translate research results from the RDCRN to patient communities | • Researchers in the RDCRN ranked communication of research activities to the patient community as one of the top three benefits of interactions with patient organizations[154] |
| Seminara  (2010)  [24] | United States | UCDs | Patient organizations | • The National Urea Cycle Disorders Foundation provides information on its website about the importance of research and ongoing UCDC trials  • It also publishes articles on UCDC studies in its newsletter | Not specified |
| Patient Partner  [20] | Not specified | Rare diseases in general | Patient organizations | • Patient organizations translate the results of trials into patient friendly information | Not specified |
| Patient Partner  [20] | Not specified | Rare diseases in general | Patient organizations | • Patient organizations co-write a scientific article on the results of a trial | Not specified |
| Patient Partner  [20] | Not specified | Rare diseases in general | Patient organizations | • Patient organizations review scientific papers on clinical trials | Not specified |
| *Assessment of benefits and harms* | | | | | |
| Genetic Alliance UK  (2014)  [97]  *Proposed* | United Kingdom | Rare diseases in general | Patients | • Patient input on acceptable risk should be imbedded into treatment R&D, from drug design to clinical trials | Not applicable |
| ***Patient reported outcome measures*** | | | | | |
| *Submission of patient reported outcome measures* | | | | | |
| van der Meijden  (2014)  [32] | International  (Australia, Canada, France, Germany, Netherlands, United Kingdom, United States)  Note: study was based out of the Netherlands | Pompe disease | Patients | • The Pompe Survey collected patient reported outcomes from patients instead of gathering clinical outcome measures | • The authors suggest that their assessment of quality of life and participation in daily activity allows for the measurement of patients’ functioning overall and in a way that truly reflects the disease impact |
| Consolaro  (2016)  [106] | Italy | Juvenile idiopathic arthritis, juvenile dermatomyositis, and juvenile autoinflammatory disease | Patients and families | • Patients and their parents regularly complete multidimensional questionnaires that integrate all major parent- and child-reported outcomes (PCROs) into a single tool (Juvenile Arthritis Multidimensional Assessment Report or JAMAR; Juvenile Dermatomyositis Multidimensional Assessment Report or JDMAR; and Juvenile Autoinflammatory Disease Multidimensional Assessment Report (JAIMAR) | • |
| Consolaro  (2016)  [106] | Not specified | JIA | Patients | • Patients have contributed self-reported pain and disease symptoms to studies using smartphones | • Not specified |
| *Participation in a validation study* | | | | | |
| Dell  (2016)  [151] | International  (North America and UK) | Primary Ciliary Dyskinesia | Patients and families | • Patients and families participated in focus groups and open-ended interviews that were used to create a developmentally appropriate, health-related quality-of-life questionnaires (QOL-PCD) for children  • Patients and families also participated in cognitive interviews whilst reviewing the prototype questionnaire | • The QOL-PCD measures were developed and have demonstrated content validity and cross-cultural equivalence for implementation in English-speaking populations |
| Wicks  (2009)  [26] | International  Note: study was based out of the United States | ALS | Patients | • 7 ALS patients answered various questions provided by the researchers  • An extended scale was developed based on their results and then piloted through an online survey provided to Patients Like Me users  • A 1-week retest and a 3-month follow-up survey were also performed by the Patients Like Me users | • 11 new items for the ALSFRS-R were developed based on the feedback from ALS patients  •The extended scale was validated by the Patients Like Me users; however, further validation in real-world studies is necessary |
| Mattsson  (2015)  [110] | Europe | Systemic sclerosis | Patients | • Patients participated in interviews to identify and describe personal factors in the experiences of functioning and health of persons with SSc. These findings were then compared to PROMs identified in SSc research to determine if existing measures cover all factors identified. | • 15 of 35 PROMs did not cover any of the concepts identified by patients  • Few PROMs covered factors relating to “patterns of experience and behaviour”.  • “Motives” and “personal history and biography” were not covered at all. |
| Abdulla  (2013)  [27] | Germany | Amyotrophic Lateral Sclerosis (ALS) | Patients | • Patients completed the self-administered Amyotrophic Lateral Sclerosis Functional Rating Scale (ALSFRS-EX) survey in German | • The ALSFRS-EX was found to have similar psychometric properties to the previously used scale, the revised Amyotrophic Lateral Sclerosis Functional Rating Scale (ALSFRS-R), with high internal consistency and reliability  • Analysis of correlations with other clinical parameters and two other scales demonstrated excellent validity |
| Hoffman  (2008)  [28] | United States | Familial cold auto-inflammatory syndrome (FCAS) and Muckle-Well Syndrome (MWS) | Patients | • Patients completed daily health assessment forms (DHAFs) over 6 months, testing the relevance of symptoms identified by the researchers and the usefulness of different measurement scales  • Subsequently, patients in a phase III clinical trial completed a revised DHAF (based on the results collected in the observational study) during the baseline period  • A Physician’s Global Assessment of Disease Activity was also completed for each patient in the clinical trial for validation purposes | • The first validated patient-reported outcome measure for patients with FCAS or MWS was developed  • The measure has high internal consistency and test-retest reliability, and is highly correlated with overall assessments of disease severity and functional limitations |
| Consolaro  (2016)  [106] | Not specified | JIA, systemic lupus erythematosus, and chronic musculoskeletal pain/juvenile fibromyalgia | Patients | • Patients participate in validation studies for Patient-Reported Outcomes Measurement Information System (PROMIS) measures | • Not specified |
| *Assist in the conduct of a validation study* | | | | | |
| Wicks  (2009)  [26] | International  Note: study was based out of the United States | ALS | Patients | • An ALS patient and member of the Patients Like Me online community was consulted to develop questions around regions of function and patients’ abilities to perform daily living activities | • 11 new items for the ALSFRS-R were developed based on the feedback from ALS patients  •The extended scale was validated by the Patients Like Me users; however, further validation in real-world studies is necessary |
| Kodra  (2007)  [29] | International (Italy, France, Spain, Romania, United Kingdom, and Turkey) | Rare diseases in general | Patient organizations | • Patient organizations translated the questionnaires and distributed them amongst their members | • Completed forms were received from Italy, Romania, the UK, Turkey, France, and Spain  • A tool has been developed that allows for the comparison of patient & family experiences across different disease types, countries and services  • The author suggests this could contribute to the implementation of an international, multidimensional and multi-disease periodic evaluation of subjective patient and family experiences |
| ***Patient registries and biorepositories*** | | | | | |
| *Enrollment in a registry or biorepository* | | | | | |
| Montano  (2007)  [30] | International  (Australia, Austria, Brazil, Canada, Chile, Colombia, Finland, France, Germany, Indonesia, Italy, Japan, Morocco, New Zealand, Poland, Puerto Rico, Saudi Arabia, Spain, Switzerland, Turkey, United Kingdom, United States)  Note: study conducted out of the United States | MPS IVA | Patients | • Patients enrolled in a registry completed a questionnaire on birth and family history; age and onset of diagnosis; signs and symptoms; clinical course from infancy to adulthood; surgical interventions; current height and weight; physical activity; other complaints  • Families or friends helped some adult patients to complete the questionnaire | • The information collected will help to facilitate clinical trials on ERTs, as information on the natural history, rate of progression, and distribution of symptoms in untreated patients is necessary for developing clinical endpoints and judging therapeutic effects  • The authors suggest an annual survey will allow for the collection of more data on the management of patients, efficacy of treatment, and endpoints of clinical trials |
| Pastores  (2007)  [31] | International  (Argentina, Belgium, Brazil, Canada, Chile, Czech Republic, Denmark, France, Germany, Hungary, Ireland, Italy, Japan, Korea, Netherlands, Norway, Poland, Saudi Arabia, Singapore, Slovakia, Taiwan, Turkey, United Kingdom, and United States) | Mucopolysaccharidosis Type I (MPS I) | Patients and families | • Patients are enrolled in a registry voluntarily by their physician, who captures medical history data on the patient’s symptoms and treatments  • Patient or their primary family also complete a health assessment questionnaire (MPS-HAQ) to capture data on daily activities  • Family assistance questions are included to assess the support that patients require in self-care and mobility activities | • Data collected in the registry is available for physicians for possible publication  • Physicians also receive monthly Patient Case Reports, newsletters, and aggregate data reports on the entire registry population  • Based on initial assessments, the Registry provides a large, broad, and representative pool of patients with MPS I  • The data collected may be modified in the future to capture other specific parameters |
| van der Meijden  (2014)  [32] | International  (Australia, Canada, France, Germany, Netherlands, United Kingdom, United States)  Note: study was based out of the Netherlands | Pompe disease | Patients | • Patients enroll in the IPA/Erasmus MC Pompe Survey (Pompe Survey), completing a baseline survey and a follow-up questionnaire every year after  • The Survey includes a Pompe-specific questionnaire as well as three generic questions regarding fatigue, participation in daily life, and health-related quality of life | • The Pompe Survey is now one of the largest databases with consistent follow-up of Pompe patients (children and adults) worldwide  • The Pompe Survey has allowed researchers to quantify disease progression in untreated patients, capture disease impact on daily life, and quantify changes due to treatment (enzyme replacement therapy, ERT) over an extended period of time |
| Consolaro  (2016)  [106] | International  (over 50 countries) | Juvenile idiopathic arthritis (JIA) | Patients and families | • Patients enroll in the registry “Pharmacovigilance in JIA patients treated with biologic agents and/or methotrexate – Pharmachild”  • Participants completes a digital version of the JAMAR questionnaire before their appointments | • Not specified |
| Wang  (2015)  [37] | International | DMD and Becker muscular dystrophy (BMD) | Patients and families | • Patients and families regularly update their results in DuchenneConnect, an online patient self-report registry  • Information is collected on age, age at loss of ambulation, current ambulatory status, drug and supplement usage or quality of life items. | • Surveys using the DuchenneConnect platform have allowed patients, researchers and clinicians to quantify the financial burden of DMD and BMD on families and government, assess family preferences for potential risks and benefits of emerging treatments for DMD, and determine the spectrum of mutations among DMD and BMD patients |
| Mallbris  (2007)  [33] | Sweden | Hereditary angioedema (HAE) | Patients | • Patients complete a questionnaire and telephone interview to capture information on demographics; social, educational, economical and health status; quality of life; family history of HAE; comorbidities; potential trigger factors; medical history; attack characterization; severity of symptoms; efficacy, safety, and outcome of different treatments; and the extent of health and social services use  • Patients also provide a blood sample if they are living near a qualified lab | • The design of the registry permits analysis of subpopulations  • With this registry, scientists will be able to conduct case-control and prospective cohort studies, and may also cross-link the data with other population-based registries |
| Evangelista  (2016)  [152] | United Kingdom | Facioscapulohumeral muscular dystrophy (FSHD) | Patients | • Patients enroll in the UK FSHD patient registry | • The registry has demonstrated utility with the recruitment of patients for a natural history study of infantile onset FSHD, and the longitudinal analysis of patient-related outcomes will provide much-need base-line information to power future trials |
| Cystic Fibrosis Foundation (CFF)  (2013)  [34] | United States | CF | Patients | • Patients provide consent to participate in the CFF registry at accredited care centres, where data is collected on state of residence, height, weight, gender, CF mutations, pulmonary function test results, medication use, and complications related to CF | • The data gathered in the registry is used by health care professionals to improve the delivery of care, study treatment effects, develop care guidelines, and design clinical trials  • The registry also allows people with CF, their families, and health care professionals to compare overall health of patients receiving care at one CF centre with those at other centres |
| De Blieck  (2013)  [35] | United States | JNCL | Patients | • Patients enroll in the URBC contact and natural history registry | • Between 2001-2012, 198 families have enrolled in the registry  •120 children from 99 families have enrolled in a study to validate the Unified Batten Disease Rating Scale (UBDRS)  •Other projects that families have been enrolled in include studies of socio-demographic status, visual –aid skills, attitudes and knowledge about genetic testing, and families’ interests in participating in Phase II safety and tolerability clinical trials  • Through the registry, patients were also recruited to participate in the URBC’s phase II trial and parents were able to provide input regarding trial design (e.g., the feasibility of travel) |
| Richesson  (2009)  [36] | United States | Rare diseases in general | Patients | • Patients provide contact information, self-reported diagnosis, and demographic information to the RDCRN  •Patients receive news of open or planned clinical studies on behalf of the RDCRN (e.g., title of open protocol; eligibility criteria; protocol description; open sites; contact info)  • If interested in participating, patients must contact study personnel  •Patients may also use a toll-free number to enroll or update their information  • Standard post may be used to receive information | • Over 4,000 individuals representing 40+ different rare diseases from 61 different countries were enrolled in the Contact Registry as of 2007  • The number of enrolees varies across the consortia due in part to the promotion of the Contact Registry by certain patient organizations  •The overall study participation rate in 5 RDCRN research consortia was 12% (6-27%), with the rate increasing to 16% (8-42%) and 21% (12-43%) when restricting study eligibility to within 200 miles and 100 miles of a study site, respectively |
| Seminara  (2010)  [24] | United States | UCDs | Patients | • Patients self-registered in the RDCRN Contact Registry receive information about UCDC studies via email | Not specified |
| *Establishment of a registry or biorepository* | | | | | |
| Workman  (2013)  [41] | United States | Rare diseases in general | Patients and patient organizations | • Patients, usually through organizations that receive advice from a scientific board of advisors, establish patient-powered registries (PPRs)  • Patients, families, or patient organizations manage or control data collection, the research agenda for the data, and the translation and dissemination of the research from the data  • Many also have biorepositories  • e.g., DuchenneConnect; Life Raft Group Patient Registry and Tissue Bank | Not specified |
| van der Meijden  (2014)  [32] | International  (Australia, Canada, France, Germany, Netherlands, United Kingdom, United States)  Note: study was based out of the Netherlands | Pompe disease | Patient organizations | • The International Pompe Association (IPA), a federation of Pompe disease patient groups, collaborated with Erasmus MC University Medical Centre to establish the Pompe Survey | • The Pompe Survey is now one of the largest databases with consistent follow-up of Pompe patients (children and adults) worldwide  • The Pompe Survey has allowed researchers to quantify disease progression in untreated patients, capture disease impact on daily life, and quantify changes due to treatment (enzyme replacement therapy, ERT) over an extended period of time |
| Wang  (2015)  [37] | International | DMD and Becker muscular dystrophy (BMD) | Patient organizations | • Parent Project Muscular Dystrophy established DuchenneConnect, an online patient self-report registry, to facilitate pre-recruitment and feasibility studies for industry and collect information about the progression and natural history of the disease. | • DuchenneConnect increases participation by lowering the barrier of entry by eliminating requirements for in-person visits or real-time communications  • Surveys using the DuchenneConnect platform have allowed patients, researchers and clinicians to quantify the financial burden of DMD and BMD on families and government, assess family preferences for potential risks and benefits of emerging treatments for DMD, and determine the spectrum of mutations among DMD and BMD patients |
| TREAT-NMD  (2014)  [38] | Europe | NMDs | Patient organizations | • TREAT-NMD collaborated with clinicians and patient organizations internationally to create registries that aim to facilitate future clinical trials and therapy development  • Registries are governed by a charter, with an oversight committee that includes patient representatives | • The global registries for DMD and spinal muscular atrophy (SMA) are recognized as top resources for trial planning and recruitment  • These registries are available to industry and academic researchers  • They also provide information and feedback to patients, connecting them to the research world |
| TREAT-NMD  (2014)  [38] | Europe | NMDs | Patient organizations | • The EuroBioBank, a TREAT-NMD-integrated resource, is led by EURODIS  • It is dedicated to rare diseases research | • EuroBioBank has been referenced in over 100 research papers  • It has ~400,000 samples available to researchers |
| Evangelista  (2016)  [152] | United Kingdom | FSHD | Patient organizations | • The UK FSHD was developed under the umbrella of the global neuromuscular network TREAT-NMD | • The registry has demonstrated utility with the recruitment of patients for a natural history study of infantile onset FSHD, and the longitudinal analysis of patient-related outcomes will provide much-need base-line information to power future trials |
| Cystic Fibrosis Foundation (CFF)  (2013)  [34] | United States | CF | Patient organizations | • The CFF established a patient registry | • The data gathered in the registry is used by health care professionals to improve the delivery of care, study treatment effects, develop care guidelines, and design clinical trials  • The registry also allows people with CF, their families, and health care professionals to compare overall health of patients receiving care at one CF centre with those at other centres |
| Ferguson  (2002)  [19] | United States | PXE | Patient organizations | • PXE International established a registry and tissue bank | Not specified |
| Schwartz  (2013)  [39] | United States | PC | Patient organizations | • The PC Project established the International PC Research Registry, in which patients provide personal histories and, in return, are provided with physician consultations and genetic testing | Not specified |
| Von-Hippel-Lindau Alliance (VHLA)  (2014)  [40] | United States | VHL | Patient organizations | • The VHLA is planning on establishing a patient registry, which will collect data on: the incidence, prevalence, and natural history of VHL lesions; studies of clinical and environmental cofactors that may influence the natural history of VHL; and studies to predict the development of specific lesion patterns within families | Not applicable |
| Workman  (2013)  [41] | United States | Rare diseases in general | Patient organizations | • Some patient registries collaborate to form patient-powered research networks (PPRNs), developing registries with a shared infrastructure and standardized method of data collection  • Data may be combined for analysis  • e.g., Genetic Alliance Registry and BioBank; Patients Like Me; Registries for All | Not specified |
| Baldo  (2016)  [157] | Not specified | Rare diseases in general | Patient organizations | • There are patient organizations who have invested their own resources in the establishment of new biobanks | Not specified |
| *Design of a registry or biorepository* | | | | | |
| van der Meijden  (2014)  [32] | International  (Australia, Canada, France, Germany, Netherlands, United Kingdom, United States)  Note: study was based out of the Netherlands | Pompe disease | Patients | • The Pompe-specific questionnaire utilized in the Pompe Survey was piloted by a patient panel, which also commented on whether any topics were missing | • The questionnaire has successfully been used in the Pompe Survey to collect data on 408 Pompe patients between 2002 and 2013 |
| Duchange  (2014)  [147] | Europe | Leukodystrophies (LDs) | Patients and patient organizations | • In developing a framework for the LeukoDataBase, the LEC also received input from patients and their families through a survey provided to French families participating in the annual meeting of the European Leukodystrophies Association | • 55 questionnaires were returned and analyzed |
| Duchange  (2014)  [147] | Europe | Leukodystrophies (LDs) | Patient organizations | • One of the first tasks of the LEC was to produce recommendations and documents to frame the LeukoDataBase, a supranational registry for patients with LDs  • Patient representatives on the LEC were responsible for relaying patient expectations  • Ethical issues identified and addressed by the LEC include acknowledging the line between care and research; outlining the informed consent process; providing a clear description of data collected and shared in the database; transparency of the length of data conservation; adapting information to patients’ clinical situations (e.g., minors); obtaining consent from patients already included in national databases; and providing ongoing information to patients regarding the registry | • A charter defining the binding commitments and responsibilities of health professionals regarding the preservation, use and sharing of participants’ data within the LeukoDataBase was developed |
| Rubinstein  (2010)  [154] | United States | Rare diseases in general | Patient organizations | • Patient organizations participated in two days of presentation and breakout sessions at the “Advancing Rare Disease Research: The Intersection of Patient Registries, Bio-specimen Repositories, and Clinical Data” workshop  • Discussions were had on the control of a global registry, access to data, bioethical considerations, and privacy concerns | • The workshop resulted in the production of recommendations for the next steps in producing this global registry |
| Rubinstein  (2012)  [155] | United States | Rare diseases in general | Patient organizations | • Patient organizations participated in discussions at the “Informed Consent Models/ Templates for Rare Disease Registries Linked to Biorepositories” workshop, which focused on developing guidance for contributing registries on the informed consent process  • Topics discussed included: the information that should be provided to patients before they give consent, elements to be included in an informed consent form, and existing templates for short, simple, and clear informed consent forms | • The workshop resulted in the production of recommendations for information to be provided to patient before a consent form is signed in the Global Rare Disease Patient Registry and Data Repository (GRDR) |
| *Maintenance and/or management of a registry or a biorepository* | | | | | |
| Woodward  (2016)  [156] | International | aHUS | Patient organizations | • A representative from the aHUS Alliance joined the Global aHUS Registry scientific advisory board (SAB) to strengthen the collaboration between academic experts and patient organizations in order to further understand patients’ needs and how the aHUS registry could help address them  • One role of the representative is providing input to the SAB on patient priorities, including acting as the interface with other patient organizations, inform the registry of analyses and scientific questions of interest to the patient community, propose, discuss and evaluate programme objectives, and provide ad hoc review of patient-related documents (e.g. informed consent forms) | Not specified |
| Baldo  (2016)  [157] | Italy | Rare diseases in general | Patient organizations | • Patient members of patient organizations participated in roundtable sessions organized by the Telethon Network of Genetic Biobanks (TNGB), a network connecting well-established Italian Genetic Biobanks (GBs), to discuss ethical and legal concerns related to privacy and informed consent, sample ownership, withdrawal of samples and consent, access to samples and data, return of results and incidental findings | • These discussions led to a draft of comprehensive informed consent that became the official model adopted by all biobanks in the Network |
| Baldo  (2016)  [157] | Italy | Rare diseases in general | Patient organizations | • Patient organizations also take part in formal working partnerships with the biobanks in the network, agreeing to 1) identify a representative who keeps associated families and referring clinicians informed of biobank activities and policies, 2) promote recruitment of patients and families, and 3) organize shipment of biospecimens to the assigned biobank.  • The PO decides whether they wish to withdraw samples and related consents or transfer the samples to another biobank when the agreement is not renewed  • Biobank staff work closely with patient organization representatives to define the sample types to be collected and the operative procedures related to their sampling and shipment | • These partnerships have allowed professionals to provide information the helps patients understand 1) the length of diagnosis and research process, 2) measures for sharing data with researchers, 3) procedures for return of results, and 4) the international and national recommendations and norms on regulating biobanks. In turn, patients have raised awareness of the professionals by 1) providing information on their values, priorities, needs, perspectives and expectations concerning the biobank services, and 2) sharing their points of view on ethical issues with regard to informed consent, data sharing and return of findings.  • There are now 13 agreements in place. The TNGH has centralized very rare samples in a unique catalogue. All samples and data collected under these agreements are available to the scientific community. Three scientific papers have been published resulting from the distribution of this data. |
| Evangelista  (2016)  [152] | United Kingdom | FSHD | Patients and patient organizations | • Representatives from patient organizations and patients themselves are members of the registry Steering Committee, which has input on the strategic direction of the registry, acts as a data ccess committee and ensures that the registry acts in the best interest of patients. | • The registry has demonstrated utility with the recruitment of patients for a natural history study of infantile onset FSHD, and the longitudinal analysis of patient-related outcomes will provide much-need base-line information to power future trials |
| Lochmuller  (2009)  [153] | Europe | Rare diseases in general | Patients and patient organizations | • Patients and patient organizations actively participate in sample collection | Not specified |
| Baldo  (2016)  [157] | Not specified | Rare diseases in general | Patients and patient organizations | • Patients and patient organizations are involved in the decision-making governance structure of national and international biobanks or biobank networks | Not specified |
| Lochmuller  (2009)  [153] | Europe | Rare diseases in general | Patient organizations | • Patient organizations participate at the operative level of biorepositories | Not specified |
| TREAT-NMD  (2014)  [38] | Europe | NMDs | Patient organizations | • TREAT-NMD collaborated with clinicians and patient organizations internationally to create registries that aim to facilitate future clinical trials and therapy development  • Registries are governed by a charter, with an oversight committee that includes patient representatives | • The global registries for Duchenne Muscular Dystrophy (DMD) and spinal muscular atrophy (SMA) are recognized as top resources for trial planning and recruitment  • These registries are available to industry and academic researchers  • They also provide information and feedback to patients, connecting them to the research world |
| TREAT-NMD  (2014)  [38] | Europe | NMDs | Patient organizations | • The EuroBioBank, a TREAT-NMD-integrated resource, is led by EURODIS  • It is dedicated to rare diseases research | • EuroBioBank has been referenced in over 100 research papers  • It has ~400,000 samples available to researchers |
| Baldo  (2016)  [157] | Italy | Rare diseases in general | Patient organizations | • The TNGB invited a representative of the rare disease patient organization “UNIAMO FIMR” to join the TNGB Advisory Board and to contribute to the development of the TNGB. | • This has been an effective way for patients to be actively involved in drafting TNGB policies and in sharing their perspectives on procedures concerning ethical issues such as transparency, informed consent, privacy, sample use and transfer, data sharing, commercialization, return of results and incidental findings |
| Landy  (2012)  [55] | Not specified | Rare diseases in general | Patient organizations | • Patient organizations are involved with research registries or biobanks | Not specified |
| *Funding a registry or biorepository* | | | | | |
| Lochmuller  (2009)  [153] | Europe | Rare diseases in general | Patient organizations | • Patient organizations fund biorepositories | Not specified |
| Evangelista  (2016)  [152] | United Kingdom | FSHD | Patient organizations | • The UK FSHD patient registry is funded by Muscular Dystrophy UK | • The registry has demonstrated utility with the recruitment of patients for a natural history study of infantile onset FSHD, and the longitudinal analysis of patient-related outcomes will provide much-need base-line information to power future trials |
| Rubinstein  (2010)  [154] | United States | Rare diseases in general | Patient organizations | • Patient organizations sponsored the “Advancing Rare Disease Research: The Intersection of Patient Registries, Bio-specimen Repositories, and Clinical Data” workshop, which focused on the development of a global patient registry for rare diseases | Not specified |
| *Recruitment of registry or biorepository participants* | | | | | |
| van der Meijden  (2014)  [32] | International  (Australia, Canada, France, Germany, Netherlands, United Kingdom, United States)  Note: study was based out of the Netherlands | Pompe disease | Patient organizations | • The IPA recruits participants to the Pompe Survey by requesting new members of the member support groups to participate | • The Pompe Survey is now one of the largest databases with consistent follow-up of Pompe patients (children and adults) worldwide  • 408 Pompe patients have been tracked in the Pompe Survey between 2002 and 2013 |
| Woodward  (2016)  [156] | International | aHUS | Patient organizations | • Another role of the patient representative on the Global aHUS registry SAB is promoting interest in the Registry among patients with aHUS, including: generating programme awareness and interest within patient community, assisting with the involvement of patient support groups and individual patients, providing information on the aHUS Registry to potential patients, and providing advice and support to patients on aHUS Registry-related manners | Not specified |
| Baldo  (2016)  [157] | Italy | Rare diseases in general | Patient organizations | • Patient organizations also take part in formal working partnerships with the biobanks in the network, agreeing to 1) identify a representative who keeps associated families and referring clinicians informed of biobank activities and policies, 2) promote recruitment of patients and families, and 3) organize shipment of biospecimens to the assigned biobank. The PO decides whether they wish to withdraw samples and related consents or transfer the samples to another biobank when the agreement is not renewed  • Biobank staff work closely with patient organization representatives to define the sample types to be collected and the operative procedures related to their sampling and shipment  • Sample collections are often organized to coincide with the patient organization’s biannual/annual meetings | • These partnerships have allowed professionals to provide information the helps patients understand 1) the length of diagnosis and research process, 2) measures for sharing data with researchers, 3) procedures for return of results, and 4) the international and national recommendations and norms on regulating biobanks. In turn, patients have raised awareness of the professionals by 1) providing information on their values, priorities, needs, perspectives and expectations concerning the biobank services, and 2) sharing their points of view on ethical issues with regard to informed consent, data sharing and return of findings.  • There are now 13 agreements in place. The TNGH has centralized very rare samples in a unique catalogue. All samples and data collected under these agreements are available to the scientific community. Three scientific papers have been published resulting from the distribution of this data. |
| Evangelista  (2016)  [152] | United Kingdom | FSHD | Patient organizations | • Patients find about the UK FSHD registry through patient support and advocacy groups (e.g. FSHD Support UK and Muscular Dystrophy UK), who provide information at conferences, on websites, and in newsletters. | • The registry has demonstrated utility with the recruitment of patients for a natural history study of infantile onset FSHD, and the longitudinal analysis of patient-related outcomes will provide much-need base-line information to power future trials |
| ***Stakeholder relationships and collaborations*** | | | | | |
| *Facilitation of relationships between stakeholders* | | | | | |
| EURODIS  (2011)  [21] | Europe | Rare diseases in general | Patient organizations | • EURODIS, in collaboration with the Alliance Maladies Rares, European experts, and members of the EURODIS Round Table of companies, developed a charter to outline general principles for collaborations between trial sponsors and patient organizations | Not specified |
| Dunkle  (2010)  [47] | United States | Rare diseases in general | Patient organizations | • NORD provides neutral meetings for communication between patients, the FDA, and industry members | Not specified |
| Dunkle  (2010)  [47] | United States | Rare diseases in general | Patient organizations | • NORD interfaces between the FDA and manufacturers of orphan products, communicating to manufacturers information on the orphan product designation and how to apply | Not specified |
| *Establishment and maintenance of relationships with stakeholders* | | | | | |
| Genetic Alliance UK  (2014)  [97]  *Proposed* | United Kingdom | Rare diseases in general | Patients | • Dialogue between patients, manufacturers, regulatory bodies, and clinical researchers should be maintained to ensure that the data required and the data collected throughout R&D, clinical trials, and regulations of new treatments are in line with each other | Not applicable |
| Caron-Flinterman  (2005)  [42] | Netherlands | NMDs | Patients | • Patient develop relationships with researchers, sharing information about their disease symptoms, including their severe fatigue | • This commentary led to the initiation of a research project on the central and peripheral aspects of muscle fatigue associated with NMDs |
| De Blieck  (2013)  [35] | United States | JNCL | Patients | • Patients provide anecdotal reports on their symptoms to URBC researchers | • Based on these reports, studies have been initiated on: the perceived benefit of flupirtine treatment; sex differences in JNCL symptom onset; and rate of progression seizure characteristics, and treatments |
| O’Mahony  (2014)  [96] | European Union | Rare disease in general | Patient organizations | • Patient organizations are members of the European Union Committee of Experts on Rare Diseases (EUCERD), which aims to foster exchange of relevant experience, policies, and practices between all parties (i.e., patient organizations, Ministries of Health, research and public health experts, industry, and the European Commission) with the main goal of aiding the European Commission in the preparation and implementation of community activities in the field of rare diseases | Not specified |
| Baldo  (2016)  [157] | Italy | Rare diseases in general | Patient organizations | • Working partnerships between patient organizations and biobanks have been formalized through written agreements. The TNGB Advisory Board developed and agreement template for use by patient organizations and the 11 biobanks of the Network. | • These partnerships have allowed professionals to provide information the helps patients understand 1) the length of diagnosis and research process, 2) measures for sharing data with researchers, 3) procedures for return of results, and 4) the international and national recommendations and norms on regulating biobanks. In turn, patients have raised awareness of the professionals by 1) providing information on their values, priorities, needs, perspectives and expectations concerning the biobank services, and 2) sharing their points of view on ethical issues with regard to informed consent, data sharing and return of findings.  • There are now 13 agreements in place. The TNGH has centralized very rare samples in a unique catalogue. All samples and data collected under these agreements are available to the scientific community. Three scientific papers have been published resulting from the distribution of this data. |
| Dunkle  (2010)  [47] | United States | Rare diseases in general | Patient organizations | • NORD collaborates with industry, allowing companies to participate on its Corporate Council | Not specified |
| Dunkle  (2010)  [47] | United States | Rare diseases in general | Patient organizations | • NORD’s Medical Advisory Committee allows for the incorporation of the view of health care professionals into their advocacy process | Not specified |
| Dunkle  (2010)  [47] | United States | Rare diseases in general | Patient organizations | • NORD and EURODIS signed a Memorandum of Understanding and are collaborating on several initiatives aiming to increase global awareness, promote R&D on new treatments, and advocating for more compassionate public policies | Not specified |
| Ferguson  (2002)  [19] | United States | GIST | Patient organizations | • The LRG established a Science Team, which reviews medical literature and communicates with leading GIST specialists | Not specified |
| Groft  (2013)  [23] | United States | Rare diseases in general | Patient organizations | • Patient organizations establish global partnerships as part of the RDCRN | Not specified |
| Von-Hippel-Lindau Alliance (VHLA)  (2014)  [40] | United States | VHL | Patient organizations | • The VHLA establishes collaborations with various organizations (e.g., Genetic Alliance, National Organization for Rare Disorders) and acts as the home for the International VHL Alliance, an international network of VHL Affiliates | • The International VHL Alliance allows the VHLA to connect with 90% of all diagnosed VHL patients around the world |
| ***Education*** | | | | | |
| *Informational resources* | | | | | |
| Patient Partner  [20] | International | Rare diseases in general | Patient organizations | • The Patient Partner project, a collaboration involving patient organization alliances (e.g., Genetic Alliance UK), established a set of guidelines for the involvement of patient organizations and patient representatives in clinical research  • The guidelines describe clinical trials and discuss how patients and patient organizations can get involved, who they should become involved with, and ethical considerations for partnerships | Not specified |
| Scarpa  (2011)  [45] | Europe | MPS II | Patient organizations | • Patient organizations provide verbal and written information on issues like education, grants, equipment, care plans, independent living, pre-and post-bereavement support, disability benefits, respite care, and housing | Not specified |
| Boon  (2010)  [46] | Netherlands | NMDs | Patient organizations | • The VSN keeps track of scientific development, annotates them, tries to clarify them, and reports on them with disclaimers to the patient community | Not specified |
| Black  (2011) [22] | United States | SMEI | Patient organizations | • The League has a website containing information on the clinical course, etiology, epidemiology, and treatment of Dravet syndrome and provides links to other international resources and support groups, and to educational financial and health care resources for children with special needs  • Information is available regarding opportunities to fund & participate in research, local events in the Dravet syndrome community, genetic testing, and regional epilepsy centers providing care to patients | Not specified |
| Dunkle  (2010)  [47] | United States | Rare diseases in general | Patient organizations | • Patient organizations created a database of medical information on rare diseases that both patients and health care professionals may access | Not specified |
| Groft  (2013)  [23] | United States | Rare diseases in general | Patient organizations | • Patient organizations are involved in educating patients, public, media, and healthcare providers within the RDCRN | Not specified |
| Groft  (2013)  [23] | United States | Rare diseases in general | Patient organizations | • Patient organizations provide disease-specific information for patients and families on RDCRN website | Not specified |
| Seminara  (2010)  [24] | United States | UCDs | Patient organizations | • The National Urea Cycle Disorders Foundation helped to design content for the UCDC website and develop and distribute brochures to patients, families, and health care professionals | Not specified |
| Von-Hippel-Lindau Alliance (VHLA)  (2014)  [40] | United States | VHL | Patient organizations | • The VHLA publishes a VHL Handbook, providing patients with tips on diagnosis, treatment, and ways of living with VHL | Not specified |
| Von-Hippel-Lindau Alliance (VHLA)  (2014)  [40] | United States | VHL | Patient organizations | • The VHLA answers questions for physicians or passes questions along to appropriate experts | Not specified |
| Von-Hippel-Lindau Alliance (VHLA)  (2014)  [40] | United States | VHL | Patient organizations | • The VHLA provides a toll-free hotline and a mentoring program | Not specified |
| Von-Hippel-Lindau Alliance (VHLA)  (2014)  [40] | United States | VHL | Patient organizations | • The VHLA has a website and sends out monthly wellness e-letters | Not specified |
| *Formal educational activities and training programs* | | | | | |
| Badiu  (2017)  [43] | Europe | Congenital hypogonadotropic hypogonadism and Kallman syndrome | Patients | • Online patient community leaders (i.e. moderators of online patient support sites) worked with the Patient Advocacy Working Group of the European Cooperation in Science and Technology (COST) to identify key patient education material (PEM) content areas and topics based on the most frequently asked questions on social media sites and from previously conducted patient needs assessments  • Patient input was sought at each following stage of PEM development including topic identification, design & revision, and vetting.  • To evaluate end-user perspectives of the PEM, a pdf of the PEM was distributed to patients alongside a PEM Assessment Tool (PEMAT) | • Patient partnerships were used to identify key topics and to target issues most important to patients as well as to contribute content |
| Kodra  (2016)  [44] | Italy | Prader-Willi syndrome (PWS) | Families | • Parents participated in the implementation and evaluation of a pilot PWS psychoeducational parent training program | • Parent training was found to be a promising intervention for parents of children with behaviour problems.  • Interventions with a behaviorally oriented program are useful for increasing parents’ ability to manage problems related to PWS. |
| Black  (2011) [22] | United States | SMEI | Patient organizations | • The IDEA League has organized Grand Rounds on Dravet syndrome at academic institutions and sponsors educational activities for health care professionals | Not specified |
| Cystic Fibrosis Foundation (CFF)  (2013)  [34] | United States | CF | Patient organizations | • The CFF provides education and advocacy resources to patients and their families  • It also provides training in quality improvement | Not specified |
| Groft  (2013)  [23] | United States | Rare diseases in general | Patient organizations | • Patient organizations fund training programs of RDCRN consortia and patient registries  • They also fund training of RDCRN investigators | Not specified |
| Lasker  (2005)  [49] | United States | PBC | Patient organization | • The PBCers Organization provides educational programs | Not specified |
| Seminara  (2010)  [24] | United States | UCDs | Patient organizations | • The National Urea Cycle Disorders Foundation is directly involved in training programs within the RDCRN | Not specified |
| Landy  (2012)  [55] | Not specified | Rare diseases in general | Patient organizations | • Patient organizations sponsored education events, sponsored healthcare professional education events, and educate policy makers | Not specified |
| ***Advocacy and awareness*** | | | | | |
| Advocating for research | | | | | |
| TREAT-NMD  (2014)  [38] | Europe | NMDs | Patient organizations | • Patient organizations (e.g., EURODIS) advocated for funding from the European Union to establish the TREAT-NMD network | • The network has since become a global organization bringing together leading specialists, patient organizations, and industry representatives |
| Caron-Flinterman  (2005)  [42] | Germany | Retinitis Pigmentosa | Patient organizations | • Pro Retina lobbied for public funding for research | Not specified |
| Patient Partner  [20] | Not specified | Rare diseases in general | Patient organizations | • Patient organizations lobby for the development of clinical trials for a specific condition | Not specified |
| *Advocating for drug access/coverage* | | | | | |
| Goodman  (503)  [48] | United States | Myelodysplastic syndrome | Patients and families | • Parents of a 7 year old patient used Facebook to advocate for access to an experimental antiviral therapy produced by Chimerix after their first request made to the company was turned down | • The resulting social media response lead the FDA and Chimerix to collaborate on a small, uncontrolled pilot trial that immediately provided the drug to the 7 year old patient and 19 other patients |
| Black  (2011) [22] | United States | SMEI | Patient organizations | • The League advocated for universal coverage of drugs shown to benefit SMEI patients  • They are still advocating for full approval of stiripentol by the FDA in the United States | • The League has secured coverage of stiripentol and clobazam by 14 Medicaid Agencies and 6+ private insurance companies in the United States; the Ministry of Health in Ontario; and one of the largest HMOs in Israel |
| *Advocating for legislation* | | | | | |
| Dunkle  (2010)  [47] | United States | Rare diseases in general | Patient organizations | • NORD representatives testify before Congressional committees on a regular basis  •Advocacy efforts of NORD have focused on: rare disease and orphan drug legislation; ensuring adequate funding for the FDA, the NIH, and the SSA; extended patent period on orphan products; the removal of lifetime caps from health insurance policies; and for legislation to improve patient access to clinical trials | • The passing of the Orphan Drug Act was due in part to the advocacy efforts of the coalition that would eventual become NORD |
| Lasker  (2005)  [49] | United States | PBC | Patient organization | • The PBCers Organization advocates for those with PBC | Not specified |
| Landy  (2012)  [55] | Not specified | Rare diseases in general | Patient organizations | • Patient organizations lobby policy makers | Not specified |
| *Awareness campaigns* | | | | | |
| Black  (2011) [22] | United States | SMEI | Patient organizations | • The IDEA League participates in national awareness campaigns | Not specified |
| Dunkle  (2010)  [47] | United States | Rare diseases in general | Patient organizations | • Patient organizations created Rare Disease Day | Not specified |
| ***Conferences and Workshops*** | | | | | |
| *Participating in conferences and workshops* | | | | | |
| Molster  (2012)  [95] | Australia | Rare diseases in general | Patients, families and patient organizations | • Patients, families, and patient organizations participated in workshops at the Australian Rare Diseases Symposium discussing the issues, goals, and actions relevant to the development of a national plan | • Participants identified a range of key issues for consideration, with associated goals and actions  • There was agreement that the recommendations drawn out from the outcomes of the workshops should be used to inform and frame the development of a national plan |
| *Hosting conferences and workshops* | | | | | |
| De Blieck  (2013)  [35] | United States | JNCL | Patient organizations | • The BDRSA hosts an Annual Conference | • The conference provides a venue for the URBC to enroll families in the contact registry, the natural history and disease-specific rating scale study, and/or neuropsychological investigations |
| Lasker  (2005)  [49] | United States | PBC | Patient organization | • The PBCers Organization hosts conferences with medical experts | Not specified |
| Seminara  (2010)  [24] | United States | UCDs | Patient organizations | • The National Urea Cycle Disorders Foundation hosts an annual conference where educational presentations are given to patients and professionals by UCDC investigators | • The conference has helped to facilitate recruitment into UCDC studies |
| Cystic Fibrosis Foundation (CFF)  (2013)  [34] | United States | CF | Patient organizations | • The CFF hosts the annual North American CF Conference, where updates on CF care and research are shared | Not specified |
| Von-Hippel-Lindau Alliance (VHLA)  (2014)  [40] | United States | VHL | Patient organizations | •Hosts the International VHL Medical Symposia, bringing together leaders in VHL basic, translational, and clinical researchers, as well as clinicians specializing in VHL diagnosis and treatment | • The conference helps to stimulate research and make connections among professionals |
| *Funding conferences and workshops* | | | | | |
| Groft  (2013)  [23] | United States | Rare diseases in general | Patient organizations | • Patient organizations fund research-based scientific conferences and meetings for patients, families, and families within the RDCRN | Not specified |
| Landy  (2012)  [55] | Not specified | Rare diseases in general | Patient organizations | • Patient organizations organize and support scientific conferences | Not specified |
| ***Patient care and support*** | | | | | |
| *Social support for patients* | | | | | |
| Frost  (2008)  [53] | International | Rare diseases in general | Patients | • Patients participate in Patients Like Me, an online community in which data can be entered into health profiles (e.g., symptoms, treatments, functional rating etc.) and members can communicate via forums and comments on their profiles | • 3,200 members (including families, providers, and researchers) have joined since 2006, with 1,750 patient users  • Data is aggregated from all health profiles to create community summaries on treatments and symptoms |
| Hughes  (2008)  [158] | International | Rare diseases in general | Patients and families | • Patients and their families participate in discussion threads on RareConnect.org, an online patient forum  • Posts tended to fall within one of the following themes: finances, difficulty in getting a diagnosis, feeling that doctors are not knowledgeable, and feelings of isolation | • The author concludes that rare disease patients provide their peers with social support and information  • RareConnect.org provides the opportunity for general information sharing  • However, due to the prevalence of moderators in this community, supportive and sociable relationships are difficult to develop |
| Doyle  (2015)  [140] | United States | Cystinosis | Patients and families | • Through advocacy groups and the Internet, patients and families interact with their disease community in 5 main ways:  1. Comfortability: they connect with others who understand their situation, allowing them to talk freely, feel understood and less guarded, and to receive comfort during difficult times  2. Comparing notes: they exchange ideas about living with the disease, sharing strategies, personal anecdotes, warnings, and information about the disease or treatment  3. Modeling and mentoring: those with more experience are seen as having greater knowledge in living with the illness, becoming models and mentors to those with less experience  4. Witnessing and scaling: they observe each other’s health, progression of illness, etc., and compare it with their own health status; older patients’ experience may provide hope or fear, and can also be used to encourage adherence to treatment  5. Going/being public: they share their story and opinions through Facebook groups, patient organization forums, and other message boards, giving and getting social support | • The authors conclude that patients and their families benefit from peer support and mentorship, and that participating in a disease community helps them to live with their illness |
| Ferguson  (2002)  [19] | United States | GIST | Patients | • The Life Raft Group (LRG) is an online community started by the husband of a patient with a gastrointestinal stromal tumor (GIST)  • A medical librarian collects medical updates from the members and shares them with the group | Not specified |
| Scarpa  (2011)  [45] | Europe | MPS II | Patient organizations | • Patient organizations provide counselling and connect families to other affected individuals | Not specified |
| Scarpa  (2011)  [45] | Europe | MPS II | Patient organizations | • Patient organizations help patients understand their diseases | Not specified |
| Black  (2011) [22] | United Kingdom | SMEI | Patient organizations | • The IDEA League UK established a fund to help families attend their annual “Dravet Weekend Away” | Not specified |
| JISC  (2011)  [54] | United Kingdom | NMDs | Patient organizations | • The MDC as a patient forum as well as a Facebook page and Twitter account | Not specified |
| Black  (2011) [22] | United States | SMEI | Patient organizations | • The IDEA league established an online network allowing for social networking between families in online forums | • The online family forum has helped to identify challenges in the care of Dravet syndrome patients, including access to care, difficulties in diagnosis, treatment disparities among providers, and the need to define associated comorbidities  • Information gathered in the online forums has stimulated research into associated conditions (e.g., facial anomalies) |
| Black  (2011) [22] | United States | SMEI | Patient organizations | • The IDEA League has organized social gathering for patients and their families | Not specified |
| Lasker  (2005)  [49] | United States | PBC | Patient organization | • The PBCers Organization provides electronic mailing lists (listservs), chatrooms and message boards for use by patients and other informational resources | • Patients were found to use the online resources provided by the PBCers Organization, particularly the Daily Digest, to discuss biomedical issues (e.g., medications), but framed within the context of offering support to their peers |
| Von-Hippel-Lindau Alliance (VHLA)  (2014)  [40] | United States | VHL | Patient organizations | • The VHLA hosts an Annual Family Meeting | Not specified |
| Landy  (2012)  [55] | Not specified | Rare diseases in general | Patient organizations | • Patient organizations organize support groups and maintained toll-free support lines | Not specified |
| *Financial support for patients* | | | | | |
| Black  (2011) [22] | United Kingdom | SMEI | Patient organizations | • The IDEA League UK has helped families obtain medical equipment by funding the purchases or helping families to obtain discounts | Not specified |
| Black  (2011) [22] | United States | SMEI | Patient organizations | • The League created a medication assistance fund to help financially disadvantaged families obtain stiripentol when funding is not available | Not specified |
| Dunkle  (2010)  [47] | United States | Rare diseases in general | Patient organizations | • NORD administers Patient Assistance Programs for uninsured and underinsured patients | Not specified |
| Dunkle  (2010)  [47] | United States | Rare diseases in general | Patient organizations | • NORD provides drugs at no charge to eligible patients through its Medication Assistance Programs | Not specified |
| Dunkle  (2010)  [47] | United States | Rare diseases in general | Patient organizations | • NORD provides premium and copayment funds for patients with certain disorders, who cannot afford out-of-pocket costs associated with their plans | Not specified |
| Landy  (2012)  [55] | Not specified | Rare diseases in general | Patient organizations | • Patient organizations provide financial assistance to patients | Not specified |
| *Support for patients participating in clinical trials* | | | | | |
| Bendixen  (2016)  [98]  *Proposed* | United States | DMD | Families | • Families who have participated in a trial should provide peer support for those who are just entering trials | Not applicable |
| Dunkle  (2010)  [47] | United States | Rare diseases in general | Patient organizations | • Patient organizations provide travel and temporary housing assistance to patients and families who must travel to participate in clinical trials | Not specified |
| EURODIS  (2011)  [21] | Europe | Rare diseases in general | Patient organizations | • Patient organizations support patients during a study to reduce the rate of drop-outs and incomplete files | Not specified |
| *Clinical care support for patients* | | | | | |
| Genetic Alliance UK  (2014)  [97]  *Proposed* | United Kingdom | Rare diseases in general | Patients | • Patients, in partnership with their physicians, should decide whether the benefits of a new drug outweigh its risks. | Not applicable |
| Pattacini  (2009)  [50] | Italy | Hemophilia | Patients | • Patients access part of their clinical records through ‘xl’Emofilia, a web-based electronic outpatients’ record, in order to consult their data and record bleeding events and home infusions  • Patient-entered data is validated by doctors at a treating Hemophilia Centre  • Patients receive training to use these records | • Data is extracted in an anonymous format and published on a website  • Some data is also processed and sent to the Italian Registry of Hemophilia and Allied Disorders  • The authors state that this system has improved the management of data, facilitated work, and improved the quality of care for patients |
| Pai  (2016)  [51] | Canada and the United States | Haemophilia | Patients and families | • Patients and families participated on a panel to develop guideline questions and identify patient-important outcomes for the creation of the NHF-McMaster Guideline on Care Models for Haemophilia Management. The panel made recommendations for each guideline question and elaborated on research priorities, implementation considerations, and monitoring. | • Evidence-based clinical practice guidelines were developed for Haemophilia |
| Lane  (2016)  [52] | United States | Haemophilia | Patients and families | • Patients and families participated in interviews related to outcomes, acceptability, equity and feasibility of different care models operating in the US, to inform the creation of the NHF-McMaster Guideline on Care Models for Haemophilia Management. | • Evidence-based clinical practice guidelines were developed for Haemophilia |
| Scarpa  (2011)  [45] | Europe | MPS II | Patient organizations | • A patient organization representative provided input into the development of recommendations through a project led by the Hunter Syndrome European Expert Council | Not specified |
| Scarpa  (2011)  [45] | Europe | MPS II | Patient organizations | • Patient organizations collaborate with physicians, specialist nurses, and homecare companies to make new treatment options available to patients | Not specified |
| TREAT-NMD  (2014)  [38] | Europe | NMDs | Patient organizations | • TREAT-NMD collaborated with specialist groups and patient organizations to develop international consensus documents outlining best practice in diagnosis and patient care  • Patient organizations worked with TREAT-NMD and healthcare professionals to produce patient-friendly summaries of the consensus documents regarding DMD | Not specified |
| Black  (2011) [22] | United States | SMEI | Patient organizations | • The League established the Collaborative Clinical Research and Comprehensive Care Network (CCR-CCN), a network of referral centers providing multidisciplinary care to Dravet syndrome patient  • Centres within the CCR-CCN collaborate to establish more consistent standards of care | Not specified |
| Cystic Fibrosis Foundation (CFF)  (2013)  [34] | United States | CF | Patient organizations | • The CFF provides grants to fund care | Not specified |
| Cystic Fibrosis Foundation (CFF)  (2013)  [34] | United States | CF | Patient organizations | • The CFF established the CF Care Guidelines | Not specified |
| Cystic Fibrosis Foundation (CFF)  (2013)  [34] | United States | CF | Patient organizations | • The CFF provides accreditation to a network of over 110 CF care centres across the United States | Not specified |
| Von-Hippel-Lindau Alliance (VHLA)  (2014)  [40] | United States | VHL | Patient organizations | • Patient organizations established VHL Clinical Care Centres in the United States and around the world | Not specified |
| Von-Hippel-Lindau Alliance (VHLA)  (2014)  [40] | United States | VHL | Patient organizations | • The VHLA created guidelines for screening and treatment  • It also developed a VHL Clinical Advisory Council and created a position of Director of Wellness to respond to questions and concerns; provide connections to medical professionals, etc.; and direct medical questions to the Clinical Advisory Council | Not specified |
| ***Patient organization development*** | | | | | |
| *Establishing patient organizations* | | | | | |
| Rabeharisoa  (2003)  [56] | France | NMDs | Patients and families | • The AFM was established by a mother of 4 patients | • The AFM now has 4,500 members, over 500 salaried workers, and is financially independent  • It is comprised of 3 main departments: “research”; “medical and social action”; and “daily life support”, ensuring that no domain is neglected |
| Rabeharisoa  (2003)  [56] | France | NMDs | Patients and families | • The AFM raises funds to support its work through a television-based initiative (Telethon) | • 80% of the AFM’s annual budget of ~€80 million is raised through Telethon |
| Ferguson  (2002)  [19] | United States | Chromosome 18 deletion | Patients and families | • The mother of a patient developed the Chromosome 18 Registry and Research Society, uniting three independent organizations | Not specified |
| *Providing guidance on establishing patient organizations* | | | | | |
| Mai  (2012)  [57] | United States | LFS | Patient organizations | • At a workshop at the National Institutes of Health, patient organizations shared their perspectives and advice on forming disease-focused support and advocacy groups | Not specified |
| *Establishing international patient organization alliances* | | | | | |
| Woodward  (2016)  [156] | International | aHUS | Patient organizations | • Representatives from 6 European countries (Belgium, France, Italy, Russia, Spain, and the UK) met and agreed to collaborate as the aHUS Alliance. Since then, another 6 organizations have become affiliated with the organization (Australia, Canada, Germany, India, Netherlands, and USA) |  |
| Boon  (2010)  [46] | Netherlands | NMDs | Patient organizations | • The VSN helped to establish the International Pompe Association, an alliance of patient organizations | Not specified |
| Von-Hippel-Lindau Alliance (VHLA)  (2014)  [40] | United States | VHL | Patient organizations | • The VHLA establishes similar alliances around the world | Not specified |
| *Fundraising to support further patient organization growth* | | | | | |
| Von-Hippel-Lindau Alliance (VHLA)  (2014)  [40] | United States | VHL | Patient organizations | • The majority of funds supporting the VHLA are acquired through fundraising initiatives | Not specified |
| ***Regulatory decision-making*** | | | | | |
| *Input into the regulatory decision-making process* | | | | | |
| Genetic Alliance UK  (2014)  [97]  *Proposed* | United Kingdom | Rare diseases in general | Patients | • Patient input on acceptable risk should be imbedded into regulatory decision-making | Not applicable |
| Pharma Letter  (2014)  [111]  *Pilot project* | Canada | Rare diseases in general | Patients and families | • As part of the new Orphan Drug Framework in Canada, patients provide input on how the disease affects their ability to manage their day-to-day lives, what treatments are currently available, what therapeutic benefits are most important to them, and their risk tolerance for new treatments | • This is a pilot project that will be used by Health Canada to assess and refine its approach to collecting patient input, facilitating this process in future orphan drug authorizations |
| EMA  (2012)  [69]  [Website] | European Union | Rare diseases in general | Patient organizations | • Patient organizations are consulted on disease-specific requests by the European Medicines Agency’ scientific committees and working parties  • They take part in discussions on the development and authorization of medicines | Not specified |
| EMA  (2012)  [69]  [Website] | European Union | Rare diseases in general | Patient organizations | • Patient organizations review written information on medicines prepared by the EMA | Not specified |
| EMA  (2012)  [69]  [Website] | European Union | Rare diseases in general | Patient organizations | • Patient organizations are involved in the preparation of EMA guidelines | Not specified |
| EMA  (2012)  [69]  [Website] | European Union | Rare diseases in general | Patient organizations | • Patient organizations take part in EMA conferences and workshops | Not specified |
| FDA  (2017)  [159] | United States | Rare diseases in general | Patients and families | • Patients and families attend public meetings hosted by the FDA where they have the chance to discuss their disease and its impact on their daily lives, the types of treatment benefits that matter to them most, and their perspectives on the adequacy of current therapies | Not specified |
| FDA  (2017)  [159] | United States | Rare diseases in general | Patients and families | • As part of the FDA’s Patient Representative Program, patients and families provide input on disease-specific issues relating to medical products in various stages of development, review and approval | Not specified |
| Black  (2011) [22] | United States | SMEI | Patient organizations | • The IDEA League contributed data used in the orphan drug designation application for stiripentol in the United States | • Stiripentol received orphan drug designation in the United States |
| Dunkle  (2010)  [47] | United States | Rare diseases in general | Patient organizations | • NORD provides input into FDA processes, such as reviewing applications and setting standards for drug and device testing | Not specified |
| *Membership on a regulatory decision-making or advisory committee* | | | | | |
| O’Mahony  (2014)  [96]  *Proposed* | European Union | Haemophilia | Patients and patient organizations | • While patients currently play a role on a number of the EMA’s scientific committees, a panel member at the EHC Congress satellite symposium suggested that there are still further opportunities for patient representation on committees  • e.g., there is not yet patient representation on the Committee for Medicinal Products for Human Use (CHMP) | Not applicable |
| EMA  (2012)  [69]  [Website] | European Union | Rare diseases in general | Patient organizations | • Two representatives from eligible patient organizations are members of the management board of the EMA, which governs the entire organization | Not specified |
| EMA  (2012)  [69]  [Website] | European Union | Rare diseases in general | Patient organizations | • Three representatives from eligible patient organizations are members of the EMA’s Committee for Orphan Medicinal Products (COMP) | Not specified |
| EMA  (2012)  [69]  [Website] | European Union | Rare diseases in general | Patient organizations | • One representative from an eligible patient organization is a member of the Pharmacovigilance Risk Assessment Committee (PRAC) | Not specified |
| EMA  (2012)  [69]  [Website] | European Union | Rare diseases in general | Patient organizations | • Two representatives from eligible patient organizations are members of the Committee for Advanced Therapies | Not specified |
| EMA  (2012)  [69]  [Website] | European Union | Rare diseases in general | Patient organizations | • The representatives from eligible patient organizations are members of the Paediatric Committee (PDCO) | Not specified |
| *Input into the design of the regulatory decision-making process* | | | | | |
| Dunkle  (2010)  [47]  *Proposed* | United States | Rare diseases in general | Patient organizations | • In a summit meeting NORD established that in the future, one priority of the organization should be to identify FDA laws, regulations, and policies that need to be changed to encourage product approval | Not applicable |
| Dunkle  (2010)  [47]  *Proposed* | United States | Rare diseases in general | Patient organizations | • In a summit meeting, NORD established that in the future, one priority of the organization should be to work with the FDA to establish greater certainty in the orphan product approval process, especially in regards to clinical trial design and endpoints | Not applicable |
| ***Reimbursement decision-making*** | | | | | |
| *Input into the reimbursement decision-making process* | | | | | |
| O’Mahony  (2014)  [96]  *Proposed* | European Union | Haemophilia | Patients | • It was stated at the EHC Congress satellite symposium that it is necessary to begin engaging HTA agencies, possibly through EU Network for HTA (EUnetHTA), to allow for patient input at the earliest stage of the HTA process  •It was also suggested by a panel member that there is an opportunity for patient engagement in EUnetHTA’s current efforts to develop common practices across the EU that ensure consistency of data collection  • e.g., it is essential that the system is capable of understanding the impact of an intervention in the context of the experience of hemophilia patients | Not applicable |
| O’Mahony  (2014)  [96]  *Proposed* | European Union | Haemophilia | Patients, families, and patient organizations | • To ensure that health technology assessments (HTAs) consider factors beyond cost (i.e., factors that matter to patients), a panel member at the EHC Congress satellite symposium suggested that patient advocates and clinicians must work together to create a shared consensus on how to effectively evaluate the efficacy of therapies and what experiential data are of the greatest value | Not applicable |
| Genetic Alliance UK  (2014)  [97]  *Proposed* | United Kingdom | Rare diseases in general | Patients | • Nominated patient experts should be permitted to present a summary of the patient evidence submission to the Evaluation Committee | Not applicable |
| Genetic Alliance UK  (2014)  [97]  *Proposed* | United Kingdom | Rare diseases in general | Patients | • Patient should be formally consulted during the National Institute of Health and Care Excellence (NICE) Highly Specialized Technology (HST) evaluation process, prior to the final decision-making Committee meeting, to outline the nature and timeline of any post-evaluation research recommended by NICE | Not applicable |
| Genetic Alliance UK  (2014)  [97]  *Proposed* | United Kingdom | Rare diseases in general | Patients | • If the benefit/risk of a product is to be re-examined by NICE, patient input should be included and all information from patient representatives provided to the EMA for their benefit/risk assessment should be made available to NICE | Not applicable |
| Genetic Alliance UK  (2014)  [97]  *Proposed* | United Kingdom | Rare diseases in general | Patients | • Patient input on benefit/risk should be incorporated during topic selection by the NIHR Horizon Scanning Centre | Not applicable |
| Genetic Alliance UK  (2014)  [97]  *Proposed* | United Kingdom | Rare diseases in general | Patient organizations | • Patient organizations should be involved in the process of topic identification and selection  • Patient organizations play a role in informing NICE of new medicines being considered for market authorization, but a more formalized route may be necessary | Not applicable |
| Dunkle  (2010)  [47]  *Proposed* | United States | Rare diseases in general | Patient organizations | • At a summit meeting, NORD concluded that a future priority for the organization should be to assure reimbursement of off-label drug use for rare disease patients | Not applicable |
| Sussex  (2013)  [112]  *Pilot study* | Europe | Rare diseases in general | Patient organization | • Patient representatives were involved in the pilot stage of multi-criteria decision analysis (MCDA) to establish and apply a framework of weighed attributes to value orphan medicinal products for potential use in the reimbursement decision-making process  • Patient representatives were first interviewed to help identify value attributes for use in the MCDA process  • Patient representatives also participated in a workshop to validate a finalized list of 8 attributes, weight these attributes, and then rate 2 case study OMPs using the weighed attributes (note: two other workshops were hosted by the researchers in this study – one with manufacturers to pilot the process, and a second with clinical and health economics experts) | • The authors suggest that, based on the success of this study, the methods described could be used in real-world settings, by decision-making groups within HTA and reimbursement bodies |
| Winquist  (2014)  [74] | Canada | Rare diseases in general | Patients | • Patients were formally consulted in Ontario’s Drugs for Rare Diseases Working Group’s evaluation of idursulfurase | • Idursulfurase was approved for funding by the Executive Officer of the Ontario Public Drug Programs after risk-sharing agreements were made with the manufacturer |
| Winquist  (2014)  [74] | Canada | Rare diseases in general | Patient organizations | • Upon completion of each drug evaluation between 2008-2013, outcomes of the reviews were shared with patient stakeholder groups to identify any areas of disagreement or error  • Reimbursement guidelines for 3 of the drugs that underwent full evaluation were circulated to patient stakeholder groups and physicians for feedback | • Recommendations were revisited based on the feedback obtained  • The 5 drugs that underwent full evaluation between 2008 -2013 were approved for funding (for certain conditions and with eligibility and exclusion criteria) by the Executive Officer of the Ontario Public Drug Programs after risk-sharing agreements were made with the manufacturer |
| Genetic Alliance UK  (2014)  [97] | United Kingdom | Rare diseases in general | Patients | • “Nominated experts”, including patients and carers, have the opportunity to speak directly to NICE’s Evaluation Committee | Not specified |
| Boon  (2010)  [46] | Netherlands | NMDs | Patient organizations | • The VSN is involved in discussions on reimbursement of therapies for NMDs | Not specified |
| Genetic Alliance UK  (2014)  [97] | United Kingdom | Rare diseases in general | Patient organizations | • Patient representatives provide a patient evidence submission during HST evaluations | Not specified |
| Genetic Alliance UK  (2014)  [97] | United Kingdom | Rare diseases in general | Patient organizations | • Patient groups identified during the evaluation are able to launch an appeal after a guidance has been issued | Not specified |
| NIHR Horizon Scanning Centre  (2015)  [166] | United Kingdom | All diseases, including rare | Patient organizations | • The NIHR Horizon Scanning Centre (HSC) received ad hoc commentary from the Gaucher Association on a briefing report regarding Miglustat for type 3 Guacher disease | Not specified |
| NIHR Horizon Scanning Centre  (2015)  [166] | United Kingdom | All diseases, including rare | Patient organizations | • In 2014, the Genetic Alliance UK agreed to serve as a conduit between the HSC and an appropriate patient group on any genetic topics on which NICE requested briefings  • Subsequently, the Genetic Alliance UK facilitated comments from the MPS Society on an HSC briefing draft regarding Lamazym for alpha-mannosidosis  • The MPS Society made contact with most of the 14 patients with alpha-mannosidosis in the UK, providing some replacement paragraphs with more up-to-date information | • Most of the comments made by the patient group were incorporated into the briefing |
| *Input into the design of the reimbursement decision-making process* | | | | | |
| Genetic Alliance UK  (2014)  [97] | United Kingdom | Rare diseases in general | Patients | • NICE met with patients when developing the initial interim HST framework | Not specified |
| Genetic Alliance UK  (2014)  [97] | United Kingdom | Rare diseases in general | Patient organizations | • Genetic Alliance UK collaborated with other patient organizations to develop a set of recommendations for consideration in the 2014 review of the HST process  • The Alliance also intends on holding consultations and developing another Patient Charter to identify gaps or inconsistencies between the HST evaluation and alternative routes for high cost medicines | Not specified |
| ‡ ”Participants” refers to patients, families, and/or patient organizations  † Within each theme, opportunities are listed in the following order: proposed opportunities for patients and families; opportunities for patients and families; proposed opportunities for patient organizations; and opportunities for patient organizations | | | | | |
