# Supplementary material for: Exploring patient and family involvement in the lifecycle of an orphan drug: a scoping review
Source: Orphanet J Rare Dis. 2017 Dec 22;12:188. doi: 10.1186/s13023-017-0738-6 (PMC5741909; doi:10.1186/s13023-017-0738-6)
Supplement: Supplementary file 2 — Appendix B contains the detailed tabulation of the review of websites of regulatory and reimbursement processes [105–131]. (DOCX 64 kb) [file 13023_2017_738_MOESM2_ESM.docx]

**Appendix B. Results from website review.**

| Table B-1. Opportunities for patient, family, and patient organization involvement in regulatory decision-making identified through website review. | | | | | | | | | |
| --- | --- | --- | --- | --- | --- | --- | --- | --- | --- |
|  |  | **Role** | | | | | | | |
| **Country** | **Regulatory body** | **Provide input in pre-submission advice on protocol** | **Submit patient-reported outcomes (PROs)** | **Membership on advisory or decision-making committees** | **Provide input on proposed regulation decision or guidelines** | **Participate in benefit/harm assessment** | **Provide input on a pharmacovigilance plan** | **Report adverse events** | **Provide input on consumer information (e.g., labelling)** |
| Australia  [58] | Therapeutic Goods Administration (TGA) | No information found (NIF) | NIF | NIF | NIF | NIF | NIF | Yes (Patients) | NIF |
| Canada  [59, 64, 65, 111] | Health Canada | NIF | Yes (Patients) [65] | Yes (Patients) [64,65] | Yes (Patients) [64,65] | NIF | NIF | Yes (Patients) [59] | NIF |
| European Union (Austria, Belgium, Denmark, Finland, France, Germany, Ireland, Italy, Netherland, Norway, Sweden, United Kingdom)  [60, 69, 70, 159] | European Medicines Agency (EMA) | Patient organizations: Yes[69] | Yes (Patients) [159] | Yes (Patient organizations) [69,70] | Yes (Patient organizations) [69] | Yes (Patient organizations) [60] | Yes (Patient organizations) [60] | Yes (Patients) [60] | Yes (Patient organizations) [69] |
| Japan  [113] | Pharmaceuticals and Medical Devices Agency | NIF | NIF | NIF | NIF | NIF | NIF | NIF | NIF |
| New Zealand  [61, 66, 114, 160] | New Zealand Medicines and Medical Devices Safety Authority (Medsafe) | NIF | Yes (Patients) [160] | No [[114] | Yes (Patients) [66] | NIF | NIF | Yes (Patients) [61] | NIF |
| Singapore  [115, 116, 117] | The Health Sciences Authority (HSA) | NIF | NIF | No | NIF | NIF | NIF | No [117] | NIF |
| Switzerland  [62, 118] | Swiss Agency for Therapeutic Products (Swissmedic) | NIF | NIF | No [118] | NIF | NIF | NIF | Yes (Patients) [62] | No |
| United States  [63, 67, 68, 119, 161, 162] | Food and Drug Administration (FDA) | NIF | Yes (Patients) [161] | Yes (Patients) [67,68] | Yes (Patients) [67,68] | Yes (Patients) [162] | NIF | Yes (Patients) [63] | NIF |

| Table B-2. Opportunities for patient, family, and patient organization involvement in reimbursement decision-making identified through website review. | | | | | | | | |
| --- | --- | --- | --- | --- | --- | --- | --- | --- |
|  |  | **Role** | | | | | | |
| **Country** | **Reimbursement body** | **Submit topic for consideration** | **Submit information during preparation of evaluation report** | **Directly consulted during the review process** | **Membership on advisory or decision-making committee** | **Provide feedback on evaluation report and/or proposed recommendations** | **Prepare a “patient submission”** | **Present views during committee** |
| Australia  [75, 79, 80, 81, 168] | Pharmaceutical Benefits Scheme (CDR) | Yes (Patients or patient organizations) [75] | No information found (NIF) | Yes (Patient organizations) [168] | No [75] | NIF | Yes (Patients or patient organizations)  [75, 79] | NIF |
|  | Life Saving Drugs Program (safety-net) | No (Physicians must submit) [80] | Yes (Patient data is submitted for yearly reapplication) [80] | NIF | No [75] | NIF | N/A | NIF |
|  | Highly Specialised Drugs Program (safety-net)^†^ | No [81] | NIF | NIF | No [75] | NIF | N/A | NIF |
| Austria  [71, 120, 121] | CDR | No [130] | NIF | NIF | No [121] | NIF | NIF | NIF |
|  | Individual reimbursement (safety-net)^†§^ | No [71] | NIF | NIF | No [121] | NIF | N/A | NIF |
| Belgium | CDR | NIF | No [122] | No [122] | No [122] | No [122] | No [122] | No [122] |
| [122] | Special Solidarity Fund (safety-net)^†^ | NIF | NIF | NIF | NIF | NIF | NIF | NIF |
| Canada  [78, 164, 175] | Common Drug Review (CDR) | NIF | NIF | NIF | No [175] | NIF | Yes (Patient organizations) [78] | NIF |
|  | pan-Canadian Oncology Drug Review (pCODR) | NIF | NIF | NIF | Yes (Patients) [164] | NIF | Yes (Patient organizations) [164] | NIF |
| Denmark | CDR | No [82] | NIF | NIF | NIF | NIF | NIF | NIF |
| [82] | Individual reimbursement (safety-net)^†^ | No (Physicians must submit) [82] | NIF | NIF | NIF | NIF | N/A | NIF |
| Finland  [90, 123] | CDR | NIF | NIF | NIF | No [123] | NIF | NIF | NIF |
|  | Special License Procedure (safety-net)^‡^ | Yes (Patients) [90] | NIF | NIF | NIF | NIF | N/A | NIF |
| France  [83, 124, 125] | CDR | No [124] | NIF | NIF | No[125] | NIF | NIF | NIF |
|  | Temporary use authorization – individual (safety-net)^‡^ | No [83] | NIF | NIF | NIF | NIF | N/A | NIF |
|  | Temporary use authorization – cohort (safety-net) ^‡^ | No [83] | NIF | NIF | NIF | NIF | NIF | NIF |
| Germany  [84, 126, 127, 169] | CDR | NIF | NIF | Yes (Patient organizations) [126, 169] | No [127] | NIF | NIF | NIF |
|  | Compassionate Use (safety-net)^§^ | No [84] | NIF | NIF | NIF | NIF | N/A | NIF |
| Ireland  [85] | Community Drugs Scheme (CDR) | NIF | NIF | NIF | NIF | NIF | NIF | NIF |
|  | Named Patient Regime (safety-net)^‡^ | No [85] | NIF | NIF | NIF | NIF | N/A | NIF |
| Italy | CDR | NIF | NIF | NIF | NIF | NIF | NIF | NIF |
| [128] | Individual or cohort reimbursement (safety-net)^§‡^ |  |  | NIF | NIF | NIF |  | NIF |
|  | Temporary individual reimbursement (safety-net)^‡^ | NIF | NIF | NIF | NIF | NIF | N/A | NIF |
|  | Individual reimbursement (safety-net)^§‡^ | NIF | NIF | NIF | NIF | NIF | N/A | NIF |
| Japan | CDR | NIF | NIF | NIF | No | NIF | NIF | NIF |
| Netherlands  [71] | Medicines Reimbursement System (CDR) | No [71] | Yes [71] | NIF | Yes (Patients) [71] | NIF | NIF | NIF |
|  | Safety-net^‡^ | NIF | NIF | NIF | NIF | NIF | N/A | NIF |
| New Zealand  [72, 86, 129, 163] | Pharmaceutical Schedule (CDR) | Yes (Patients or patient organizations) [163] | Yes (Patients) [72] | Yes (Patients) [72] | No [129 | Yes (Patients) [72] | NIF | NIF |
|  | Named Patient Pharmaceutical Assessment (Exceptional  Circumstances) Policy (safety-net)^†^ | No [86] | NIF | NIF | No [129] | NIF | N/A | NIF |
| Norway | CDR | No [130] | NIF | NIF | NIF | NIF | NIF | NIF |
| [130] | Compassionate use program (safety-net)^†^ | NIF | NIF | NIF | NIF | NIF | N/A | NIF |
| Singapore  [115] | CDR | NIF | NIF | NIF | No [115] | NIF | NIF | NIF |
| Spain | CDR | NIF | NIF | NIF | NIF | NIF | NIF | NIF |
| Sweden  [171, 120] | CDR | No [120] | NIF | NIF | Yes (Patient organizations) [171] | NIF | NIF | NIF |
| Switzerland  [172] | List of Specialties (CDR) | NIF | NIF | NIF | Yes (Individuals nominated by patient organizations) [172] | NIF | NIF | NIF |
| United Kingdom  [76, 87, 173, 176] | Highly Specialised Drugs Programme (CDR) | NIF | NIF | Yes (Patient organizations) [173] | Yes (Patient organizations) [173] | NIF | Yes (Patients and patient organizations) [176] | Yes (Patients) [76] |
|  | Patient Access Scheme (safety-net)^†^ | NIF | NIF | Yes (Patients) [87] | NIF | Yes (Patients) [87] | NIF | Yes (Patients) [87] |
| Scotland  [25, 88, 131, 170, 177] | CDR | NIF | NIF | Yes (Patient organizations) [170] | No [25] | NIF | Yes (Patient organizations) [177] | NIF |
|  | Patient Access Scheme (safety-net)^†^ | No [88] | NIF | NIF | NIF | NIF | NIF | NIF |
| Wales  [77, 89] | CDR | NIF | NIF | NIF | No [77] | NIF | Yes (Patients and patient organizations) [77] | NIF |
|  | Patient Access Scheme (safety-net)^†^ | No [89] | NIF | NIF | N [89] | NIF | NIF | NIF |
| United States  [73, 165, 174] | CDR | NIF | Yes (Patients) [73] | Yes (Patients) [73] | Yes (“Experts in patient advocacy”) [174] | Yes (Patients) [165] | NIF | Yes (Patients)  [73, 174] |
| ^†^ Non-reimbursed therapies  ^§^ Off-label therapies  ^‡^ Unlicensed therapies | | | | | | | | |
