# Supplementary material for: Exploring patient and family involvement in the lifecycle of an orphan drug: a scoping review
Source: Orphanet J Rare Dis. 2017 Dec 22;12:188. doi: 10.1186/s13023-017-0738-6 (PMC5741909; doi:10.1186/s13023-017-0738-6)
Supplement: Supplementary file 3 — Appendix C contains definitions of the themes identified. (DOCX 34 kb) [file 13023_2017_738_MOESM3_ESM.docx]

**Appendix C. Definitions of the identified themes.**

| **Table C-1. Identified themes and their definitions.** | |
| --- | --- |
| **Theme** | **Definition** |
| Research | Activities, other than clinical trials, related to the conduct of clinical and health services research, including enrollment, study design, funding, and dissemination of results. |
| Clinical trials | Activities related to the conduct of clinical trials, specifically, including enrollment, trial design, funding, and dissemination of results. |
| Patient reported outcome measures | Activities related to the development and use of patient-reported outcome measures. |
| Patient registries and biorepositories | Activities related to the establishment, management, and ongoing use of registries and biorepositories. |
| Stakeholder relationships and collaborations | Activities related to the establishment of collaborations and formal partnerships among various stakeholders. |
| Education | Activities related to the provision of informational resources and formal educational activities on various topics. |
| Advocacy and awareness | Activities related to raising awareness of a disease and advocating for the needs of the disease community. |
| Conferences and workshops | Activities related to hosting and participating in conferences and workshops that unite different stakeholders for various purposes. |
| Patient care and support | Activities related to clinical care and other forms of support for patients and their families. |
| Patient organization development | Activities related to the establishment and growth of rare disease patient organizations and alliances. |
| Regulatory decision-making | Activities related to government decision-making on the approval of new therapies for sale within their jurisdiction. |
| Reimbursement decision-making | Activities related to government decision-making on the funding of new therapies within their jurisdiction. |
