# Supplementary material for: Exploring patient and family involvement in the lifecycle of an orphan drug: a scoping review
Source: Orphanet J Rare Dis. 2017 Dec 22;12:188. doi: 10.1186/s13023-017-0738-6 (PMC5741909; doi:10.1186/s13023-017-0738-6)

**Appendix D. Opportunities mapped across the orphan drug lifecycle.**

| Table D-1. Existing and proposed opportunities for patient, family, and patient organization involvement mapped across the orphan drug lifecycle. | | | |
| --- | --- | --- | --- |
|  | **Participants** | | |
| **Lifecycle stage** | **Patients** | **Families** | **Patient organizations** |
| **Pre-clinical phase** | **Research**  Literature (existing):  • Participating as subjects in studies outside of clinical trials  • Setting research priorities  • Initiating research studies  • Providing assistance to researchers conducting studies  • Leading research  • Participating in research organizations/networks  • Disseminating research-related information  Literature (proposed):  • Participate in all decisions about research on rare diseases  • Participate in decision-making processes within research collaborations/networks  Webinar (existing):  • Funding research  • Providing input on desired format for a new drug  **Patient reported outcome measures (PROMs)**  Literature (existing):  • Participating in studies to develop and validate PROMs  • Assisting researchers in conducting studies to develop and validate PROMs  **Patient registries and biorepositories**  Literature (existing):  • Enrolling in and submitting data to registries and biorepositories  • Providing input on the design of registries  Webinar (existing):  • Providing funding  • Encouraging others to enroll  **Stakeholder relationships and collaborations**  Webinar (existing):  • Facilitating relationships between stakeholders  • Establishing relationships with stakeholders  **Education**  Literature (existing):  • Helping to develop educational material/training programs for patients/families  Webinar (existing):  • Organizing and sponsoring formal educational activities and training programs for health care professionals, researchers, and policymakers  • Participating in standardized patient programs  **Advocacy and Awareness**  Webinar (existing):  • Advocating for research  • Starting awareness campaigns (e.g., fundraisers)  **Conferences and workshops**  Literature (existing):  • Participating in conferences and workshops  Webinar (existing):  • Presenting at conferences  • Sitting on organizing committees  **Patient care and support**  Literature (existing):  • Providing social support  • Monitoring own clinical care  Webinar (existing):  • Providing clinical care support  **Patient organization development**  Literature (existing):  • Establishing patient organizations | **Research**  Literature (existing):  • Initiating research studies  • Providing assistance to researchers conducting studies  • Leading research  • Participating in research organizations/networks  • Disseminating research-related information  Literature (proposed):  • Participate in all decisions about research on rare diseases  • Participate in decision-making processes within research collaborations/networks  Webinar (existing):  • Funding research  **Patient registries and biorepositories**  Literature (existing):  • Submitting data to registries and biorepositories on behalf of a patient  • Providing input on the design of registries  Webinar (existing):  • Providing funding  • Encouraging others to enroll  **Stakeholder relationships and collaborations**  Webinar (existing):  • Facilitating relationships between stakeholders  • Establishing relationships with stakeholders  **Education**  Literature (existing):  • Helping to develop educational material/training programs for patients/families  Webinar (existing):  • Organizing and sponsoring formal educational activities and training programs for health care professionals, researchers, and policymakers  • Participating in standardized patient programs  **Advocacy and Awareness**  Webinar (existing):  • Advocating for research  • Starting awareness campaigns (e.g., fundraisers)  **Conferences and workshops**  Literature (existing):  • Participating in conferences and workshops  Webinar (existing):  • Volunteering at conferences  **Patient care and support**  Literature (existing):  • Providing social support  **Patient organization development**  Literature (existing):  • Establishing patient organizations | **Research**  Literature (existing):  • Participating as subjects in studies outside of clinical trials  • Setting research priorities  • Initiating research studies  • Providing assistance to researchers conducting studies  • Leading research  • Funding research  • Participating in research organizations/networks  • Disseminating research-related information  **Patient reported outcome measures**  Literature (existing):  • Assisting researchers in conducting studies to develop and validate PROMs  **Patient registries and biorepositories**  Literature (existing):  • Providing input on the design of registries  • Serving on oversight committees  • Providing funding  • Recruiting participants  **Stakeholder relationships and collaborations**  Literature (existing):  • Facilitating relationships between stakeholders  • Establishing relationships with stakeholders  **Education**  Literature (existing):  • Sharing informational resources on disease-specific topics  • Organizing and sponsoring formal educational activities and training programs for health care professionals, researchers, and policymakers  **Advocacy and Awareness**  Literature (existing):  • Advocating for research  • Advocating for legislation  • Starting awareness campaigns  Webinar (existing):  • Advocating for improved quality of life  • Advocating for education  • Advocating for clinics and patient care services  • Facilitating patients’ individual Advocacy and Awareness efforts  **Conferences and workshops**  Literature (existing):  • Participating in conferences and workshops  • Organizing conferences  • Funding conferences  **Patient care and support**  Literature (existing):  • Providing social support  • Providing financial support  • Providing clinical care support  **Patient organization development**  Literature (existing):  • Providing advice on develop patient organizations  • Fundraising to support further development of the organization |
| **Clinical trials** | **Research**  Literature (existing):  • Participating as subjects in studies outside of clinical trials  • Setting research priorities  • Initiating research studies  • Providing assistance to researchers conducting studies  • Leading research  • Participating in research organizations/networks  • Disseminating research-related information  Literature (proposed):  • Participate in all decisions about research on rare diseases  • Participate in decision-making processes within research collaborations/networks  Webinar (existing):  • Funding research  **Clinical trials**  Literature (existing):  • Participating as subjects in trials  Literature (proposed):  *•* Ensure the collection of real-world outcomes that are meaningful to patients  **Patient reported outcome measures (PROMs)**  Literature (existing):  • Submitting PROs in a study  • Participating in studies to develop and validate PROMs  • Assisting researchers in conducting studies to develop and validate PROMs  **Patient registries and biorepositories**  Literature (existing):  • Enrolling in and submitting data to registries and biorepositories  • Providing input on the design of registries  Webinar (existing):  • Providing funding  • Encouraging others to enroll  **Stakeholder relationships and collaborations**  Webinar (existing):  • Facilitating relationships between stakeholders  • Establishing relationships with stakeholders  **Education**  Literature (existing):  • Helping to develop educational material/training programs for patients/families  Webinar (existing):  • Organizing and sponsoring formal educational activities and training programs for health care professionals, researchers, and policymakers  • Participating in standardized patient programs  **Advocacy and Awareness**  Literature (existing):  • Advocating for drug access/coverage  Webinar (existing):  • Advocating for research  • Starting awareness campaigns (e.g., fundraisers)  **Conferences and workshops**  Literature (existing):  • Participating in conferences and workshops  Webinar (existing):  • Presenting at conferences  • Sitting on organizing committees  **Patient care and support**  Literature (existing):  • Providing social support  • Monitoring own clinical care  Webinar (existing):  • Providing clinical care support  **Patient organization development**  Literature (existing):  • Establishing patient organizations | **Research**  Literature (existing):  • Initiating research studies  • Providing assistance to researchers conducting studies  • Leading research  • Participating in research organizations/networks  • Disseminating research-related information  Literature (proposed):  • Participate in all decisions about research on rare diseases  • Participate in decision-making processes within research collaborations/networks  Webinar (existing):  • Funding research  **Patient registries and biorepositories**  Literature (existing):  • Submitting data to registries and biorepositories on behalf of a patient  • Providing input on the design of registries  Webinar (existing):  • Providing funding  • Encouraging others to enroll  **Stakeholder relationships and collaborations**  Webinar (existing):  • Facilitating relationships between stakeholders  • Establishing relationships with stakeholders  **Education**  Literature (existing):  • Helping to develop educational material/training programs for patients/families  Webinar (existing):  • Organizing and sponsoring formal educational activities and training programs for health care professionals, researchers, and policymakers  • Participating in standardized patient programs  **Advocacy and Awareness**  Literature (existing):  • Advocating for drug access/coverage  Webinar (existing):  • Advocating for research  • Starting awareness campaigns (e.g., fundraisers)  **Conferences and workshops**  Literature (existing):  • Participating in conferences and workshops  Webinar (existing):  • Volunteering at conferences  **Patient care and support**  Literature (existing):  • Providing social support  **Patient organization development**  Literature (existing):  • Establishing patient organizations | **Research**  Literature (existing):  • Participating as subjects in studies outside of clinical trials  • Setting research priorities  • Initiating research studies  • Providing assistance to researchers conducting studies  • Leading research  • Funding research  • Participating in research organizations/networks  • Disseminating research-related information  **Clinical trials**  Literature (existing):  • Providing assistance to researchers conducting a trial  • Funding clinical trials and clinical trial networks  • Establishing and/or participating in clinical trial networks  • Disseminating information on the results of clinical trials  **Patient reported outcome measures**  Literature (existing):  • Assisting researchers in conducting studies to develop and validate PROMs  **Patient registries and biorepositories**  Literature (existing):  • Providing input on the design of registries  • Serving on oversight committees  • Providing funding  • Recruiting participants  **Stakeholder relationships and collaborations**  Literature (existing):  • Facilitating relationships between stakeholders  • Establishing relationships with stakeholders  **Education**  Literature (existing):  • Sharing informational resources on disease-specific topics  • Organizing and sponsoring formal educational activities and training programs for health care professionals, researchers, and policymakers  • Starting awareness campaigns  **Advocacy and Awareness**  Literature (existing):  • Advocating for drug access/coverage  • Advocating for research  • Advocating for legislation  Webinar (existing):  • Advocating for improved quality of life  • Advocating for education  • Advocating for clinics and patient care services  • Facilitating patients’ individual Advocacy and Awareness efforts  **Conferences and workshops**  Literature (existing):  • Participating in conferences and workshops  • Organizing conferences  • Funding conferences  **Patient care and support**  Literature (existing):  • Providing social support  • Providing financial support  • Providing clinical care support  • Providing support to patients participating in clinical trials  **Patient organization development**  Literature (existing):  • Providing advice on develop patient organizations  • Fundraising to support further development of the organization  **Regulatory decision-making**  Literature (existing):  • Provide input in pre-submission advice given on trial protocol |
| **Regulatory approval** | **Research**  Literature (existing):  • Participating as subjects in studies outside of clinical trials  • Setting research priorities  • Initiating research studies  • Providing assistance to researchers conducting studies  • Leading research  • Participating in research organizations/networks  • Disseminating research-related information  Literature (proposed):  • Participate in all decisions about research on rare diseases  • Participate in decision-making processes within research collaborations/networks  Webinar (existing):  • Funding research  **Patient reported outcome measures (PROMs)**  Literature (existing):  • Submitting PROs in a study  • Participating in studies to develop and validate PROMs  • Assisting researchers in conducting studies to develop and validate PROMs  **Patient registries and biorepositories**  Literature (existing):  • Enrolling in and submitting data to registries and biorepositories  • Providing input on the design of registries  Webinar (existing):  • Providing funding  • Encouraging others to enroll  **Stakeholder relationships and collaborations**  Webinar (existing):  • Facilitating relationships between stakeholders  • Establishing relationships with stakeholders  **Education**  Literature (existing):  • Helping to develop educational material/training programs for patients/families  Webinar (existing):  • Organizing and sponsoring formal educational activities and training programs for health care professionals, researchers, and policymakers  • Participating in standardized patient programs  **Advocacy and Awareness**  Literature (existing):  • Advocating for drug access/coverage  Webinar (existing):  • Advocating for research  • Starting awareness campaigns (e.g., fundraisers)  **Conferences and workshops**  Literature (existing):  • Participating in conferences and workshops  Webinar (existing):  • Presenting at conferences  • Sitting on organizing committees  **Patient care and support**  Literature (existing):  • Providing social support  • Monitoring own clinical care  Webinar (existing):  • Providing clinical care support  **Patient organization development**  Literature (existing):  • Establishing patient organizations  **Regulatory decision-making**  Literature (existing):  • Consideration of PROs by decision-makers  • Membership on advisory or decision-making committees  • Providing input on proposed regulation decision or guidelines  • Participate in benefit/harm assessment  • Reporting adverse events  Literature (proposed):  *•* Provide input on acceptable risk | **Research**  Literature (existing):  • Initiating research studies  • Providing assistance to researchers conducting studies  • Leading research  • Participating in research organizations/networks  • Disseminating research-related information  Literature (proposed):  • Participate in all decisions about research on rare diseases  • Participate in decision-making processes within research collaborations/networks  Webinar (existing):  • Funding research  **Patient registries and biorepositories**  Literature (existing):  • Submitting data to registries and biorepositories on behalf of a patient  • Providing input on the design of registries  Webinar (existing):  • Providing funding  • Encouraging others to enroll  **Stakeholder relationships and collaborations**  Webinar (existing):  • Facilitating relationships between stakeholders  • Establishing relationships with stakeholders  **Education**  Literature (existing):  • Helping to develop educational material/training programs for patients/families  Webinar (existing):  • Organizing and sponsoring formal educational activities and training programs for health care professionals, researchers, and policymakers  • Participating in standardized patient programs  **Advocacy and Awareness**  Literature (existing):  • Advocating for drug access/coverage  Webinar (existing):  • Advocating for research  • Starting awareness campaigns (e.g., fundraisers)  **Conferences and workshops**  Literature (existing):  • Participating in conferences and workshops  Webinar (existing):  • Volunteering at conferences  **Patient care and support**  Literature (existing):  • Providing social support  **Patient organization development**  Literature (existing):  • Establishing patient organizations | **Research**  Literature (existing):  • Participating as subjects in studies outside of clinical trials  • Setting research priorities  • Initiating research studies  • Providing assistance to researchers conducting studies  • Leading research  • Funding research  • Participating in research organizations/networks  • Disseminating research-related information  **Patient reported outcome measures**  Literature (existing):  • Assisting researchers in conducting studies to develop and validate PROMs  **Patient registries and biorepositories**  Literature (existing):  • Providing input on the design of registries  • Serving on oversight committees  • Providing funding  • Recruiting participants  **Stakeholder relationships and collaborations**  Literature (existing):  • Facilitating relationships between stakeholders  • Establishing relationships with stakeholders  **Education**  Literature (existing):  • Sharing informational resources on disease-specific topics  • Organizing and sponsoring formal educational activities and training programs for health care professionals, researchers, and policymakers  • Starting awareness campaigns  **Advocacy and Awareness**  Literature (existing):  • Advocating for drug access/coverage  • Advocating for research  • Advocating for legislation  Webinar (existing):  • Advocating for improved quality of life  • Advocating for education  • Advocating for clinics and patient care services  • Facilitating patients’ individual Advocacy and Awareness efforts  **Conferences and workshops**  Literature (existing):  • Participating in conferences and workshops  • Organizing conferences  • Funding conferences  **Patient care and support**  Literature (existing):  • Providing social support  • Providing financial support  • Providing clinical care support  **Patient organization development**  Literature (existing):  • Providing advice on develop patient organizations  • Fundraising to support further development of the organization  **Regulatory decision-making**  Literature (existing):  • Providing data for orphan drug designation applications  • Provide input in pre-submission advice given on trial protocol  • Membership on advisory or decision-making committees  • Providing input on proposed regulation decision or guidelines  • Participate in the assessment of benefits and harms  • Providing input on plans for post-market approval pharmacovigilance  • Providing input on consumer information (e.g., labelling)  Literature (proposed):  • Participate on regulatory committees that do not currently have patient representation  • Identify laws, regulations, and policies that need to be changed in order to encourage product approval  • Consult with regulatory bodies to establish greater certainty in the approval process, especially in regards to trial design and endpoint selection |
| **Real world studies** | **Research**  Literature (existing):  • Participating as subjects in studies outside of clinical trials  • Setting research priorities  • Initiating research studies  • Providing assistance to researchers conducting studies  • Leading research  • Participating in research organizations/networks  • Disseminating research-related information  Literature (proposed):  • Participate in all decisions about research on rare diseases  • Participate in decision-making processes within research collaborations/networks  Webinar (existing):  • Funding research  **Patient reported outcome measures (PROMs)**  Literature (existing):  • Submitting PROs in a study  • Participating in studies to develop and validate PROMs  • Assisting researchers in conducting studies to develop and validate PROMs  **Patient registries and biorepositories**  Literature (existing):  • Enrolling in and submitting data to registries and biorepositories  • Providing input on the design of registries  Webinar (existing):  • Providing funding  • Encouraging others to enroll  **Stakeholder relationships and collaborations**  Webinar (existing):  • Facilitating relationships between stakeholders  • Establishing relationships with stakeholders  **Education**  Literature (existing):  • Helping to develop educational material/training programs for patients/families  Webinar (existing):  • Organizing and sponsoring formal educational activities and training programs for health care professionals, researchers, and policymakers  • Participating in standardized patient programs  **Advocacy and Awareness**  Literature (existing):  • Advocating for drug access/coverage  Webinar (existing):  • Advocating for research  • Starting awareness campaigns (e.g., fundraisers)  **Conferences and workshops**  Literature (existing):  • Participating in conferences and workshops  Webinar (existing):  • Presenting at conferences  • Sitting on organizing committees  **Patient care and support**  Literature (existing):  • Providing social support  • Monitoring own clinical care  Webinar (existing):  • Providing clinical care support  **Patient organization development**  Literature (existing):  • Establishing patient organizations  **Regulatory decision-making**  Literature (existing):  • Reporting adverse events | **Research**  Literature (existing):  • Initiating research studies  • Providing assistance to researchers conducting studies  • Leading research  • Participating in research organizations/networks  • Disseminating research-related information  Literature (proposed):  • Participate in all decisions about research on rare diseases  • Participate in decision-making processes within research collaborations/networks  Webinar (existing):  • Funding research  **Patient registries and biorepositories**  Literature (existing):  • Submitting data to registries and biorepositories on behalf of a patient  • Providing input on the design of registries  Webinar (existing):  • Providing funding  • Encouraging others to enroll  **Stakeholder relationships and collaborations**  Webinar (existing):  • Facilitating relationships between stakeholders  • Establishing relationships with stakeholders  **Education**  Literature (existing):  • Helping to develop educational material/training programs for patients/families  Webinar (existing):  • Organizing and sponsoring formal educational activities and training programs for health care professionals, researchers, and policymakers  • Participating in standardized patient programs  **Advocacy and Awareness**  Literature (existing):  • Advocating for drug access/coverage  Webinar (existing):  • Advocating for research  • Starting awareness campaigns (e.g., fundraisers)  **Conferences and workshops**  Literature (existing):  • Participating in conferences and workshops  Webinar (existing):  • Volunteering at conferences  **Patient care and support**  Literature (existing):  • Providing social support  **Patient organization development**  Literature (existing):  • Establishing patient organizations | **Research**  Literature (existing):  • Participating as subjects in studies outside of clinical trials  • Setting research priorities  • Initiating research studies  • Providing assistance to researchers conducting studies  • Leading research  • Funding research  • Participating in research organizations/networks  • Disseminating research-related information  **Patient reported outcome measures**  Literature (existing):  • Assisting researchers in conducting studies to develop and validate PROMs  **Patient registries and biorepositories**  Literature (existing):  • Providing input on the design of registries  • Serving on oversight committees  • Providing funding  • Recruiting participants  Webinar (existing):  • Encouraging enrollment in industry-led post-market studies  • Requesting data from industry registries to conduct research and share with disease community  **Stakeholder relationships and collaborations**  Literature (existing):  • Facilitating relationships between stakeholders  • Establishing relationships with stakeholders  **Education**  Literature (existing):  • Sharing informational resources on disease-specific topics  • Organizing and sponsoring formal educational activities and training programs for health care professionals, researchers, and policymakers  • Starting awareness campaigns  **Advocacy and Awareness**  Literature (existing):  • Advocating for drug access/coverage  • Advocating for research  • Advocating for legislation  Webinar (existing):  • Advocating for improved quality of life  • Advocating for education  • Advocating for clinics and patient care services  • Facilitating patients’ individual Advocacy and Awareness efforts  **Conferences and workshops**  Literature (existing):  • Participating in conferences and workshops  • Organizing conferences  • Funding conferences  **Patient care and support**  Literature (existing):  • Providing social support  • Providing financial support  • Providing clinical care support  **Patient organization development**  Literature (existing):  • Providing advice on develop patient organizations  • Fundraising to support further development of the organization |
| **Reimbursement decision-making** | **Research**  Literature (existing):  • Participating as subjects in studies outside of clinical trials  • Setting research priorities  • Initiating research studies  • Providing assistance to researchers conducting studies  • Leading research  • Participating in research organizations/networks  • Disseminating research-related information  Literature (proposed):  • Participate in all decisions about research on rare diseases  • Participate in decision-making processes within research collaborations/networks  Webinar (existing):  • Funding research  **Patient reported outcome measures (PROMs)**  Literature (existing):  • Submitting PROs in a study  • Participating in studies to develop and validate PROMs  • Assisting researchers in conducting studies to develop and validate PROMs  **Patient registries and biorepositories**  Literature (existing):  • Enrolling in and submitting data to registries and biorepositories  • Providing input on the design of registries  Webinar (existing):  • Providing funding  • Encouraging others to enroll  **Stakeholder relationships and collaborations**  Webinar (existing):  • Facilitating relationships between stakeholders  • Establishing relationships with stakeholders  **Education**  Literature (existing):  • Helping to develop educational material/training programs for patients/families  Webinar (existing):  • Organizing and sponsoring formal educational activities and training programs for health care professionals, researchers, and policymakers  • Participating in standardized patient programs  **Advocacy and Awareness**  Literature (existing):  • Advocating for drug access/coverage  Webinar (existing):  • Advocating for research  • Starting awareness campaigns (e.g., fundraisers)  **Conferences and workshops**  Literature (existing):  • Participating in conferences and workshops  Webinar (existing):  • Presenting at conferences  • Sitting on organizing committees  **Patient care and support**  Literature (existing):  • Providing social support  • Monitoring own clinical care  Webinar (existing):  • Providing clinical care support  **Patient organization development**  Literature (existing):  • Establishing patient organizations  **Regulatory decision-making**  Literature (existing):  • Reporting adverse events  **Reimbursement decision-making**  Literature (existing):  • Submitting topics for evaluation  • Providing information for preparation of evaluation report  • Directly consulted during review process  • Membership on advisory/decision-making committee  • Providing feedback on evaluation report and/or proposed recommendations  • Preparing a patient submission  • Presenting views during committee meeting  Literature (proposed):  • Providing input on benefits and risks during topic selection by horizon scanning producing organizations  • Providing input at the earliest stages of the HTA process  • Presenting a summary of patient submissions to reimbursement evaluation committees  • Consulting in the outlining of post-evaluation research recommended by reimbursement decision-makers  • Provide input into any re-assessments of benefits and risks | **Research**  Literature (existing):  • Initiating research studies  • Providing assistance to researchers conducting studies  • Leading research  • Participating in research organizations/networks  • Disseminating research-related information  Literature (proposed):  • Participate in all decisions about research on rare diseases  • Participate in decision-making processes within research collaborations/networks  Webinar (existing):  • Funding research  **Patient registries and biorepositories**  Literature (existing):  • Submitting data to registries and biorepositories on behalf of a patient  • Providing input on the design of registries  Webinar (existing):  • Providing funding  • Encouraging others to enroll  **Stakeholder relationships and collaborations**  Webinar (existing):  • Facilitating relationships between stakeholders  • Establishing relationships with stakeholders  **Education**  Literature (existing):  • Helping to develop educational material/training programs for patients/families  Webinar (existing):  • Organizing and sponsoring formal educational activities and training programs for health care professionals, researchers, and policymakers  • Participating in standardized patient programs  **Advocacy and Awareness**  Literature (existing):  • Advocating for drug access/coverage  Webinar (existing):  • Advocating for research  • Starting awareness campaigns (e.g., fundraisers)  **Conferences and workshops**  Literature (existing):  • Participating in conferences and workshops  Webinar (existing):  • Volunteering at conferences  **Patient care and support**  Literature (existing):  • Providing social support  **Patient organization development**  Literature (existing):  • Establishing patient organizations | **Research**  Literature (existing):  • Participating as subjects in studies outside of clinical trials  • Setting research priorities  • Initiating research studies  • Providing assistance to researchers conducting studies  • Leading research  • Funding research  • Participating in research organizations/networks  • Disseminating research-related information  **Patient reported outcome measures**  Literature (existing):  • Assisting researchers in conducting studies to develop and validate PROMs  **Patient registries and biorepositories**  Literature (existing):  • Providing input on the design of registries  • Serving on oversight committees  • Providing funding  • Recruiting participants  Webinar (existing):  • Encouraging enrollment in industry-led post-market studies  • Requesting data from industry registries to conduct research and share with disease community  **Stakeholder relationships and collaborations**  Literature (existing):  • Facilitating relationships between stakeholders  • Establishing relationships with stakeholders  **Education**  Literature (existing):  • Sharing informational resources on disease-specific topics  • Organizing and sponsoring formal educational activities and training programs for health care professionals, researchers, and policymakers  • Starting awareness campaigns  **Advocacy and Awareness**  Literature (existing):  • Advocating for drug access/coverage  • Advocating for research  • Advocating for legislation  Webinar (existing):  • Advocating for improved quality of life  • Advocating for education  • Advocating for clinics and patient care services  • Facilitating patients’ individual Advocacy and Awareness efforts  **Conferences and workshops**  Literature (existing):  • Participating in conferences and workshops  • Organizing conferences  • Funding conferences  **Patient care and support**  Literature (existing):  • Providing social support  • Providing financial support  • Providing clinical care support  **Patient organization development**  Literature (existing):  • Providing advice on develop patient organizations  • Fundraising to support further development of the organization  **Reimbursement decision-making**  Literature (existing):  • Submitting topics for evaluation  • Directly consulted during review process  • Membership on advisory/decision-making committee  • Preparing a patient submission  Literature (proposed):  • Consulting in topic identification and selection  • Assuring reimbursement of off-label drug use |
| **Routine clinical use** | **Research**  Literature (existing):  • Participating as subjects in studies outside of clinical trials  • Setting research priorities  • Initiating research studies  • Providing assistance to researchers conducting studies  • Leading research  • Participating in research organizations/networks  • Disseminating research-related information  Literature (proposed):  • Participate in all decisions about research on rare diseases  • Participate in decision-making processes within research collaborations/networks  Webinar (existing):  • Funding research  **Patient reported outcome measures (PROMs)**  Literature (existing):  • Submitting PROs in a study  • Participating in studies to develop and validate PROMs  • Assisting researchers in conducting studies to develop and validate PROMs  **Patient registries and biorepositories**  Literature (existing):  • Enrolling in and submitting data to registries and biorepositories  • Providing input on the design of registries  Webinar (existing):  • Providing funding  • Encouraging others to enroll  **Stakeholder relationships and collaborations**  Webinar (existing):  • Facilitating relationships between stakeholders  • Establishing relationships with stakeholders  **Education**  Literature (existing):  • Helping to develop educational material/training programs for patients/families  Webinar (existing):  • Organizing and sponsoring formal educational activities and training programs for health care professionals, researchers, and policymakers  • Participating in standardized patient programs  **Advocacy and Awareness**  Literature (existing):  • Advocating for drug access/coverage  Webinar (existing):  • Advocating for research  • Starting awareness campaigns (e.g., fundraisers)  **Conferences and workshops**  Literature (existing):  • Participating in conferences and workshops  Webinar (existing):  • Presenting at conferences  • Sitting on organizing committees  **Patient care and support**  Literature (existing):  • Providing social support  • Monitoring own clinical care  Webinar (existing):  • Providing clinical care support  **Patient organization development**  Literature (existing):  • Establishing patient organizations  **Regulatory decision-making**  Literature (existing):  • Reporting adverse events | **Research**  Literature (existing):  • Initiating research studies  • Providing assistance to researchers conducting studies  • Leading research  • Participating in research organizations/networks  • Disseminating research-related information  Literature (proposed):  • Participate in all decisions about research on rare diseases  • Participate in decision-making processes within research collaborations/networks  Webinar (existing):  • Funding research  **Patient registries and biorepositories**  Literature (existing):  • Submitting data to registries and biorepositories on behalf of a patient  • Providing input on the design of registries  Webinar (existing):  • Providing funding  • Encouraging others to enroll  **Stakeholder relationships and collaborations**  Webinar (existing):  • Facilitating relationships between stakeholders  • Establishing relationships with stakeholders  **Education**  Literature (existing):  • Helping to develop educational material/training programs for patients/families  Webinar (existing):  • Organizing and sponsoring formal educational activities and training programs for health care professionals, researchers, and policymakers  • Participating in standardized patient programs  **Advocacy and Awareness**  Literature (existing):  • Advocating for drug access/coverage  Webinar (existing):  • Advocating for research  • Starting awareness campaigns (e.g., fundraisers)  **Conferences and workshops**  Literature (existing):  • Participating in conferences and workshops  Webinar (existing):  • Volunteering at conferences  **Patient care and support**  Literature (existing):  • Providing social support  **Patient organization development**  Literature (existing):  • Establishing patient organizations | **Research**  Literature (existing):  • Participating as subjects in studies outside of clinical trials  • Setting research priorities  • Initiating research studies  • Providing assistance to researchers conducting studies  • Leading research  • Funding research  • Participating in research organizations/networks  • Disseminating research-related information  **Patient reported outcome measures**  Literature (existing):  • Assisting researchers in conducting studies to develop and validate PROMs  **Patient registries and biorepositories**  Literature (existing):  • Providing input on the design of registries  • Serving on oversight committees  • Providing funding  • Recruiting participants  Webinar (existing):  • Encouraging enrollment in industry-led post-market studies  • Requesting data from industry registries to conduct research and share with disease community  **Stakeholder relationships and collaborations**  Literature (existing):  • Facilitating relationships between stakeholders  • Establishing relationships with stakeholders  **Education**  Literature (existing):  • Sharing informational resources on disease-specific topics  • Organizing and sponsoring formal educational activities and training programs for health care professionals, researchers, and policymakers  • Starting awareness campaigns  **Advocacy and Awareness**  Literature (existing):  • Advocating for drug access/coverage  • Advocating for research  • Advocating for legislation  Webinar (existing):  • Advocating for improved quality of life  • Advocating for education  • Advocating for clinics and patient care services  • Facilitating patients’ individual Advocacy and Awareness efforts  **Conferences and workshops**  Literature (existing):  • Participating in conferences and workshops  • Organizing conferences  • Funding conferences  **Patient care and support**  Literature (existing):  • Providing social support  • Providing financial support  • Providing clinical care support  **Patient organization development**  Literature (existing):  • Providing advice on develop patient organizations  • Fundraising to support further development of the organization |
| **Obsolescence/ replacement with a new therapy** | **Research**  Literature (existing):  • Participating as subjects in studies outside of clinical trials  • Setting research priorities  • Initiating research studies  • Providing assistance to researchers conducting studies  • Leading research  • Participating in research organizations/networks  • Disseminating research-related information  Literature (proposed):  • Participate in all decisions about research on rare diseases  • Participate in decision-making processes within research collaborations/networks  Webinar (existing):  • Funding research  **Patient reported outcome measures (PROMs)**  Literature (existing):  • Submitting PROs in a study  • Participating in studies to develop and validate PROMs  • Assisting researchers in conducting studies to develop and validate PROMs  **Patient registries and biorepositories**  Literature (existing):  • Enrolling in and submitting data to registries and biorepositories  • Providing input on the design of registries  Webinar (existing):  • Providing funding  • Encouraging others to enroll  **Stakeholder relationships and collaborations**  Webinar (existing):  • Facilitating relationships between stakeholders  • Establishing relationships with stakeholders  **Education**  Literature (existing):  • Helping to develop educational material/training programs for patients/families  Webinar (existing):  • Organizing and sponsoring formal educational activities and training programs for health care professionals, researchers, and policymakers  • Participating in standardized patient programs  **Advocacy and Awareness**  Literature (existing):  • Advocating for drug access/coverage  Webinar (existing):  • Advocating for research  • Starting awareness campaigns (e.g., fundraisers)  **Conferences and workshops**  Literature (existing):  • Participating in conferences and workshops  Webinar (existing):  • Presenting at conferences  • Sitting on organizing committees  **Patient care and support**  Literature (existing):  • Providing social support  • Monitoring own clinical care  Webinar (existing):  • Providing clinical care support  **Patient organization development**  Literature (existing):  • Establishing patient organizations  **Regulatory decision-making**  Literature (existing):  • Reporting adverse events  **Reimbursement decision-making**  Literature (existing):  • Providing information for preparation of evaluation report  • Directly consulted during review process  • Membership on advisory/decision-making committee  • Providing feedback on evaluation report and/or proposed recommendations  • Preparing a patient submission  • Presenting views during committee meeting  Literature (proposed):  • Providing input at the earliest stages of the HTA process  • Presenting a summary of patient submissions to reimbursement evaluation committees  • Provide input into any re-assessments of benefits and risks | **Research**  Literature (existing):  • Initiating research studies  • Providing assistance to researchers conducting studies  • Leading research  • Participating in research organizations/networks  • Disseminating research-related information  Literature (proposed):  • Participate in all decisions about research on rare diseases  • Participate in decision-making processes within research collaborations/networks  Webinar (existing):  • Funding research  **Patient registries and biorepositories**  Literature (existing):  • Submitting data to registries and biorepositories on behalf of a patient  • Providing input on the design of registries  Webinar (existing):  • Providing funding  • Encouraging others to enroll  **Stakeholder relationships and collaborations**  Webinar (existing):  • Facilitating relationships between stakeholders  • Establishing relationships with stakeholders  **Education**  Literature (existing):  • Helping to develop educational material/training programs for patients/families  Webinar (existing):  • Organizing and sponsoring formal educational activities and training programs for health care professionals, researchers, and policymakers  • Participating in standardized patient programs  **Advocacy and Awareness**  Literature (existing):  • Advocating for drug access/coverage  Webinar (existing):  • Advocating for research  • Starting awareness campaigns (e.g., fundraisers)  **Conferences and workshops**  Literature (existing):  • Participating in conferences and workshops  Webinar (existing):  • Volunteering at conferences  **Patient care and support**  Literature (existing):  • Providing social support  **Patient organization development**  Literature (existing):  • Establishing patient organizations | **Research**  Literature (existing):  • Participating as subjects in studies outside of clinical trials  • Setting research priorities  • Initiating research studies  • Providing assistance to researchers conducting studies  • Leading research  • Funding research  • Participating in research organizations/networks  • Disseminating research-related information  **Patient reported outcome measures**  Literature (existing):  • Assisting researchers in conducting studies to develop and validate PROMs  **Patient registries and biorepositories**  Literature (existing):  • Providing input on the design of registries  • Serving on oversight committees  • Providing funding  • Recruiting participants  Webinar (existing):  • Encouraging enrollment in industry-led post-market studies  • Requesting data from industry registries to conduct research and share with disease community  **Stakeholder relationships and collaborations**  Literature (existing):  • Facilitating relationships between stakeholders  • Establishing relationships with stakeholders  **Education**  Literature (existing):  • Sharing informational resources on disease-specific topics  • Organizing and sponsoring formal educational activities and training programs for health care professionals, researchers, and policymakers  • Starting awareness campaigns  **Advocacy and Awareness**  Literature (existing):  • Advocating for drug access/coverage  • Advocating for research  • Advocating for legislation  Webinar (existing):  • Advocating for improved quality of life  • Advocating for education  • Advocating for clinics and patient care services  • Facilitating patients’ individual Advocacy and Awareness efforts  **Conferences and workshops**  Literature (existing):  • Participating in conferences and workshops  • Organizing conferences  • Funding conferences  **Patient care and support**  Literature (existing):  • Providing social support  • Providing financial support  • Providing clinical care support  **Patient organization development**  Literature (existing):  • Providing advice on develop patient organizations  • Fundraising to support further development of the organization  **Reimbursement decision-making**  Literature (existing):  • Directly consulted during review process  • Membership on advisory/decision-making committee  • Preparing a patient submission  Literature (proposed):  • Assuring reimbursement of off-label drug use |

**Figure D-1. Themes from opportunities for patients identified in the literature/website review mapped onto the orphan drug lifecycle vs. additional opportunities identified in the webinar mapped onto the orphan drug lifecycle.**


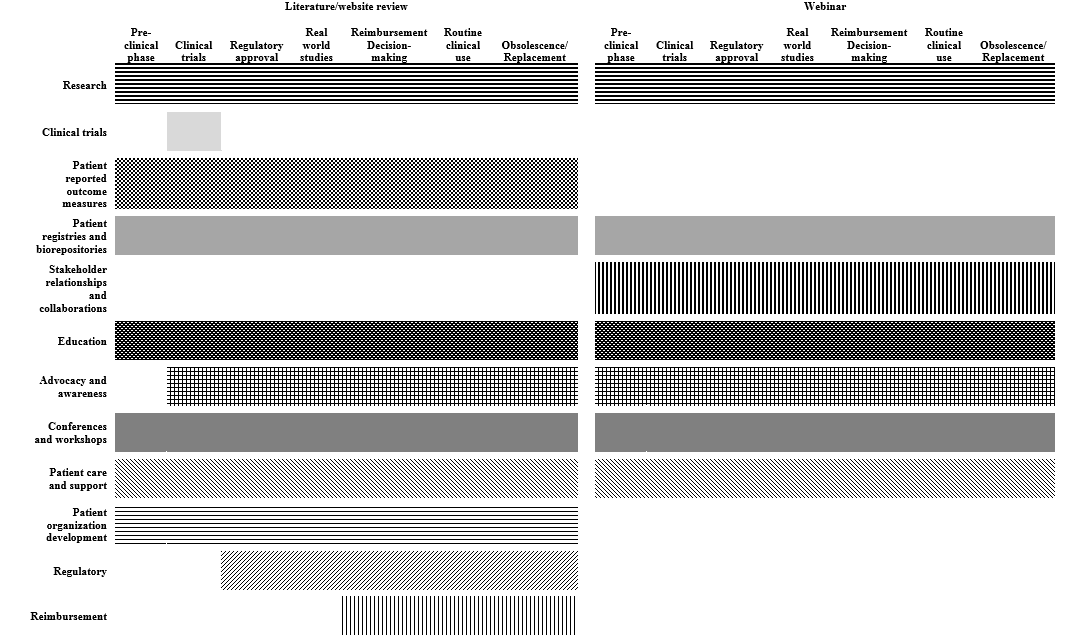


**Figure D-2. Themes from opportunities for families identified in the literature/website review mapped onto the orphan drug lifecycle vs. additional opportunities identified in the webinar mapped onto the orphan drug lifecycle.**

**
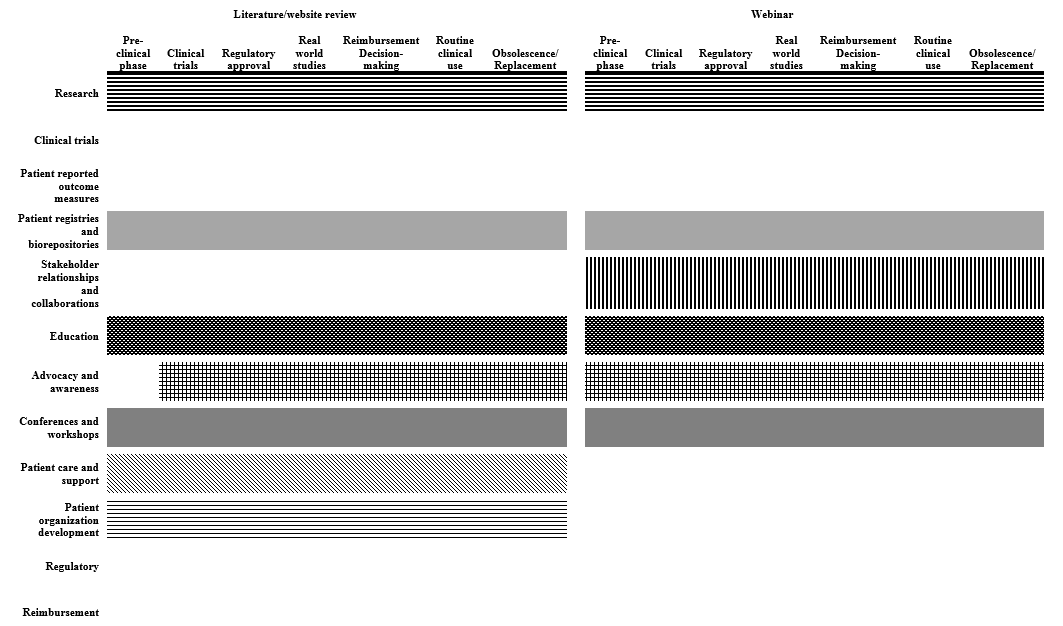
**

**Figure D-3. Themes from opportunities for patient organizations identified in the literature/website review mapped onto the orphan drug lifecycle vs. additional opportunities identified in the webinar mapped onto the orphan drug lifecycle.**


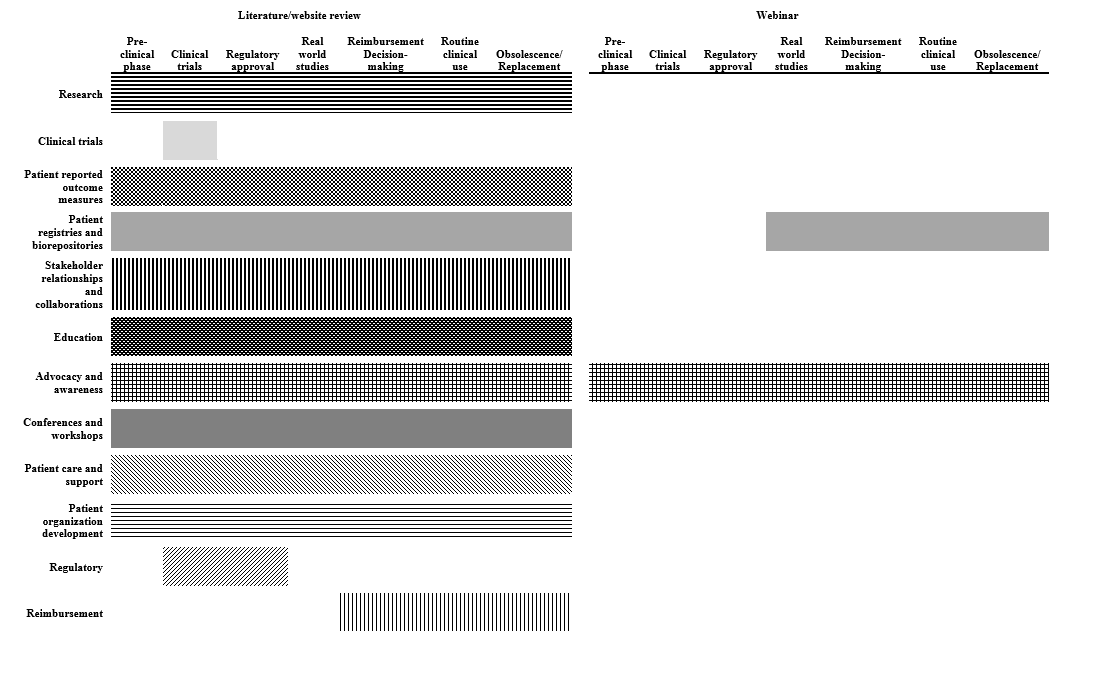

Supplement: Supplementary file 4 — Appendix D contains figures depicting the identified opportunities for patients, families, and patient organizations mapped onto the orphan drug lifecycle. (DOCX 147 kb) [file 13023_2017_738_MOESM4_ESM.docx]
